# Addressing Care Gaps in Chronic Disease Management: A Systematic Review of Nursing Interventions

**DOI:** 10.3390/healthcare14203534

**Published:** 2026-10-20

**Authors:** Barbara Cussigh, Lucija Gosak, Tamara Trajbarič, Adrijana Svenšek

**Affiliations:** Faculty of Health Sciences, University of Maribor, 2000 Maribor, Slovenia; lucija.gosak2@um.si (L.G.); tamara.trajbaric1@um.si (T.T.); adrijana.svensek1@um.si (A.S.)

**Keywords:** chronic disease management, nursing interventions, care gaps, multimorbidity, case management, continuity of care, self-management support, telehealth

## Abstract

**Background/Objectives**: Chronic diseases are a major contributor to the global burden of disease and are often associated with care gaps that adversely affect health outcomes and healthcare quality. Nurses play a central role in delivering interventions to address these gaps. This systematic review aimed to identify which nursing interventions address care gaps in chronic disease management and to assess the outcomes of their implementation among adults with chronic diseases. **Methods**: A systematic review was conducted in accordance with PRISMA guidelines. Five electronic databases were searched for studies. Studies involving adults with chronic diseases that evaluated nursing interventions targeting care gaps were eligible for inclusion. Methodological quality was assessed using JBI critical appraisal tools appropriate for the study design, and studies were not excluded based on quality assessment. Data from primary studies and systematic reviews were synthesized narratively and separately to avoid double counting of evidence. Overlap between included systematic reviews and primary studies was assessed and documented. **Results**: Twenty reports were included and synthesized separately: 17 reports of 16 primary studies (randomized and cluster-randomized controlled trials, quasi-experimental studies, and one cohort study) and three systematic reviews with meta-analysis. In the primary studies, the most frequently targeted care gap was continuity of care, followed by unmet needs, fragmented care, and health literacy or information gaps. The most common interventions were nurse-led case management, telehealth follow-up, and self-management support. Reported improvements, mostly in outcomes related to rather than direct measures of the targeted gaps, included care coordination, self-management behaviors, treatment adherence, quality of life, patient satisfaction, and selected clinical outcomes. Of the 17 primary-study reports, eight showed a statistically significant between-group benefit on the primary outcome, and four showed mixed effects; the remainder showed non-significant, within-group-only, or uncontrolled changes or did not test effectiveness. The three systematic reviews of transitional care and nurse-managed protocols reported reductions in readmissions and mortality and modest improvements in clinical risk factors. **Conclusions**: Nurse-led interventions are frequently designed to target care gaps in chronic disease management and have been associated with improvements in selected patient, service, and clinical outcomes. However, few studies measured the targeted gaps directly, so evidence that these gaps actually narrowed remains limited. Case management, telehealth follow-up, and self-management support were the most frequently evaluated approaches and warrant rigorous evaluation using direct measures of care gaps.

## 1. Introduction

Chronic diseases are among the leading causes of morbidity, mortality, and healthcare utilization worldwide [1]. As populations age and life expectancy rises, the prevalence of multimorbidity is increasing, creating additional challenges for healthcare delivery and long-term disease management [2,3]. Chronic diseases remain a major contributor to the global burden of disease. As survival continues to improve worldwide, healthcare systems are increasingly challenged not only to extend life expectancy but also to reduce years lived with poor health and disability [4].

Despite advances in chronic disease management, many patients still face care gaps that undermine health outcomes and the quality of care [5]. These gaps may include fragmented service delivery [6], limited access to healthcare [7], unmet supportive care needs, insufficient health literacy, inadequate self-management support, and deficiencies in continuity and quality of care [8]. Individuals living with multimorbidity are particularly vulnerable because healthcare systems and clinical guidelines are often organized around single diseases rather than complex, long-term health needs [7].

Nurses play a central role in addressing these challenges across primary, community, outpatient, and transitional care settings through patient education, care coordination, health promotion, and self-management support [9]. Nursing interventions, including case management, transitional care, self-management education, health coaching, telehealth follow-up, psychosocial support, and nurse-led clinics, have increasingly been implemented to improve care coordination, strengthen patient engagement, enhance access to healthcare services, and support self-management of chronic conditions [10]. Prior evidence suggests that these interventions may improve clinical outcomes, treatment adherence, patient satisfaction, and healthcare utilization [11,12].

Numerous studies have demonstrated the effectiveness of nursing interventions in improving outcomes for people with chronic diseases. However, these interventions have typically been evaluated within specific patient populations or disease contexts, while less attention has been paid to the care gaps they are intended to address. As a result, there is limited synthesis of which nursing interventions are used to address particular care gaps and which outcomes are associated with their implementation across chronic disease populations. This systematic review aimed to examine the impact of nursing interventions on addressing care gaps and improving outcomes among adults with chronic diseases.

## 2. Materials and Methods

The review followed a structured narrative systematic synthesis approach, incorporating heterogeneous evidence types (quantitative, qualitative, mixed-methods, and intervention studies, as well as systematic reviews synthesizing primary research evidence). Primary studies and systematic reviews were synthesized separately to minimize the risk of double-counting participants and study findings. Systematic reviews were used primarily to contextualize and compare findings from the included primary studies rather than to provide additional participant-level evidence. The review was reported in accordance with the Preferred Reporting Items for Systematic Reviews and Meta-Analyses (PRISMA) guidelines [13], and the synthesis was adapted to accommodate methodological diversity and aligned with a narrative systematic synthesis framework. A PRISMA flow diagram documented the study selection process, and the PRISMA checklist is provided in the Supplementary Table S5 [13]. The research question, based on the PIO approach [14], was: What is the impact of nursing interventions (I) on addressing care gaps and improving outcomes (O) in adults with chronic diseases (P)?

The review was registered in the International Prospective Register of Systematic Reviews (PROSPERO) on 5 August 2026 (CRD420261452328). Registration was retrospective with respect to the literature search, which was completed on 15 July 2026; the review start date recorded in PROSPERO (27 July 2026) refers to the start of the review process after the internal protocol had been finalised. An internal review protocol, specifying the review question, eligibility criteria, outcomes of interest, screening procedures, quality appraisal methods, data extraction procedures, and planned synthesis approach, and documenting the search strategy already executed, was prepared on 21 July 2026, before study selection commenced. At the time of registration, title-and-abstract screening, full-text eligibility assessment, data extraction, quality appraisal, and data synthesis had not yet started. Several methods reported here differ from those recorded in PROSPERO; these deviations and the reasons for them are described in Section 2.6. The retrospective registration is acknowledged as a limitation (Section 4).

### 2.1. Search Strategy

A comprehensive literature search was conducted across five international databases: PubMed (U.S. National Library of Medicine), MEDLINE and CINAHL Ultimate (both via EBSCOhost, EBSCO Information Services, 10 Estes Street, Ipswich, MA 01938, USA), Web of Science Core Collection (Clarivate, 70 St. Mary Axe, London, EC3A 8BE, Great Britain), and Scopus (Elsevier, Radarweg 29, 1043 NX Amsterdam, Netherlands) (up to 15 July 2026) to identify studies examining the impact of nursing practice on addressing care gaps and improving outcomes for adults with chronic diseases.

The search used free-text keywords covering chronic diseases, nursing practice, nursing interventions, care gaps, continuity of care, unmet needs, quality of care, self-management, and patient outcomes. No controlled-vocabulary terms (e.g., MeSH or CINAHL Subject Headings) were added to the search string. Boolean operators (AND, OR) were used to combine search terms. No publication date restrictions were applied, and only studies published in English were included. The database searches yielded 189 records from PubMed, 257 from MEDLINE, 301 from CINAHL Ultimate, 228 from Web of Science, and 213 from Scopus prior to deduplication. The same search string was used in all databases, except in PubMed, where it was applied without truncation symbols (asterisks): (“chronic disease*” OR “chronic illness*” OR “chronic condition*” OR “long-term condition*” OR “noncommunicable disease*” OR multimorbid* OR comorbid*) AND (“nursing care” OR “nursing practice” OR “nursing intervention*” OR “nursing management” OR “nurse-led” OR “nurse-managed”) AND (“care gap*” OR “gap* in care” OR “unmet need*” OR “unmet healthcare need*” OR “unmet health care need*” OR “missed care” OR “care deficit*” OR “fragmented care” OR “continuity of care” OR “access to care” OR “barrier* to care” OR “quality of care”) AND (“patient outcome*” OR “quality of life” OR “quality of care” OR satisfaction OR self-management OR “self-management”). The complete strategies as executed in each database, including the platform, searched fields, applied limits, date of the final search, and number of records retrieved, are provided in Supplementary Table S7.

Screening was managed using Rayyan [15]. All records retrieved from the databases were imported into the platform and checked for duplicates before relevance screening. The screening process comprised two stages: title-and-abstract screening, followed by full-text review of potentially eligible studies. Screening was conducted independently by two reviewers. Any disagreements were resolved through discussion and, when necessary, consultation with an additional reviewer. Studies whose eligibility could not be determined from the title and abstract were retained for full-text assessment to minimize the risk of premature exclusion. In addition, the reference lists of all included studies were manually screened to identify any additional relevant studies that may not have been captured through the database search. No additional studies were identified through reference-list screening.

### 2.2. Eligibility Criteria

Studies were eligible for inclusion if they were published in English, involved adults (≥18 years) with one or more chronic diseases, and examined nursing care, nursing interventions, or nursing management strategies. For this review, nursing interventions were defined as those led, coordinated, or primarily delivered by registered nurses, advanced practice nurses, community nurses, or other qualified nursing professionals. Multidisciplinary interventions were eligible if nurses had a clearly defined and substantial role in intervention delivery, care coordination, patient education, case management, follow-up, or self-management support. To be included, studies also had to address care gaps, unmet needs, or deficiencies in chronic disease management and report patient- or care-related outcomes, such as quality of life, self-management, patient satisfaction, continuity of care, access to care, or quality of care. Eligible study designs included quantitative, qualitative, and mixed-methods studies, intervention studies, and systematic reviews or meta-analyses synthesizing evidence on nursing interventions in chronic disease management.

Studies were excluded if they focused on pediatric populations, addressed conditions not classified as chronic diseases, or evaluated interventions without a nursing component. Studies addressing exclusively medical or pharmacological treatment were also excluded. In addition, editorials, opinion papers, commentaries, conference abstracts, protocols, narrative reviews, scoping reviews, non-full-text articles, and studies not published in English were excluded from the review. Theses, dissertations, and other non-peer-reviewed reports were also excluded. The inclusion and exclusion criteria used in this review are presented in Table 1.

### 2.3. Data Extraction

Data extraction was conducted independently by two reviewers using a standardized form developed in advance and aligned with the review question and the structure of the study characteristics table. The following data were extracted from each included study: author and year of publication, country, healthcare setting, study design, chronic condition(s) addressed, sample size, type of nursing intervention, primary care gap targeted, whether and how the targeted care gap was measured, and direction of the reported effect. When relevant information was unavailable, it was recorded as “not reported.” The extracted data were checked for completeness and consistency. Any discrepancies or conflicts arising during the review and data-extraction process were resolved through discussion and consensus; when consensus could not be reached, a third reviewer was consulted. The characteristics of the included primary studies and systematic reviews are presented in Table 2a and Table 2b, respectively. In this review, a study refers to a unique investigation and a report to a publication describing it; one study may be described in more than one report. Study characteristics (design, country, setting, and intervention type) are counted per unique study, whereas care gaps and direction of effect, which may differ between reports of the same study, are counted per report.

Care gaps were classified using a predefined taxonomy of seven categories derived from the literature on chronic care [5,6,7,8]: (1) continuity of care, meaning disruptions in informational, management, or relational continuity, particularly across care transitions; (2) fragmented care, meaning poorly coordinated care delivered by multiple providers or services; (3) health literacy or information gaps, meaning insufficient knowledge, understanding, or information needed for self-management and decision-making; (4) unmet needs, meaning physical, psychosocial, supportive, or palliative needs not addressed by existing services; (5) quality-of-care deficits, meaning shortfalls against recommended or guideline-concordant care processes; (6) care deficits, meaning insufficient self-care capacity or support for self-management; and (7) access-to-care gaps, meaning geographical, financial, temporal, or organizational barriers to obtaining care. Each report was assigned one primary category, defined as the gap explicitly identified by the study authors as the principal target of the intervention in the study aims or rationale. Where several gaps were cited, the gap most closely linked to the primary outcome was selected. Additional gaps were recorded as secondary. Category assignment was performed independently by two reviewers, and disagreements were resolved by consensus.

The care gap that an intervention was designed to address was recorded separately from evidence that the gap had narrowed. Change in the targeted gap was classified as follows. A gap was considered directly measured only when a report used an instrument or indicator that quantified the targeted primary care gap itself, as listed in Table 2a; measures of secondary gaps or of related clinical, behavioral, or utilization outcomes were not counted as direct measures. “Reduced” was assigned when a direct measure of the targeted gap (e.g., a continuity-of-care, care-coordination, access, or unmet-needs measure) showed a statistically significant between-group improvement. “Partially reduced” was assigned when a direct measure improved on some but not all indicators, when improvement was demonstrated only within groups or in uncontrolled designs, or when the between-group improvement was based only on provider perceptions or on a comparison between two active interventions without a usual-care comparator. “No change” was assigned when a direct measure showed no significant difference. “Not directly measured” was assigned when the gap itself was not assessed and only related clinical, behavioral, or utilization outcomes were reported; such outcomes were treated as indirect evidence and were not classified as a reduction of the gap.

**Figure 1:**
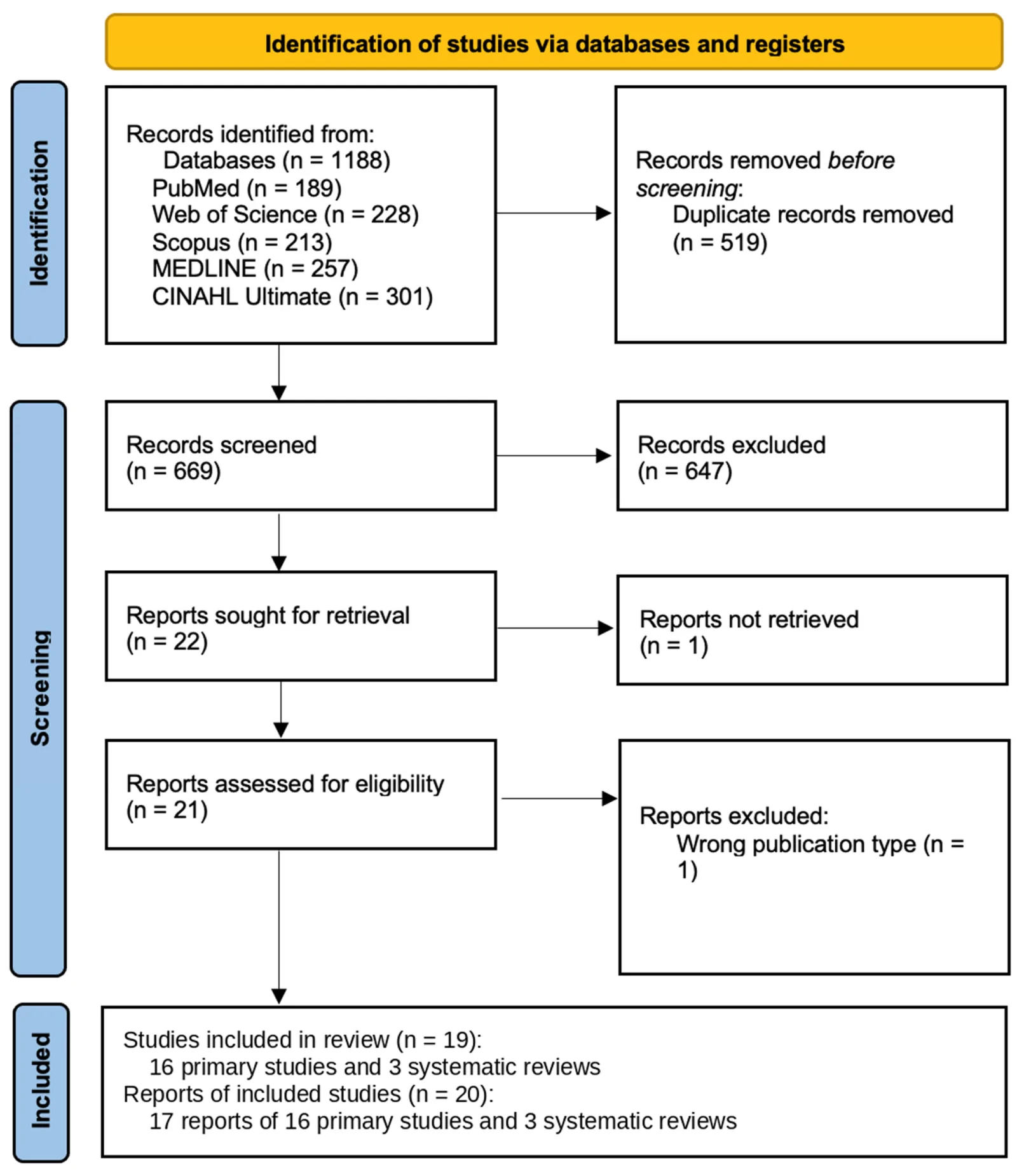
PRISMA 2020 flow diagram.

The study selection process is presented in the PRISMA 2020 flow diagram (Figure 1), outlining the number of records identified, screened, excluded, and ultimately included in the systematic review.

Because this review was based exclusively on published literature, ethical approval was not required. Informed consent was not required because no primary data were collected. No generative artificial intelligence was used for study selection, data extraction, quality assessment, or interpretation of the findings.

### 2.4. Data Synthesis

Due to heterogeneity across studies in study design, populations, chronic conditions, nursing interventions, and outcome measures, a narrative synthesis approach was used. The narrative synthesis followed the framework proposed by Popay et al. [16], involving the development of a preliminary synthesis of findings, exploration of relationships within and between studies, assessment of the robustness of the evidence, and generation of an overall interpretation of the findings. Findings were compared and summarized across studies to examine how nursing interventions address care gaps in chronic disease management and influence patient and healthcare outcomes. Particular attention was paid to the characteristics of nursing interventions, the types of care gaps addressed, and the reported effects on patient outcomes, quality of care, self-management, continuity of care, healthcare utilization, patient satisfaction, access to care, and quality of life. The direction of effect was classified on the basis of between-group comparisons with the study comparator; within-group (pre–post) changes and results from uncontrolled designs were recorded separately and were not counted as evidence of comparative benefit. The following categories were applied to the primary outcome of each report: “Positive (significant)”, a statistically significant between-group difference favoring the intervention; “Positive (trend)”, a non-significant between-group difference favoring the intervention; “Mixed”, significant between-group differences in some but not all outcomes; “No significant between-group difference”, no significant between-group differences in primary or secondary outcomes, irrespective of any within-group changes; “Within-group change only”, controlled studies that reported only pre–post changes within study arms without testing differences between groups; “Uncontrolled pre–post change”, single-group designs without a comparator; and “Not applicable”, feasibility studies that did not test effectiveness. For systematic reviews, classification was based on the pooled estimates reported for the primary outcome. Classification was performed independently by two reviewers, and disagreements were resolved by consensus. Primary studies and systematic reviews were synthesized separately, and all frequency summaries are based on primary studies only. Overlap between the included systematic reviews and primary studies was documented in a citation matrix (Supplementary Table S6) [17]. Because the complete sets of primary studies included in the three systematic reviews were not mapped, overlap among the reviews themselves was not quantified, and the overlap assessment was restricted to the primary studies included in the present review.

### 2.5. Quality Assessment

The methodological quality of the included reports was assessed using the JBI Critical Appraisal Tools appropriate for each study design. Depending on the methodological design of the included reports, the relevant JBI checklists for cohort studies [18], quasi-experimental studies [19], systematic reviews [20], and randomized controlled trials [21] were applied. Quality appraisal was performed independently by two reviewers, and disagreements were resolved through discussion or, when necessary, by a third reviewer.

For reports assessed using the JBI tools, each criterion rated “yes” received one point, whereas “no” or “unclear” responses received zero points. Criteria rated “not applicable” were excluded from both the numerator and the denominator, so that reports were not penalized for items irrelevant to their design. Total scores and percentages of applicable criteria were calculated for each report. Because summary scores weight all criteria equally and may mask critical flaws, percentage scores were used descriptively and were considered alongside item-level judgments (Table 3, Table 4, Table 5 and Table 6). Following [22], reports were categorized as low (<60%), moderate (60–79%), high (80–89%), or excellent (≥90%) in methodological quality.

The methodological quality assessment was not used as a basis for study exclusion but rather to support the interpretation and contextualization of the findings. During the synthesis process, evidence derived from reports rated as high or excellent quality was given greater weight in drawing conclusions, whereas findings from reports of lower methodological quality were interpreted with greater caution. Specifically, appraisal judgments were reported alongside each study’s findings in the narrative synthesis; findings from uncontrolled designs, or from studies with critical limitations (e.g., no between-group comparison, unaccounted clustering, or major inconsistencies in reported data), were not used as the sole basis for conclusions; and conclusions were qualified where they relied predominantly on studies rated low or moderate. Methodological quality was assessed at the study level. No formal framework for rating outcome-level certainty of evidence (e.g., GRADE) was applied, so statements about the overall strength of evidence in this review reflect study-level methodological limitations and are not formal certainty ratings.

**Table 2:** (**a**) Characteristics of the included primary studies. Direction of effect refers to the primary outcome and is classified on the basis of between-group comparisons with the study comparator: Positive (significant), statistically significant between-group benefit; Positive (trend), non-significant between-group difference favoring the intervention; Mixed, significant between-group differences in some but not all outcomes; No significant between-group difference, no significant differences in primary or secondary outcomes; Within-group change only, pre–post changes within study arms without between-group testing; Uncontrolled pre–post change, single-group design without a comparator; Not applicable, feasibility study without effectiveness testing. Only the Positive (significant) category denotes demonstrated comparative benefit. (**b**) Characteristics of the included systematic reviews.

(a)										
Report (Author, Year)	Country	Setting	Design	Chronic Condition(s)		N	Nursing Intervention Type		Care Gap (Primary)	Direction of Effect
Meekaew et al., [23]	Thailand	Outpatient clinic	Cluster RCT	T2DM + hypertension + CKD		120 (60/60)	Self-management education/support		Care deficit	Positive (significant)
Boult et al., [24] ^a^	USA	Primary care	Cluster RCT	Multimorbidity (≥65 y, high risk)		904 (485/419)	Case management		Quality of care	Positive (significant)
Schenker et al., [25]	USA	Outpatient clinic	Quasi-exp. (single-arm)	Advanced cancer		23 (single-arm)	Case management		Unmet needs	Not applicable
Leff et al., [26] ^a^	USA	Primary care	Cluster RCT	Multimorbidity (≥65 y, high risk)		904 (485/419)	Case management		Fragmented care	Positive (trend)
Rechenberg et al., [27]	USA	Primary care	RCT	Type 2 diabetes (Latinx immigrants)		18 (10/7)	Self-management education/support		Health literacy/information gap	Within-group change only (no between-group test)
Schraeder et al., [28]	USA	Primary care	Quasi-exp.	Multimorbidity (≥65 y, high risk)		677 (400/277)	Case management		Fragmented care	Mixed
Xiao et al., [29]	China	Hospital/acute care	RCT	Lung cancer (chemotherapy)		120 dyads (240)	Psychosocial support/counselling		Unmet needs	Mixed
Wang et al., [30]	China	Transitional (hospital-to-home)	Retrospective cohort	Chronic heart failure		90 (45/45)	Multicomponent		Continuity of care	Positive (significant)
Mallow et al., [31]	USA	Community	Quasi-exp. (pre/post)	Multimorbidity		30 (single-arm)	Telephone/telehealth follow-up		Access to care	Uncontrolled pre–post change
Xie et al., [32]	China	Transitional (hospital-to-home)	RCT	Chronic heart failure (older adults)		104 (52/52)	Self-management education/support		Continuity of care	Positive (significant)
Fu et al., [33]	China	Transitional (hospital-to-home)	Cluster RCT	Multimorbidity (post-discharge, older)		400 (200/200)	Telephone/telehealth follow-up		Continuity of care	Mixed
Wong et al., [34]	Hong Kong (China)	Outpatient clinic	RCT	CKD/ESRF on CAPD		98 (49/49)	Case management		Continuity of care	Mixed
Ratanawongsa et al., [35]	USA	Outpatient clinic	RCT	Type 2 diabetes (poor control)		330 (112/113/105)	Telephone/telehealth follow-up		Health literacy/information gap	Positive (significant)
Riegel et al., [36]	USA	Transitional (hospital-to-home)	Cluster RCT	Chronic heart failure		358 (130/228)	Telephone/telehealth follow-up		Continuity of care	Positive (significant)
Akkus & Akdemir, [37]	Turkey	Home care	Quasi-exp.	Multiple sclerosis		45 (21/24)	Home visits		Unmet needs	Positive (significant)
Kizawa et al., [38]	Japan	Primary care	RCT	Chronic diseases (CVD, cancer, pulmonary, renal)		220 (117/103)	Psychosocial support/counselling		Health literacy/information gap	No significant between-group difference
Nualchaem et al., [39]	Thailand	Primary care	Quasi-exp.	Diabetes and/or hypertension (multimorbidity)		60 (30/30)	Case management		Fragmented care	Positive (significant)
(**b**)										
**Review**	**Search End Date**	**Included Studies**	**Participants**	**Population**	**Nursing Intervention**	**Care Gap (Primary)**	**Main Findings**	**Direction of Effect**	**JBI Quality**	**Primary Studies of the Present Review Also Included**
Zhu et al. [40]	[29 March 2014]	10 RCTs	3438	Mixed chronic (HF, CHD, older adults)	Transitional care (early discharge planning)	Continuity of care	Reduced readmissions, readmission length of stay and mortality	Mixed	10/11 (91%), excellent	[Wong et al.] [34]
Shaw et al. [41]	[31 January 2014]	18 studies	23,004	Diabetes, hypertension, hyperlipidaemia	Nurse-led clinic/consultation (nurse-managed protocols)	Quality of care	Modest improvements in HbA1c, blood pressure and cholesterol	Mixed	10/11 (91%), excellent	[None]
Le Berre et al. [42]	[9 April 2015]	92 RCTs	[Not Reported]	Chronically ill older patients (CHF, respiratory, cancer, other)	Transitional care	Continuity of care	Reduced mortality, readmissions and readmission days; quality of life not consistently improved	Mixed	10/11 (91%), excellent	[Riegel et al.] [36]

^a^ Two reports of the same Guided Care cluster-randomized trial; counted as one study for study characteristics and as separate reports for care gap and direction of effect.

**Table 3:** Critical appraisal of randomized controlled trials, including cluster-randomized trials [21].

Checklist Question	Meekaew et al. [23]	Boult et al. [24]	Leff et al. [26]	Rechenberg et al. [27]	Xiao et al. [29]	Xie et al. [32]	Fu et al. [33]	Wong et al. [34]	Ratanawongsa et al. [35]	Riegel et al. [36]	Kizawa et al. [38]
Q1 ^1^	Unclear	Yes	Unclear	Unclear	Yes	Yes	Yes	Yes	Unclear	Unclear	Yes
Q2 ^2^	Unclear	Yes	Yes	Unclear	Yes	Unclear	Yes	Unclear	Unclear	Unclear	Unclear
Q3 ^3^	Unclear	No	No	Unclear	Yes	No	Yes	No	Unclear	No	No
Q4 ^4^	No	No	No	No	No	No	No	No	No	No	No
Q5 ^5^	No	No	No	No	No	No	No	No	No	No	No
Q6 ^6^	Unclear	Unclear	Yes	No	Yes	Yes	Yes	Yes	Yes	Yes	Yes
Q7 ^7^	Unclear	No	Unclear	No	No	Unclear	No	No	No	Unclear	No
Q8 ^8^	Yes	Yes	Yes	Yes	Yes	Yes	No	Yes	Yes	Yes	Yes
Q9 ^9^	Unclear	Unclear	Unclear	Yes	Unclear	Unclear	Yes	Yes	Unclear	Unclear	Unclear
Q10 ^10^	No	Yes	No	Unclear	Unclear	Unclear	Unclear	No	No	No	Yes
Q11 ^11^	No	Yes	No	No	Yes	Unclear	No	No	Unclear	No	Yes
Q12 ^12^	No	No	Unclear	No	Unclear	Yes	No	Yes	Unclear	No	Unclear
Q13 ^13^	No	No	Unclear	Yes	Yes	Yes	No	Yes	Yes	No	Yes
Score, n/N (%)	1/13 (8%)	5/13 (38%)	3/13 (23%)	3/13 (23%)	7/13 (54%)	5/13 (38%)	5/13 (38%)	6/13 (46%)	3/13 (23%)	2/13 (15%)	6/13 (46%)
Quality	Low	Low	Low	Low	Low	Low	Low	Low	Low	Low	Low

Note: ^1^ Was true randomization used for assignment of participants to treatment groups? ^2^ Was allocation to treatment groups concealed? ^3^ Were treatment groups similar at the baseline? ^4^ Were participants blind to treatment assignment? ^5^ Were those delivering the treatment blind to treatment assignment? ^6^ Were treatment groups treated identically other than the intervention of interest? ^7^ Were outcome assessors blind to treatment assignment? ^8^ Were outcomes measured in the same way for treatment groups? ^9^ Were outcomes measured in a reliable way? ^10^ Was follow up complete and if not, were differences between groups in terms of their follow up adequately described and analysed? ^11^ Were participants analysed in the groups to which they were randomized? ^12^ Was appropriate statistical analysis used? ^13^ Was the trial design appropriate and any deviations from the standard RCT design (individual randomization, parallel groups) accounted for in the conduct and analysis of the trial? Cluster-randomized trials: Meekaew et al. [23], Boult et al. [24], Leff et al. [26], Fu et al. [33], and Riegel et al. [36]. For these trials, Q13 was used to judge whether clustering was accounted for in the sample-size calculation and analysis, which bears on the risk of unit-of-analysis errors and unaccounted design effects. Additional cluster-specific concerns: clustering not accounted for in the sample-size calculation or analysis, Meekaew et al. [23], Boult et al. [24], Fu et al. [33], and Riegel et al. [36]; very small number of clusters, Meekaew et al. [23] and Fu et al. [33]. Results from these trials may overstate precision and should be interpreted with additional caution. Overall, retrospective propensity-score–matched cohort study has a high risk of bias and limited ability to support causal conclusions. Although the investigators recruited both groups from the same hospital population, reported complete follow-up, and used several standardized outcome instruments, important methodological weaknesses remain. The article inconsistently describes the study as both observational and randomized, provides insufficient details about exposure classification and the propensity-score matching procedure, identifies and controls confounding inadequately, and reports a statistically significant baseline age difference despite claiming group comparability. Outcome measurement procedures were incompletely described, follow-up was too short to assess longer-term clinical outcomes, and the statistical analyses did not appropriately account for repeated measurements, matched data, residual baseline differences, multiple comparisons, or sparse event counts. Consequently, the reported improvements may reflect bias, confounding, or analytical limitations rather than a reliable treatment effect, and the findings should be interpreted cautiously rather than regarded as definitive evidence of effectiveness.

**Table 4:** Critical appraisal of the cohort study [18].

Checklist Question	Wang et al. [30]
Q1 ^1^	No
Q2 ^2^	Unclear
Q3 ^3^	Unclear
Q4 ^4^	No
Q5 ^5^	Unclear
Q6 ^6^	No
Q7 ^7^	Unclear
Q8 ^8^	Unclear
Q9 ^9^	Yes
Q10 ^10^	Yes
Q11 ^11^	No
Score, n/N (%)	2/11 (18%)
Quality	Low

Note: ^1^ Were the two groups similar and recruited from the same population? ^2^ Were the exposures measured similarly to assign people to both the exposed and unexposed groups? ^3^ Was the exposure measured in a valid and reliable way? ^4^ Were confounding factors identified? ^5^ Were strategies to deal with confounding factors stated? ^6^ Were the groups/participants free of the outcome at the start of the study (or at the moment of the exposure)? ^7^ Were the outcomes measured in a valid and reliable way? ^8^ Was the follow-up time reported and sufficient to be long enough for outcomes to occur? ^9^ Was follow-up complete, and if not, were the reasons for loss to follow-up described and explored? ^10^ Were strategies to address incomplete follow-up utilized? ^11^ Was appropriate statistical analysis used?

**Table 5:** Critical appraisal of quasi-experimental studies [19].

Checklist Question	Schenker et al. [25]	Schraeder et al. [28]	Nualchaem et al. [39]	Akkuş & Akdemir [37]	Mallow et al. [31]
Q1 ^1^	Yes	Yes	Yes	Yes	Yes
Q2 ^2^	No	Yes	Yes	Yes	No
Q3 ^3^	No	No	No	Yes	No
Q4 ^4^	No	No	Unclear	Unclear	No
Q5 ^5^	No	No	No	No	No
Q6 ^6^	Yes	Yes	Yes	Yes	Unclear
Q7 ^7^	Unclear	Yes	Unclear	Unclear	Unclear
Q8 ^8^	Yes	Yes	No	No	No
Q9 ^9^	Yes	Yes	No	Unclear	Unclear
Score, n/N (%)	4/9 (44%)	6/9 (67%)	3/9 (33%)	4/9 (44%)	1/9 (11%)
Quality	Low	Moderate	Low	Low	Low

Note: ^1^ Is it clear in the study what is the “cause” and what is the “effect” (i.e., there is no confusion about which variable comes first)? ^2^ Was there a control group? ^3^ Were participants included in any comparisons similar? ^4^ Were the participants included in any comparisons receiving similar treatment/care, other than the exposure or intervention of interest? ^5^ Were there multiple measurements of the outcome, both pre and post the intervention/exposure? ^6^ Were the outcomes of participants included in any comparisons measured in the same way? ^7^ Were outcomes measured in a reliable way? ^8^ Was follow-up complete and if not, were differences between groups in terms of their follow-up adequately described and analyzed? ^9^ Was appropriate statistical analysis used?

**Table 6:** Critical appraisal of Systematic reviews.

Checklist Question	Le Berre et al. [42]	Shaw et al. [41]	Zhu et al. [40]
Q1 ^1^	Yes	Yes	Yes
Q2 ^2^	Yes	Yes	Yes
Q3 ^3^	Yes	Yes	Yes
Q4 ^4^	No	Yes	No
Q5 ^5^	Yes	Yes	Yes
Q6 ^6^	Yes	No	Yes
Q7 ^7^	Yes	Yes	Yes
Q8 ^8^	Yes	Yes	Yes
Q9 ^9^	Yes	Yes	Yes
Q10 ^10^	Yes	Yes	Yes
Q11 ^11^	Yes	Yes	Yes
Score, n/N (%)	10/11 (91%)	10/11 (91%)	10/11 (91%)
Quality	Excellent	Excellent	Excellent

Note: ^1^ Is the review question clearly and explicitly stated? ^2^ Were the inclusion criteria appropriate for the review question? ^3^ Was the search strategy appropriate? ^4^ Were the sources and resources used to search for studies adequate? ^5^ Were the criteria for appraising studies appropriate? ^6^ Was critical appraisal conducted by two or more reviewers independently? ^7^ Were there methods to minimize errors in data extraction? ^8^ Were the methods used to combine studies appropriate? ^9^ Was the likelihood of publication bias assessed? ^10^ Were recommendations for policy and/or practice supported by the reported data? ^11^ Were the specific directives for new research appropriate?

### 2.6. Deviations from the Registered Protocol

Several entries in the PROSPERO record did not correspond to the internal protocol that guided the review; the methods reported here follow the internal protocol. The differences and the reasons for them are as follows.

(1) Study design. The PROSPERO record restricted eligibility to randomized studies. However, the internal protocol specified a broader range of eligible study designs, including non-randomized intervention studies and systematic reviews. The methods reported in this review therefore follow the internal protocol rather than the PROSPERO record. To limit the effect of this difference, direction of effect was classified on the basis of between-group comparisons only, primary studies and systematic reviews were synthesized separately, and findings from uncontrolled designs were not used as the sole basis for conclusions (Section 2.4 and Section 2.5). (2) Information sources. The PROSPERO record listed Google Scholar, PubMed, and Scopus. MEDLINE, CINAHL Ultimate, and Web of Science Core Collection were added to improve coverage of the nursing literature, and Google Scholar was not searched because its results cannot be retrieved reproducibly or exported in full.

(3) Supplementary searching. The PROSPERO record specified forward citation searching. Instead, the reference lists of included studies were screened (backward citation searching), as this was considered a more feasible supplementary search method within the scope of the review. No additional eligible studies were identified. (4) Risk-of-bias assessment. The PROSPERO record specified ROBIS, which is designed for systematic reviews and is not applicable to primary studies. Design-specific JBI critical appraisal tools were therefore used for all included reports.

(5) Data synthesis. Thematic synthesis was specified in PROSPERO. Because the included evidence consisted of quantitative intervention studies and systematic reviews, with no qualitative studies, a narrative synthesis following Popay et al. [16] was used instead (Section 2.4).

(6) Risk of bias due to missing results. Although specified in PROSPERO, this was not formally assessed because the small number of heterogeneous studies per outcome precluded methods such as funnel plots. This is acknowledged as a limitation (Section 4).

(7) Contact with study authors. Study authors were not contacted regarding missing data because sufficient information was available in the published reports for eligibility assessment, data extraction, and quality appraisal; missing information was recorded as “not reported”. (8) Additional analyses. The classification of direction of effect, the classification of change in the targeted care gap, and the assessment of overlap between systematic reviews and primary studies were not specified in PROSPERO; they were introduced during peer review to improve the interpretability of the findings.

## 3. Results

### 3.1. Characteristics of the Included Studies

Twenty reports met the inclusion criteria: 17 reports of 16 primary studies and three systematic reviews with meta-analysis (Table 2a,b). Two reports (Boult et al. [24]; Leff et al. [26] derived from the same Guided Care cluster-randomized controlled trial but reported different outcome domains; they are counted as one study for study characteristics and as separate reports for care gaps and direction of effect. The primary-study reports were published between 2002 and 2026, and the 16 primary studies were conducted in six countries and regions: the USA (*n* = 7), China (*n* = 4), Thailand (*n* = 2), Hong Kong (China), Turkey, and Japan (*n* = 1 each). Detailed study identification, contextual, and population characteristics are provided in Supplementary Table S1.

Of the 16 primary studies, six were individually randomized controlled trials (RCTs), four were cluster RCTs (described in five reports), five were quasi-experimental studies (including two single-group or single-arm designs), and one was a retrospective cohort study. Care was delivered most frequently in primary care (*n* = 5), followed by transitional (hospital-to-home) care and outpatient clinics (*n* = 4 each), and hospital or acute care, community, and home care settings (*n* = 1 each).

The primary studies spanned a broad range of chronic conditions, most frequently chronic heart failure, diabetes, and multimorbidity in older adults, followed by chronic kidney disease, cancer, and multiple sclerosis. Sample sizes ranged from 18 to 904 participants. The three systematic reviews included between 10 and 92 primary studies and up to 23,004 participants (Table 2b).

### 3.2. Data Synthesis

**Figure 2:**
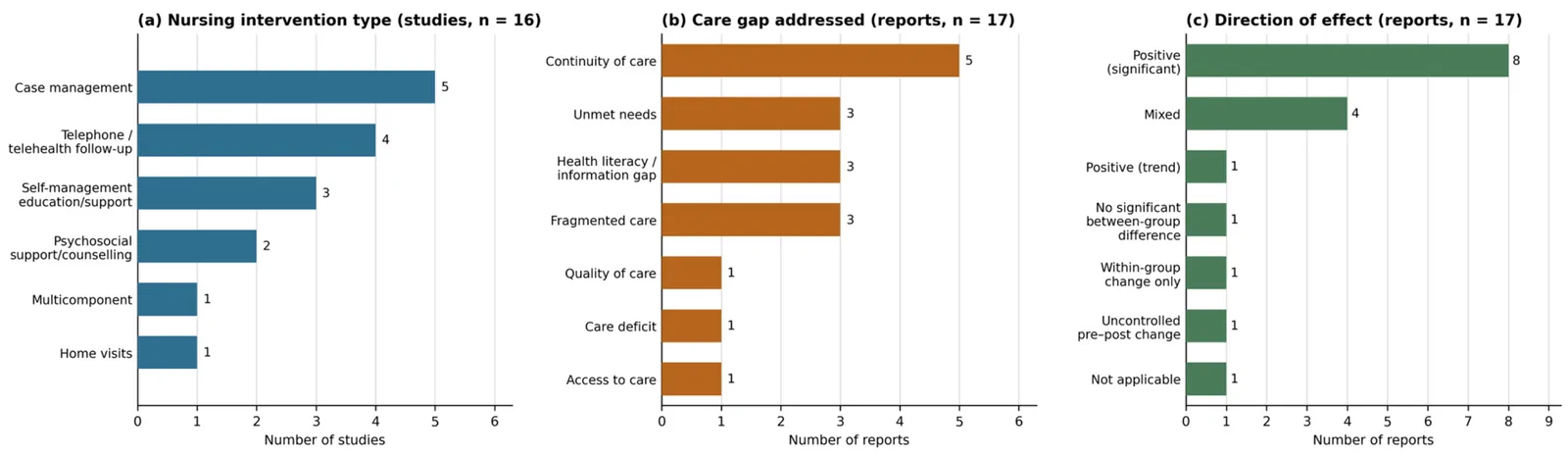
Distribution of the 17 primary-study reports (16 studies) by (**a**) nursing intervention type (counted per study), (**b**) primary care gap addressed, and (**c**) direction of the reported effect, classified on the basis of between-group comparisons (categories as defined in Table 2a). Systematic reviews are not included; they are summarized in Table 2b and Section 3.3. Where a report targeted more than one care gap, the primary gap is shown; the full mapping is provided in Supplementary Table S3.

The nursing interventions evaluated in the primary studies were heterogeneous (Figure 2a). The most common approach was nurse-led case management (*n* = 5 studies), followed by telephone or telehealth follow-up (*n* = 4) and self-management education and support (*n* = 3). Psychosocial support or counseling was evaluated in two studies, and multicomponent programs and home visits in one study each. Detailed characteristics of the nursing interventions, including intervention components, providers, duration, intensity, follow-up, and comparators, are presented in Supplementary Table S2. Interventions were most often delivered by registered nurses, advanced practice nurses, or nurse case managers, frequently as part of a multidisciplinary team.

Figure 2b summarizes the primary care gaps targeted in the 17 primary-study reports. Continuity of care was the most frequently targeted gap (*n* = 5), followed by unmet needs, fragmented care, and health-literacy or information gaps (*n* = 3 each), and quality-of-care, care-deficit, and access-to-care gaps (*n* = 1 each). Several reports addressed more than one overlapping gap; Table 2a presents the primary gap, and the full mapping is provided in the extended extraction table (Supplementary Table S3).

Regarding the direction of effect (Figure 2c), classified on the basis of between-group comparisons, eight of the 17 primary-study reports described a statistically significant between-group benefit of the nursing intervention on the primary outcome, and one additional report described a non-significant trend favoring the intervention. Four reports described mixed effects, with significant improvements in some outcomes (most commonly self-management, treatment adherence, patient satisfaction, and selected quality-of-life or clinical measures) but no significant between-group differences in others. One RCT found no significant between-group differences in either primary or secondary outcomes, and reported only within-group changes that may reflect social desirability or repeated assessment [35], and one RCT reported only within-group changes without between-group testing [27]; neither was counted as demonstrating comparative benefit. One single-group pre–post study reported changes that cannot be attributed to the intervention in the absence of a comparator [31], and a single-arm pilot [25] did not permit an estimate of effect. In three trials, the comparator was an active or attention-control condition rather than usual care (Xiao et al. [29], attention control; Fu et al. [33], standardised nurse telephone follow-up; Kizawa et al. [38], standardised nurse-led verbal advance care planning), which may have attenuated between-group differences (Supplementary Table S2). Because counts of statistically significant findings do not convey the magnitude or precision of effects, these categories are descriptive and should be interpreted alongside the outcome-specific effect estimates reported by the individual studies. Using the criteria described in Section 2.3, the targeted care gap was measured directly in 4 of the 17 primary-study reports: Meekaew et al. [23] (care deficit; self-care agency and self-care behavior scales), Boult et al. [24] (quality of care; Patient Assessment of Chronic Illness Care), Ratanawongsa et al. [35] (health literacy or information gap; primary care provider-reported barriers to care, including limited English proficiency), and Kizawa et al. [38] (health literacy or information gap; knowledge of and readiness for advance care planning). The gap was reduced, with a statistically significant between-group improvement in the direct measure, in two of these reports [23,24]; it was partially reduced in one [35], in which the improvement in barriers to care was provider-perceived and limited to a comparison between two active interventions (automated telephone self-management support versus group medical visits), without a usual-care comparison; and it was unchanged in one [38], in which both groups improved and no significant between-group differences were found. In the remaining 13 primary-study reports, the gap itself was not measured, and improvements in related outcomes were treated as indirect evidence only; these reports, including those of Wang et al. [30], Riegel et al. [36], and Rechenberg et al. [27], are therefore classified as “Not directly measured” in Supplementary Table S3, irrespective of the direction of effect on their primary outcomes.

### 3.3. Evidence from Systematic Reviews

Three systematic reviews with meta-analysis were included and synthesized separately from the primary studies (Table 2b). Two reviews evaluated nurse-led transitional care or early discharge planning and targeted continuity of care, and one evaluated nurse-managed protocols in outpatient care and targeted quality of care. All three were rated excellent (91%). Le Berre et al. [42] reported reductions in mortality, readmissions, and readmission days, although quality of life did not consistently improve. Zhu et al. [40] reported reduced readmissions, readmission length of stay, and mortality. Shaw et al. [41] reported modest improvements in HbA1c, blood pressure, and cholesterol. Because each review reported significant effects on some but not all outcomes, all three were classified as showing mixed effects.

Of the 16 primary studies, 2 were also included in at least one of the systematic reviews [34,36] (Wong et al. [34] in Zhu et al. [40], and Riegel et al. [36] in Le Berre et al. [42]; Supplementary Table S6), and 14 were not included in any review. Neither study was included in more than one review. Overlap among the three systematic reviews themselves was not quantified, because their complete sets of included studies were not mapped; as two of the reviews [41,42] evaluated transitional care in overlapping populations, some trials may have contributed to both. Review-level findings were therefore not added to the primary-study counts, and agreement between the two evidence streams was not interpreted as independent corroboration where the same trials contributed to both.

### 3.4. Quality Assessment

Using the thresholds described in Section 2.5, of the 17 primary-study reports, one quasi-experimental study was rated moderate (67%), and the remaining 16 were rated low (8–54%); the three systematic reviews were rated excellent (91%) (Table 3, Table 4, Table 5 and Table 6). The studies included in this review evaluated nurse-led, coaching, and coordinated-care interventions. Considered collectively, they provide promising but methodologically limited evidence, as methodological rigor varied considerably across trials. Sequence generation was a relative strength in several studies, which used computer-generated sequences, genuine random-number methods, or other robust randomization procedures [24,29,32,33,34,38]. However, the method used to generate the random sequence was insufficiently described or not reported in other studies [26,35,36]. Allocation concealment was even less consistent. It was adequately implemented in only a minority of studies [24,29,33], whereas it was absent, unreported, or insufficiently described in many others [23,27,32,34,35,36,38].

The most pervasive methodological limitation was the combination of unblinded participants and intervention providers with subjective, self-reported outcomes [24,27,29,32,33,34,38]. This increased the potential for performance and detection bias, particularly in studies in which outcome assessors were also aware of treatment allocation [35]. Although some trials used blinded data collectors or interviewers [24,29,34], outcome-assessor blinding was judged as not achieved in Table 3 because the outcomes concerned were self-reported by unblinded participants. The risk of detection bias was lower in trials that incorporated objective clinical, healthcare-utilization, or cost outcomes [26,32,36].

Clinically relevant baseline imbalances were identified in several studies and were not always appropriately adjusted for in the analyses [24,26,32,34,36,38]. In addition, intention-to-treat analysis was frequently incomplete, absent, or inadequately reported. This concern was often compounded by substantial attrition, participant exclusions, or insufficient handling of missing data [23,26,27,29,32,33,34,35,36].

Among the cluster-randomized trials, a recurring and potentially serious limitation was the failure to account adequately for clustering in the sample-size calculation or statistical analysis [23,24,33,36]. In some cases, this issue was further aggravated by the inclusion of very few clusters [23,33]. Conversely, Leff et al. [26] reported analytical approaches intended to account for clustering, although the adequacy of these could not be fully judged from the report.

Additional concerns included reliance on within-group rather than between-group comparisons [27], multiple statistical comparisons without adjustment for multiplicity [34,38], inconsistencies in reported data or participant flow [32,33], and the preliminary or pilot nature of several studies [23,26,27]. Overall, the reviewed interventions show potential; however, the findings should be interpreted cautiously because of the substantial variability in study quality and the recurring risks of selection, performance, detection, attrition, and statistical-analysis bias. The detailed critical-appraisal judgments for each included study are presented in Table 3.

Critical appraisal is presented in the Table 4.

Overall, the five studies suggest that nurse-led interventions may improve care for people with chronic or advanced conditions, but confidence in these findings is limited because most studies had important design limitations. Schenker et al. [25] showed that the CONNECT intervention was feasible and acceptable, but the single-arm pilot design could not establish clinical effectiveness. Mallow et al. [31] reported improvements in glucose and blood pressure after a technology-supported intervention, but the absence of a control group means these changes cannot be confidently attributed to the intervention.

The controlled studies provided somewhat stronger evidence. Schraeder et al. [28] was the most methodologically robust because it included a large comparison group, objective healthcare-use outcomes, and statistical adjustment, although non-random geographical allocation and baseline differences still created a risk of confounding. Akkuş and Akdemir [37] found a possible improvement in physical quality of life, but the small sample, predictable allocation, attrition, and lack of blinded assessment reduced confidence in the result. Nualchaem et al. [39] reported improvements in self-management, function, glucose and blood pressure, but site-based allocation, participant loss and inconsistencies in the reported tables and statistical results indicate a high risk of bias.

Therefore, all studies can be included in a narrative synthesis, but their findings should be interpreted cautiously. The combined evidence supports the feasibility and potential benefit of nurse-led care, rather than providing firm proof of effectiveness. Overall, the risk of bias in these studies is moderate to high.

The three systematic reviews were synthesized separately from the primary studies. All three reviews were appraised as having excellent methodological quality (10/11; 91%; Table 6). Zhu et al. [40] and Le Berre et al. [42] reported reductions in readmissions and mortality associated with transitional care interventions, while Shaw et al. [41] reported modest improvements in HbA1c, blood pressure, and cholesterol associated with nurse-managed care.

## 4. Discussion

This systematic review examined which nursing interventions address care gaps in chronic disease management and the outcomes associated with implementing these interventions among adults with chronic diseases.

A prominent finding of this review was the frequent focus on continuity of care, which emerged as the most addressed care gap across the included studies. In the primary studies, continuity-focused interventions included telephone follow-up and case-management models [34,36] and more recent multicomponent and digitally supported approaches [30,32,33]; at the review level, two systematic reviews of nurse-led transitional care and discharge planning [40,42] reported reductions in readmissions and mortality. Although findings varied, the primary studies generally reported improvements in self-management, medication adherence, psychological well-being, self-efficacy, and patient satisfaction. In contrast, evidence from the systematic reviews was synthesized separately and suggested reductions in readmissions, mortality, and hospital utilization, particularly in transitional care interventions. Together, these findings suggest that continuity-focused nursing interventions are associated with improvements in both care processes and patient outcomes, particularly during transitions between hospital and community care. However, continuity of care itself was not measured directly in any of the included primary studies, so these findings represent indirect evidence (Supplementary Table S3). This interpretation is supported by recent evidence emphasizing post-discharge follow-up, monitoring, patient education, and sustained nursing support as key elements of effective continuity strategies [43,44]. Similar findings were reported by Markovich et al. [45], who conceptualized continuity of care as a multidimensional construct comprising informational, management, and relational continuity. Their review highlighted ineffective information transfer and unclear post-discharge responsibility as major contributors to fragmented care transitions, particularly among older adults and individuals with complex chronic conditions. These observations are consistent with the present review, in which fragmented care emerged as an important care gap, particularly among individuals with multimorbidity. Fragmented care is associated with poorer outcomes among patients with chronic illnesses [6]. Nurse-led case-management interventions [26,28,39] were associated with improvements in outcomes related to care coordination, service integration, self-management, and hospital utilization, although fragmented care itself was not measured directly and the between-group effect in Leff et al. [26] was not statistically significant. This is further supported by evidence indicating that integrated care models can improve quality of life, treatment adherence, access to health and social services, and patient experiences among individuals with complex chronic conditions, highlighting the importance of coordinated approaches in reducing care fragmentation [10,46,47].

Although quality-of-care deficits were less frequently addressed, positive outcomes were still observed. In a primary study, Boult et al. [24] reported improvements in patient-perceived quality of care with a nurse-led case-management model, and at review level, Shaw et al. [41] found that nurse-managed protocols were associated with modest improvements in clinical risk factors (HbA1c, blood pressure, and cholesterol). These findings suggest that an expanded nursing role may support more proactive, guideline-concordant chronic disease management. Similar conclusions have been reported in recent reviews, which identified nurse-led interventions as effective strategies for improving chronic disease outcomes through patient education, medication management, self-management support, and coordinated care delivery [48,49].

Care deficits were primarily addressed through self-management support interventions. Meekaew et al. [23] reported improvements in disease-related knowledge, self-care behaviors, blood pressure control, microalbuminuria, and glycemic outcomes, underscoring the potential of nurse-led educational and behavioral support to strengthen patients’ self-management capacity. Similar benefits have been reported in recent reviews, which show that nurse-led self-management interventions can improve disease knowledge, self-care skills, medication adherence, and clinical outcomes among people living with chronic conditions [12,49]. The reported benefits of these interventions may be explained by their emphasis on patient education, motivational approaches, individualized assessment of care needs, and ongoing nurse-patient relationships, which have been identified as important mechanisms supporting chronic disease self-management [50].

Access-to-care gaps were primarily addressed through telehealth and digital health interventions. Mallow et al. [31] reported pre–post improvements in clinical outcomes among underserved rural patients with multimorbidity, although the absence of a control group precludes attribution to the intervention, and Fu et al. [33] reported mixed effects on self-management and quality of life with a nurse-led digital continuity-of-care platform compared with an active comparator (standardised nurse telephone follow-up); in neither study was the targeted gap measured directly. Recent evidence indicates that nurse-led digital interventions can improve self-management, clinical outcomes, healthcare utilization, and patient satisfaction among individuals living with chronic conditions [44]. Nurse-led telehealth interventions have also been recognized as an important strategy for overcoming geographical, temporal, and organizational barriers to healthcare, thereby improving access and promoting more equitable delivery of chronic disease services, particularly for underserved populations [51].

Health literacy and information-related gaps were addressed through self-management education, health coaching, telehealth support, and counseling interventions [27,35,38]. These interventions were linked to improvements in disease-related knowledge, patient activation, self-management behaviors, treatment adherence, and participation in healthcare decision-making. However, comparative evidence for this gap was limited: Ratanawongsa et al. [35] showed only provider-perceived reductions in barriers to care, limited to a comparison between two active interventions; Kizawa et al. [38] found no significant between-group differences; and the improvements reported by Rechenberg et al. [27] were within-group changes without between-group testing. Recent evidence also shows that self-management interventions can improve self-efficacy, quality of life, and psychological outcomes among people living with chronic diseases [52]. Nurses play a central role in strengthening health literacy and supporting informed decision-making, helping reduce information-related care gaps and promote patient empowerment [53].

Unmet patient needs were primarily addressed through psychosocial support, counseling, home visits, and individualized follow-up interventions [25,29,37]. Reported benefits included improvements in psychological well-being, quality of life, and patient satisfaction, particularly among individuals with advanced or complex chronic conditions. These findings underscore the value of person-centered nursing approaches that address emotional, psychosocial, and supportive care needs alongside disease management. Recent evidence similarly highlights the role of nurse-led supportive interventions in improving psychosocial outcomes and quality of life among individuals living with chronic illnesses [10].

Across the included studies, nurse-led case management, telehealth follow-up, and self-management support were the most commonly implemented interventions. Despite variation in study settings, populations, and intervention components, these approaches were frequently associated with improvements in outcomes related to care coordination, continuity of care, self-management capacity, health literacy, healthcare access, and selected clinical outcomes. This suggests that multifaceted, patient-centered nursing interventions may help address a broad range of care gaps among adults with chronic diseases; however, because the targeted gaps were rarely measured directly, these associations should not be interpreted as evidence that the gaps themselves narrowed. A study-level synthesis of key findings, barriers and facilitators, author-reported limitations, and preliminary themes is provided in Supplementary Table S4.

This review has several limitations. The pronounced heterogeneity of the included studies precluded meta-analysis and required a narrative synthesis, so the findings should be read as a qualitative overview rather than pooled estimates of effect. Eligibility was restricted to English-language, full-text publications with no search of grey literature, so language and publication bias cannot be excluded. In addition, the search relied on free-text terms without controlled vocabulary (e.g., MeSH or CINAHL Subject Headings), which may have reduced its sensitivity. Although primary studies and systematic reviews were synthesized separately, 2 primary studies were also included in at least one review (Supplementary Table S6), so the two evidence streams are not fully independent; moreover, overlap among the three systematic reviews themselves was not quantified. The methodological quality of the included studies was variable and generally limited, and no formal framework (e.g., GRADE) was used to rate outcome-level certainty of evidence; the reported benefits should therefore be interpreted as associations rather than definitive evidence of effectiveness. Another limitation concerns the PROSPERO registration, which occurred after the literature search had been completed, and the deviations from the registered methods described in Section 2.6. Although study selection, data extraction, quality appraisal, and data synthesis had not started at the time of registration, retrospective registration and these deviations reduce the transparency of protocol development. Risk of bias due to missing results was also not formally assessed. In addition, most included studies did not measure the targeted care gap directly, and the available direct evidence was limited: in one of the four reports with a direct measure, the improvement was provider-reported and based on a comparison between two active interventions, and in another, no between-group difference was found against an active comparator. Classification of gap change therefore relied largely on related outcomes and involved reviewer judgment. Finally, the evidence was concentrated in a few countries, most notably the USA and China, which limits the transferability of the findings to other settings and health systems.

Future research should further explore less frequently investigated care gaps, particularly quality-of-care deficits, care deficits, and access-to-care barriers, and should measure the targeted care gaps directly using validated indicators alongside patient and clinical outcomes.

## 5. Conclusions

Nurse-led interventions frequently target multiple care gaps in chronic disease management and were associated with improvements in selected patient, service, and clinical outcomes. Because the targeted gaps were rarely measured directly, these findings indicate potential rather than demonstrated reductions in care gaps. These findings underscore the important role of nurses in supporting integrated, coordinated, and patient-centered chronic care.

## Figures and Tables

**Table 1:** Inclusion and exclusion criteria.

Criterion	Inclusion	Exclusion
Population	Adults (≥18 years) with one or more chronic diseases	Pediatric populations; conditions not classified as chronic diseases
Intervention	Nursing care, nursing interventions, or nursing management strategies	Interventions without a nursing component; exclusively medical or pharmacological treatment
Focus and outcomes	Addresses care gaps, unmet needs, or deficiencies in chronic disease management and reports patient- or care-related outcomes (e.g., quality of life, self-management, patient satisfaction, continuity of care, access to care, or quality of care)	Does not address care gaps or does not report patient- or care-related outcomes
Study design	Quantitative, qualitative, mixed-methods, and intervention studies; systematic reviews or meta-analyses synthesizing evidence on nursing interventions	Editorials, opinion papers, commentaries, conference abstracts, protocols, narrative reviews, and scoping reviews
Publication	Full-text, peer-reviewed articles published in English	Non-full-text articles; theses, dissertations, and other non-peer-reviewed reports; studies not published in English

## Data Availability

No new data were created or analyzed in this study. Data sharing is not applicable to this article as all analyzed sources and findings are included within the manuscript.

## Supplementary Materials

The following supporting information can be downloaded at: https://www.mdpi.com/article/10.3390/healthcare14203534/s1, Table S1. Extended data-extraction matrix (Part 1 of 4): study identification, context, and population characteristics of the 20 included reports (17 reports of 16 primary studies and three systematic reviews). Supplementary material: Table S2. Extended data-extraction matrix (Part 2 of 4): characteristics of the nursing intervention. Supplementary material: Table S3. Extended data-extraction matrix (Part 3 of 4): targeted care gaps and reported outcomes. Supplementary material Table S4. Extended data-extraction matrix (Part 4 of 4): synthesis—key findings, barriers and facilitators, limitations, and preliminary themes. Supplementary Table S5. Preferred Reporting Items for Systematic Reviews and Meta-Analyses (PRISMA 2020) Checklist. Supplementary Table S6. Citation matrix of overlap between included systematic reviews and primary studies. Supplementary Table S7. Complete database-specific search strategies as executed, including platforms, searched fields, applied limits, and records retrieved.

## Abbreviations

The following abbreviations are used in this manuscript:

Abbreviation	Full Term
CAPD	Continuous ambulatory peritoneal dialysis
CHD	Coronary heart disease
CHF	Chronic heart failure
CINAHL	Cumulative Index to Nursing and Allied Health Literature
CKD	Chronic kidney disease
CONNECT	Care Management by Oncology Nurses
CVD	Cardiovascular disease
ESRF	End-stage renal failure
GenAI	Generative artificial intelligence
HbA1c	Glycated hemoglobin (hemoglobin A1c)
HF	Heart failure
JBI	Joanna Briggs Institute
MA	Meta-analysis
PIO	Population, Intervention, Outcome
PRISMA	Preferred Reporting Items for Systematic Reviews and Meta-Analyses
PROSPERO	International Prospective Register of Systematic Reviews
RCT	Randomized controlled trial
SR	Systematic review
T2DM	Type 2 diabetes mellitus
USA	United States of America
