# Supplementary material for: Addressing Care Gaps in Chronic Disease Management: A Systematic Review of Nursing Interventions
Source: Healthcare (Basel). 2026 Oct 20;14(20):3534. doi: 10.3390/healthcare14203534 (PMC13704512; doi:10.3390/healthcare14203534)
Supplement: Supplementary file 1 [file healthcare-14-03534-s001.zip › healthcare-4583723-supplementary.pdf]

Supplementary material: Table S1. Extended data-extraction matrix (Part 1 of 4): study identification, context, and population characteristics of the 20 included reports (17 reports of 16 primary studies and three systematic reviews).

ID	Author, year	Country	Setting	Study design	Aim	Chronic condition(s)	Sample size (N int./control)	Age, sex, and other characteristics
1	Meekaew et al. [23]	Thailand	Outpatient clinic	Cluster RCT	To evaluate the effectiveness of a nurse-led intervention delivered via the LINE Official Account (OA) smartphone application on knowledge, self-care and clinical outcomes in adults with type 2 diabetes and comorbidities.	Type 2 diabetes with comorbid hypertension and CKD stage 3 (A1C >= 7.5%, BP >= 140/90 mmHg, eGFR 30-59 ml/min/1.73 m2, microalbuminuria >= 30 mg/L).	120 randomised (60 intervention / 60 control); 108 analysed (55 / 53). Lost to follow-up: 5 intervention, 7 control. 457 screened for eligibility.	Mean age 57.7 (SD 9.7) intervention vs 59.2 (SD 9.5) control; female 68.3% vs 66.7%; majority married with primary school education; mean duration of diabetes 9.2 vs 11.0 years; no significant baseline differences (P > .05). Sampling method: cluster randomisation was used because each general hospital has only one NCD clinic, so the design guards against contamination - 2 of 4 general hospitals in Ratchaburi province were randomly selected and randomly assigned, one clinic to the intervention group and one to the control group; within the clinics the researcher recruited patients meeting the inclusion criteria (adults aged 20 or older, A1C >= 7.5%, BP >= 140/90 mmHg, eGFR 30-59 ml/min/1.73 m2, MAU >= 30 mg/L, on oral medication, with a smartphone running the LINE OA application and able to communicate in Thai). 457 patients were screened for eligibility (239 at the intervention clinic, 218 at the control clinic) and 120 were enrolled. The sample size was calculated with G*Power on A1C as the primary outcome (power 0.80, alpha 0.05, effect size 0.56 - 51 per group), inflated to 60 per group for an anticipated 20% attrition. Attrition: 5 of 60 lost in the intervention group (1 moved to another area, 4 unable to continue) and 7 of 60 in the control group (2 moved away, 5 unable to continue), leaving 55 and 53 analysed (8.3% and 11.7% loss).
2	Zhu et al. [40]	China	Hospital / acute care	Systematic review with meta-analysis	To compare the effectiveness of nurse-led early discharge planning programmes with standard care for hospital inpatients with chronic disease or rehabilitation needs, using length of stay, hospital readmission, readmission length of stay and all-cause mortality as principal outcomes.	Mixed chronic conditions: heart failure, coronary heart disease, chronic disease in hospitalised older adults, hip fracture rehabilitation and hospitalised psychiatric patients. Acute and critical illness excluded.	10 RCTs, 3438 participants (1709 received discharge planning in addition to standard care / 1729 standard care only). Individual trial samples ranged from 30 to 749 patients. 468 citations screened.	Predominantly older adults; 8 trials recruited patients aged 65 and over, 2 recruited patients under 65. Trial mean ages ranged from about 36 to 79 years; proportion of men ranged from about 25% to 64%. Sampling method (study selection): systematic search of PubMed (1966 to March 2014), MEDLINE, EMBASE, CINAHL, the Cochrane Library and reference lists for RCTs with parallel controls comparing nurse-led early discharge planning with standard care in inpatients with chronic disease or rehabilitation needs; last search 29 March 2014; non-English publications excluded. One author screened titles, two authors independently screened abstracts using a full-text screening form, and disagreements were resolved by discussion or a third author. Attrition (study-selection flow): 468 records identified and screened, 433 excluded (41 non-human, 35 non-English, 356 non-RCT), 35 full texts assessed, 24 excluded for not meeting the criteria, 11 eligible, 1 duplicate removed, leaving 10 RCTs (3438 participants) in the meta-analysis; participant dropout within the individual pooled trials was not extracted at review level.
3	Shaw et al. [41]	USA	Outpatient clinic	Systematic review with meta-analysis	To determine whether nurse-managed protocols are effective for the outpatient management of adults with diabetes, hypertension and hyperlipidaemia.	Type 1 and type 2 diabetes, hypertension and hyperlipidaemia in adults with elevated cardiovascular risk.	18 unique studies (16 RCTs, 2 controlled before-and-after studies), 23 004 patients, from 2954 screened citations.	Median age 58.3 years (range 37.2-72.1) across 16 studies; approximately 47% female; race not reported in 84% of studies. Patients with diabetes had baseline A1C around 8.0% or higher, patients with hypertension had moderate hypertension and those with hyperlipidaemia had borderline high lipid levels. Sampling method (study selection): searched MEDLINE (via PubMed), the Cochrane Central Register of Controlled Trials, EMBASE and CINAHL from 1 January 1980 to 31 January 2014 for English-language, peer-reviewed RCTs and quasi-experimental studies comparing nurse-managed protocols with usual care in adults with chronic conditions, plus bibliography searches and a ClinicalTrials.gov search for unpublished studies. Two reviewers applied prespecified eligibility criteria to all titles, abstracts and full texts, resolving disagreements by discussion or a third reviewer; one reviewer abstracted data and assessed quality, confirmed by a second. Attrition (study-selection flow): 2954 unique citations identified, 2615 excluded at title/abstract, 339 retrieved for full-text review, 319 excluded (not English/westernized country/full publication 55; not adults with the disease of interest or not outpatient 29; ineligible design

ID	Author, year	Country	Setting	Study design	Aim	Chronic condition(s)	Sample size (N int./control)	Age, sex, and other characteristics
								or comparator 75; no intervention of interest 153; no outcome of interest 7); of 23 potentially eligible studies 4 were excluded because the nurses' authority to titrate could not be verified and 1 because the target illness could not be distinguished, leaving 18 unique studies (23 004 patients, plus 2 companion articles); participant attrition within the pooled trials was not extracted at review level.
4	Le Berre et al. [42]	Canada	Transitional (hospital-to-home)	Systematic review with meta-analysis	To determine the impact of transitional care interventions for older patients with chronic disease transitioning from hospital to home on mortality, service use (emergency department visits, hospital readmission, readmission days) and quality of life.	Chronic heart failure (66% of the pooled population), respiratory conditions (9%), cancer (2%) and 22% with other conditions including multimorbidity, a geriatric profile, frailty or polymedication. Alcohol dependence, spinal cord injury, myocardial infarction, hip fracture, depression and stroke were excluded.	92 RCTs included from 10 234 screened references. Pooled analyses: 68 trials for mortality, 20 for emergency department visits, 76 for readmission, 41 for readmission days and 18 for quality of life.	Patients aged 65 and over with at least one chronic disease, hospitalised and discharged home. Mean age across intervention groups 73.8 (SD 4.8) years; 47% female. Sampling method (study selection): a specialised librarian searched MEDLINE, CINAHL, PsycINFO and EMBASE with geriatric, transition, hospital, primary-care and chronic-disease terms for RCTs published in English or French between 1 January 1995 and 9 April 2015; reference lists of included studies and companion papers were also screened. Two reviewers independently examined titles and abstracts and then full texts, resolving any disagreement by consensus. Attrition (study-selection flow): 10 234 references identified, 9574 excluded on title or abstract and 568 on full text, leaving 92 RCTs; participant dropout within the individual pooled trials was not extracted at review level, although it informed the sensitivity analyses that removed studies at risk of bias.
5	Boult et al. [24]	USA	Primary care	Cluster RCT	To test the hypothesis that Guided Care improves the quality of health care for multimorbid older patients and physicians' satisfaction with chronic care within six months.	Multimorbidity in patients aged 65 and over in the upper quartile of risk of heavy health service use (hierarchical condition category score >= 1.2); mean 4.3 self-reported chronic conditions (range 0-13).	13 534 patients screened, 3692 at high risk, 2391 eligible, 904 consented and cluster-randomised (485 Guided Care / 419 usual care); 767 analysed at six months (408 / 359). 14 pods, 49 physicians, 8 practices, 7 nurses.	Mean age 77.2 (range 66-106) Guided Care vs 78.1 (66-96) usual care; 54.2% vs 55.4% female; about 51% and 49% Caucasian and 46% African American; approximately 31% living alone; mean SF-36 Physical Component 38.7 vs 38.1 and Mental Component 50.3 vs 48.7. Sampling method: multistage recruitment within a cluster-randomised design. Patients of participating physicians were eligible for screening if aged 65 or older and covered by fee-for-service Medicare Parts A and B, Kaiser-Permanente Medicare Advantage or TriCare; their previous 12 months of insurance claims were analysed with the hierarchical condition category (HCC) model, and those with an HCC score of 1.2 or higher (top 25% of predicted use) entered recruitment. High-risk patients received an introductory opt-out letter, then a telephone call and the offer of an in-home meeting; professional interviewers visited consenting patients' homes to screen, describe the study, obtain written consent and complete the baseline interview (ineligible if without a telephone, non-English-speaking, planning extended travel, or failing a cognitive screen without a proxy). The unit of randomisation was the 'pod' (a colocated team of 2-5 primary care physicians and their consenting patients); the study statistician randomly assigned the 14 pods to Guided Care or usual care. 13 534 patients were screened, 3692 (27.3%) were at high risk, 2391 were alive, accessible and eligible, and 904 (37.8%) consented and were cluster-randomised (485 Guided Care / 419 usual care). Attrition: at 6 months, 77 of 485 Guided Care participants were not analysed (55 lost to follow-up - 28 deaths, 13 dropouts, 9 unable to contact, 5 declined the interview - plus 22 who changed physicians) and 60 of 419 usual-care participants were not analysed (50 lost - 24 deaths, 4 dropouts, 17 unable to contact, 5 declined - plus 10 who changed physicians), leaving 408 and 359 analysed; follow-up rates among those still alive and with the same physician were 93.8% and 93.2%.
6	Schenker et al. [25]	USA	Outpatient clinic	Quasi-experimental	To assess the feasibility, acceptability and perceived effectiveness of an oncology nurse-led care management approach (CONNECT) for improving the provision of primary	Advanced cancer - metastatic solid tumours: lung 26%, colon 26%, prostate 13%, pancreatic 9%, oesophageal 9%, breast, cervical, ovarian and neuroendocrine tumour 4% each. Haematological malignancies excluded.	23 patients, 19 caregivers and 5 oncologists; single-arm pilot with NO control group. 48 potential participants identified, 35 approached, 30 consented, 23 enrolled.	Patients mean age 67 (SD 14), 48% male, 96% Caucasian; ECOG performance status 0 in 39%, 1 in 57%, 2 in 4%; 87% receiving chemotherapy. Caregivers mean age 57 (SD 17), 37% male, 58% spouse or partner, 63% living with the patient. Sampling method: consecutive clinic-based (convenience) sampling in a single-arm pilot with no randomisation. Oncology clinic staff identified adult patients with upcoming appointments who had metastatic solid tumours and for whom the oncologist 'would not be surprised' if the patient died or was admitted to the ICU within a year, with ECOG performance status 0-2, planning ongoing care with a participating oncologist and willing to be seen at least

ID	Author, year	Country	Setting	Study design	Aim	Chronic condition(s)	Sample size (N int./control)	Age, sex, and other characteristics
					palliative care in oncology clinics.			monthly; patients with haematological malignancies, unable to read and respond in English, unable to complete the baseline interview or lacking decision-making capacity were excluded, and consenting patients identified a caregiver. Enrolment ran January to April 2014. Attrition: 48 potential participants were identified, 35 approached (13 not approached - 8 per the oncologist, 3 did not show or cancelled, 2 ineligible), 30 consented (5 not - 4 declined, 1 deceased) and 23 enrolled (7 not enrolled - 4 withdrew, 3 ineligible); consent-to-approach rate 86%, enrolled-to-consent rate 77%. No participant withdrew after enrolment; 4 patients died during the study (at 10, 60, 66 and 82 days), and three-month outcome assessments were completed with all 19 remaining patients and 15 caregivers (83% of participants), with about 1% missing data.
7	Leff et al. [26]	USA	Primary care	Cluster RCT	To evaluate the preliminary effects of Guided Care on health service utilisation and costs during the first 8 months of an ongoing cluster-randomised controlled trial, in order to inform business and policy decisions before publication of the final results in 2011.	Multimorbidity in patients aged 65 and over at high risk of heavy health service use (hierarchical condition category score >= 1.2, upper quartile of risk). Individual chronic diagnoses are not listed in this paper.	13,534 patients screened, 1763 offered participation, 904 consented and cluster-randomised (485 Guided Care / 419 usual care); 835 analysed (433 / 402). 69 participants (7.6%) excluded after randomisation: insurance claims unavailable 1.4%, consent rescinded 1.8%, died before start date 2.2%, no start date before 30 June 2007 2.2%. 8 practices, 14 pods, 49 physicians.	Aged 65 and over; mean age and sex distribution are not reported in this paper (reported in Boult et al. 2008, ID 5). Insurance: 34% fee-for-service Medicare, 45% Kaiser Permanente, 21% Tricare/USFHP. Baseline differences favouring Guided Care: lacked enough money at the end of the month 10.4% vs 15.5% ($p = 0.05$); difficulty with 2 or more instrumental activities of daily living 21.7% vs 28.3% ($p = 0.03$); excellent or very good self-rated health 23.1% vs 17.3% ($p < 0.001$); insured by Tricare/USFHP 26.8% vs 16.5% ($p = 0.001$). Demographics, comorbidity and risk scores otherwise similar. Mean observation period 8.3 vs 7.8 months ($p < 0.05$); 83% observed for at least 6 months. Sampling method: whole-panel claims screening rather than volunteer sampling - the insurance claims of all patients of the 8 practices were screened (13,534 people) to identify those aged 65 and over with an HCC score of 1.2 or higher, the highest quartile of predicted use; 1763 eligible high-risk patients were offered participation and 904 (51.3% of those offered) consented and completed an in-home baseline interview. Allocation was at cluster level: the study statistician, blinded to the identity of the pods, randomly assigned the 14 pods to Guided Care or usual care. Attrition: no follow-up losses are reported within the 8-month analysis window, but 69 of the 904 randomised participants (7.6%) were excluded before analysis - insurance claims unavailable 1.4%, consent rescinded 1.8%, died before their start date 2.2%, no start date before 30 June 2007 2.2%; these losses are not broken down by group.
8	Rechenberg et al. [27]	USA (federally qualified health centers; two study sites, not named in the paper)	Primary care	RCT	To test the feasibility and acceptability of a language concordant health coaching intervention (IL2L for Chronic Care) delivered by nurse and nurse practitioner students in a pilot study of Latinx immigrants with type 2 diabetes and limited English proficiency, and to present preliminary trends in glycaemic control (A1c), anxiety, depressive symptoms and patient satisfaction.	Type 2 diabetes with out-of-range glycaemic control (current A1c > 7) in Latinx immigrants with limited English proficiency (LEP). Anxiety and depressive symptoms were measured as psychosocial comorbidity. Patients with symptoms of long-term diabetes complications (retinopathy, neuropathy, nephropathy) or severe depression were excluded.	22 potential participants identified by the clinic; 18 enrolled (82% response rate, reported as 81% in the discussion); 1 lost to the study (6%); 17 analysed (10 intervention / 7 control). Psychosocial analyses were run on 9 intervention participants after deletion of one large outlier. The power analysis required 19 participants ($dz = 0.6$, one-tailed dependent t-tests, $\alpha = 0.05$) and 22 (11 per arm) were to be recruited.	Mean age 51.7 (SD 12.9) years; 64.7% female; all Latinx immigrants, primarily Spanish-speaking with LEP, aged 22 or older. Mean A1c 10.9 (SD 2.0); mean weight 185.46 lbs (SD 40.5); mean BMI 32.98 (SD 6.92). 47.1% partnered, 76.5% employed, 11 participants (65%) with annual family income of $20,000 or less. No statistically significant differences between groups in demographic or general health characteristics. Group-specific age and sex distributions are not reported. Sampling method: clinic-based convenience recruitment (the authors do not use the term convenience sampling) from federally qualified health centers - the clinic identified patients carrying a diagnosis of T2D with A1c above 7, Spanish-language brochures were distributed in waiting rooms, primary care providers introduced the study to eligible patients, and interested patients were referred to a trained on-site bilingual promotor; a simple randomisation sheet prepared in advance by the statistician determined the group of each successive participant. Attrition: 22 potential participants identified, 18 enrolled (82% response rate, reported as 81% in the discussion), 1 lost to the study (6%) and 17 analysed; the paper does not state which group lost the participant. One large outlier was additionally excluded from the depression analysis, leaving n = 9 for the psychosocial outcomes.
9	Schraeder et al. [28]	USA (central Illinois:	Primary care	Quasi-experimental	To test the effectiveness of a collaborative primary care nurse case	Multimorbidity in community-dwelling adults aged 65 and over screened	4053 new health plan members were mailed a 50-item health questionnaire; 3562 were	Mean age 75.4 (SD 7.1) treatment vs 76.4 (SD 7.9) comparison ($p = 0.067$); female 53.4% vs 60.3% ($p = 0.084$). Significant baseline differences: minority race 7.3% vs 23.1% ($p < 0.001$); less than high school education 26.3% vs 34.7% ($p = 0.021$);

ID	Author, year	Country	Setting	Study design	Aim	Chronic condition(s)	Sample size (N int./control)	Age, sex, and other characteristics
		treatment site 8 counties in east central Illinois, primarily rural; comparison site 13 counties in west central Illinois with a more diverse population)			management intervention emphasising collaboration between physicians, nurses and patients, risk identification, comprehensive assessment, collaborative planning, health monitoring, patient education and transitional care, on healthcare utilisation and cost for community-dwelling chronically ill older people. The hypotheses were that the intervention would reduce service utilisation, lower total costs of care and prove revenue neutral.	as high risk for mortality, functional decline or increased health service use. Baseline conditions, treatment vs comparison: heart disease (congestive heart failure and/or angina) 42.3% vs 41.9%; diabetes 29.0% vs 31.4%; myocardial infarction 23.6% vs 21.7%; COPD 16.5% vs 15.9%; urinary incontinence 14.3% vs 13.7%; stroke 13.5% vs 12.6%; depression 12.5% vs 11.2%; cancer 9.8% vs 14.8% (p = 0.045). 45.3% vs 43.3% took 5 or more prescription medications daily.	returned (88% response rate; treatment site 2102, comparison site 1460). 677 were screened high risk and formed the study sample: treatment group n = 400, comparison group n = 277 (19% of the assessed population). The 2884 members not screened as high risk (treatment site 1701, comparison site 1183) were analysed separately as a check on design effects. No attrition figures for the analysed sample are given, but voluntary disenrolment from the health plan was 8% in the treatment group and 22% in the comparison group (p < 0.001).	lives alone 26.8% vs 33.9% (p = 0.049); cancer 9.8% vs 14.8% (p = 0.045); complete physical in the past 12 months 67.2% vs 53.4% (p < 0.001); any specialist visit in the previous 6 months 53.3% vs 61.0% (p = 0.049). Similar between groups: 2 or more ADL limitations 23.1% vs 23.3%; 2 or more IADL limitations 16.8% vs 17.3%; fair or poor self-rated health 44.4% vs 46.9%; possible cognitive impairment 5.8% in both. Treatment patients were enrolled about 3 months longer (27.6 months, SD 7.9, vs 24.8, SD 8.6; p < 0.001). All patients were community dwelling (not in a nursing home at enrolment) and had the same covered health benefits. Sampling method: population screening of a health plan rather than volunteer sampling - all 4053 people who joined the plan during its first year of operation were mailed a 50-item health questionnaire, with telephone contact for non-responders, and 3562 (88%) responded (treatment site 2102, comparison site 1460); those aged 65 and over, not resident in a nursing home at enrolment, and screened as high risk on 24 selected questions formed the study sample of 677, i.e. 19% of the assessed population. Allocation was geographical rather than random: the eight-county east central Illinois site received the intervention and the 13-county west central Illinois site served as comparison. Attrition: no participant flow diagram or loss-to-follow-up figures are given for the analysed sample, and analysis was by intention to treat on all 677; however, voluntary disenrolment from the health plan differed markedly between sites (8% treatment vs 22% comparison, p < 0.001) and treatment patients were followed about three months longer (27.6 vs 24.8 months, p < 0.001), so exposure and dropout were unequal.
10	Xiao et al. [29]	China (mainland; respiratory medicine ward of Hunan Cancer Hospital, Changsha; research team based in Changsha and Hong Kong)	Hospital / acute care	RCT	To examine the effectiveness of a culturally adapted, family-oriented dignity therapy in improving dignity-related distress (primary aim) and, as secondary aims, depression and spiritual well-being, and to test whether the effects were sustained four weeks after the intervention, in Chinese patients with lung cancer undergoing chemotherapy.	Lung cancer undergoing chemotherapy: non-small-cell 76.7%, small cell 23.3%; 92.5% at an advanced stage (stage III 30.8%, stage IV 57.5%, extensive stage 4.2%). Dignity-related distress, depressive symptoms and spiritual well-being were the target problems. Patients and family caregivers with cognitive impairment or psychiatric illness were excluded.	482 dyads screened; 311 did not meet the inclusion criteria; 51 of the 171 eligible dyads declined; 120 dyads (patient plus one significant family caregiver, 240 participants) were randomised, 60 to family-oriented dignity therapy and 60 to attention control. All completed baseline. Analysed at T1 (one week): 94 dyads (45 intervention / 49 control); at T2 (four weeks): 67 dyads (36 / 31). Attrition 21.7% at T1 and 44.2% at T2; 9 dyads did not complete the assigned sessions. The sample size calculation required 45 dyads per arm (80% power, 0.6 standardised mean difference, two-sided 5%), inflated to 120 for an anticipated 25% attrition. Only patient outcomes are reported in this paper.	Patients: mean age 56.03 years (SD 8.79, range 32-73); 67.5% male; 98.3% married; 75.5% had junior high school education or above; 60.0% cared for by their spouse; most had a monthly income below 1000 RMB (about 140 USD); 45.0% were newly diagnosed and had been on chemotherapy for less than two months; insurance was mainly New Rural Cooperative Medical Insurance (58.3% in both groups). By group, mean age 54.97 (SD 8.86) intervention vs 57.10 (SD 8.66) control; male 61.7% vs 73.3%. No statistically significant differences between groups in patient or family caregiver characteristics or in baseline outcome scores. Family caregiver characteristics are in the supplementary file only. Sampling method: convenience sampling of patient-family caregiver dyads from the respiratory medicine ward of a single cancer hospital; 482 dyads were assessed for eligibility between March and May 2019, 311 did not meet the selection criteria and 51 of the 171 eligible dyads (29.8%) declined, mainly for lack of time or interest; the remaining 120 dyads were randomly allocated 1:1 by block randomisation with varying block sizes of 4, 6 and 8, using sequentially numbered opaque sealed envelopes opened only after baseline data collection. Attrition: 21.7% at T1, leaving 94 dyads (45 intervention, 49 control), and 44.2% at T2, leaving 67 dyads (36 intervention, 31 control); 9 dyads (4 intervention, 5 control) discontinued the assigned sessions and the remainder were lost to physical problems, referral to other hospitals or loss of contact. Baseline characteristics of those who completed and those who dropped out were compared and did not differ significantly; analysis was by intention to treat using generalised estimating equations, with complete-case and last-observation-carried-forward sensitivity analyses.
11	Wang et al. [30]	China (cardiology department, Huanggang Central	Transitional (hospital-to-home)	Retrospective cohort study (propensity score matched)	To investigate the impact of a structured continuity of care programme on self-care capability, quality of life, mental well-being, physical condition, cardiovascular complications and	Chronic heart failure (CHF) diagnosed per the American Heart Association guidelines, NYHA class II to IV, disease course longer than 6 months. Experimental group: NYHA II 21, III 13,	90 patients admitted between January and September 2023, allocated by propensity score matching to an experimental group (n = 45) and a control group (n = 45). The number of records screened before matching, and the variables	Experimental group: 23 male / 22 female; age range 51-69 years, mean 61.41 (SD 2.13); disease course 0.5-8 years, mean 3.88 (SD 0.97). Control group: 24 male / 21 female; age range 52-71 years, mean 60.37 (SD 1.86); disease course 0.5-7.5 years, mean 3.92 (SD 1.02). Inclusion was limited to ages 40-80. The text states there was no statistically significant baseline difference between the groups (P > .05), but Table 1 reports the age comparison as t = 2.4671, P = .0156, i.e. a significant difference; disease course did not differ (t = 0.1906, P = .8493). No data on education, income, living situation or comorbidity. Sampling method:

ID	Author, year	Country	Setting	Study design	Aim	Chronic condition(s)	Sample size (N int./control)	Age, sex, and other characteristics
		Hospital, Huanggang, Hubei Province; single centre)			nursing satisfaction in patients with chronic heart failure, in order to inform more personalised nursing strategies for comprehensive heart failure management.	IV 9; control group: NYHA II 22, III 14, IV 8. Depression and anxiety were assessed as comorbid psychological states. Patients with severe liver or kidney dysfunction, an acute cardiovascular event in the previous 3 months, chronic infection, active malignancy, prior cardiac surgery or other major systemic disease were excluded.	used for matching, are not reported. The authors state there was no loss to follow-up, dropout, withdrawal or death during the observation period; missing data accounted for less than 5% of the dataset and were handled by mean imputation for continuous and mode imputation for categorical variables, with a sensitivity analysis.	retrospective selection from the clinical records of patients admitted to the cardiology department between January and September 2023, with propensity score matching used to create two comparable groups of 45. The authors do not name the sampling method, and the sampling frame, the number of records screened before matching and the variables entered into the matching model are all unreported. Attrition: the authors state that there were no cases of loss to follow-up, dropout, or withdrawal due to severe complications or death during the observation period. Missing data accounted for less than 5% of the dataset and were handled by mean imputation for continuous variables and mode imputation for categorical variables, with a sensitivity analysis reported as confirming that the imputation did not materially affect the outcomes. No participant flow diagram is provided.
12	Mallow et al. [31]	USA	Community primary-care free clinic (Milan Puskar Health Right, Morgantown, West Virginia) serving uninsured/underinsured low-income rural adults aged 18-64; after enrollment, care delivered remotely to patients' homes via a web-based mHealth platform (telehealth); single clinic site; rural	Prospective single-group pre/post design (feasibility / initial-effectiveness study); described as the first trial of mI SMART, with development, feasibility and acceptability published separately	To present the initial effectiveness of mI SMART, a nurse practitioner-led web-based mHealth intervention integrating sensors and mobile devices, for improving biophysical outcomes and overcoming the access-to-care determinant in rural adults with multiple chronic conditions	Multiple chronic conditions (multimorbidity); any combination of diabetes, obesity, hypertension, depression and/or hyperlipidemia; mean 4.0 unique diagnoses; 96.7% had multiple chronic conditions	31 invited/asked to join; 30 agreed and enrolled (all but one invited agreed); feasibility sampling goal 30; no control group; biophysical paired analysis n=29 for glucose and systolic/diastolic BP and n=30 for weight/BMI; baseline surveys PHQ-9 n=28, UCLA n=27, SF-36 n=29; post-intervention survey response n=9 (too low for pre/post comparison)	Mean age 52.0 (SD 10.0; range 29-64); female 21 (70%), male 9 (30%); White 21 (70%), Hispanic 3 (10%), African American 2 (6.7%), Asian/Pacific Islander 1 (3.3%), Native American 1 (3.3%), Other 2 (6.7%); married 18 (60%); education below high school 7 (23.3%), high school/GED 10 (33.3%), some college 5 (16.7%), 2-year 3 (10%), 4-year 3 (10%), Master's 2 (6.7%); income below $20,000 56.7%; mean unique diagnoses 4.0 (SD 1.78, range 1-12); baseline UCLA loneliness 53.88 (SD 5.91, indicating loneliness), PHQ-9 7.21 (SD 6.41, mild), SF-36 total 57.33 (SD 13.58, moderate); White participants had significantly higher baseline weight/BMI than non-White. Sampling/recruitment: convenience sample of free-clinic attendees Dec 2 2014-Dec 8 2015; potential participants identified by clinic NPs then contacted by front-desk staff and invited. Attrition: no formal flow diagram; biophysical BP/glucose analyzed n=29 (1 of 30 without paired value), weight/BMI n=30; 3 refused baseline surveys; post-intervention survey response only n=9; wording '29 participated longer than the first week, remainder for at least 12 weeks' [unclear]
13	Xie et al. [32]	China	Cardiology department of a tertiary-level teaching hospital (First Affiliated Hospital of Bengbu Medical University, Anhui Province); inpatient during the hospital stay plus outpatient follow-up visits and structured weekly telephone	Prospective randomised controlled trial	To evaluate the impact of a comprehensive nursing intervention based on Orem's self-care deficit theory on self-care ability, medication adherence and mental health in older patients with chronic heart failure	Chronic heart failure (systolic HF or HF with preserved ejection fraction; NYHA II-IV) in older adults aged 60 years or older	104 older HF patients randomised via a random number table; control n=52, study n=52; no eligibility-screening, enrolled-versus-randomized or attrition numbers reported; all 104 stated to be followed for 12 months and analysed (52 per group in tables)	Age 60 or older; reported by band - control 60-70 38 (73%), 71-80 14 (27%); study 60-70 32 (62%), 71-80 20 (39%); gender control female 31 (60%), male 21 (40%); study female 23 (44%), male 29 (56%); BMI control 28.12±2.63, study 27.80±2.93; hypertension control 39 (75%), study 30 (58%); previous MI control 16 (31%), study 19 (37%); previous stroke control 24 (46%), study 21 (40%); NYHA II/III/IV control 22/26/4, study 27/19/6; no significant baseline differences. Sampling/recruitment: consecutive-type recruitment of older HF patients admitted to the cardiology department between November 2021 and April 2023, then randomly allocated by random number table; specific sampling method not explicitly named beyond random allocation; number approached/screened NR. Attrition: NR (no participant flow diagram; 52 per group analyzed but withdrawals/loss to follow-up not explicitly reported)

ID	Author, year	Country	Setting	Study design	Aim	Chronic condition(s)	Sample size (N int./control)	Age, sex, and other characteristics
			follow-up after discharge; single site					
14	Fu et al. [33]	China	Geriatric ward of a Grade-A tertiary hospital (Shengjing Hospital of China Medical University, Shenyang, Liaoning) plus eight communities/community health centres in the hospital's service area; post-discharge community and home care delivered via a WeChat-based mHealth platform (telehealth); eight community clusters	Two-arm parallel cluster randomised controlled trial (reported per CONSORT 2010 and its cluster extension); trial registration ChiCTR2400089720	To evaluate the effectiveness of the Smart Post-Discharge Elderly Health Management (SPEM) platform on health-related quality of life and self-management after hospital discharge, and to assess usability, perceived usefulness and economic impact	Older post-discharge adults with chronic conditions/multimorbidity requiring continuous post-discharge care (specific diagnoses not individually reported)	Assessed for eligibility n=3303; excluded n=2903 (not meeting inclusion criteria n=1975; declined n=928); randomised/allocated n=400 across 8 community clusters (4 per group), 200 per arm; received allocated intervention 195 (intervention) and 197 (control); analysed n=192 (intervention) and n=191 (control); analysed cluster sizes intervention 48/47/47/50 and control 48/48/47/48; the text states 383 enrolled with 192/191 allocated	Mean age 73.17 (SD 8.61); intervention 72.83 (SD 8.90), control 73.51 (SD 8.33); male 207 (54.05%) [intervention 109 (56.77%), control 98 (51.31%)], female 176 (45.95%); married 309 (80.68%); living alone 43 (11.23%); receiving pension 282 (73.63%); education, number of children, BADL (Barthel) and IADL (FAQ) balanced; groups well matched at baseline. Sampling/recruitment: cluster randomisation of 8 comparable communities (8000-12000 residents; at least one community health centre; household income RMB 60000-80000/yr) by an independent statistician using SPSS v25.0, 1:1; participants recruited Dec 1 2023-Feb 29 2024 from discharged geriatric-ward patients via flyers, notices and face-to-face contact (eligibility: 60 or older, no cognitive impairment, able to use WeChat, resident in a designated community). Attrition: dropout 4% intervention and 4.5% control (reasons death, unreachable, voluntary withdrawal); per CONSORT, did-not-receive-intervention 5 (died 1, unreachable 3, withdrew 1) and control 4 (died 3, withdrew 1), plus lost-to-follow-up intervention 1/control 2 and discontinued intervention 2/control 4; analysed 192 vs 191 (0 excluded from analysis)
15	Wong et al. [34]	Hong Kong (China)	Two renal centres of a hospital cluster in Hong Kong; outpatient/clinic-based patients on continuous ambulatory peritoneal dialysis (CAPD) returning for clinical follow-up; intervention delivered by post-clinic nurse-initiated telephone follow-up	Randomised controlled trial	To examine whether patients with chronic kidney disease receiving a nurse-led disease management programme have better outcomes than a control group in non-adherence, quality of life, satisfaction, symptom/complication control and health-service utilisation	Chronic kidney disease / end-stage renal failure treated with continuous ambulatory peritoneal dialysis (CAPD); common comorbidities diabetes (33.7%), hypertension (22.4%), heart disease (16.3%)	Planned sample size 94 (47 per group); 120 computer-generated random numbers used; analysis samples reported as control n=49 and study n=49 (total 98 in Tables 1-2); numbers assessed/approached and attrition not explicitly reported	Mean age 62.4 years; male 53.1%, female 46.9%; married 63.3%; education no formal 20.4%, primary 41.8%, secondary 33.7%; employed 9.2%, unemployed 11.2%, retired 53.1%, homemaker 24.5%; housing self-owned 46.9%, public 39.8%, rented 10.2%; finances sufficient 60.2%/more than sufficient 9.2%/insufficient 24.5%/extremely insufficient 6.1%; mean 3.5 years on CAPD; mean 3 daily exchanges; no significant baseline differences except sleep. Sampling/recruitment: all CAPD patients returning to two renal centres for clinical follow-up were invited (consecutive/convenience-type approach; not explicitly named); inclusion required being communicable, alert/oriented, contactable by home telephone and resident in the hospital service area; number invited/screened NR. Attrition: NR (no participant flow numbers or loss to follow-up reported; analysis n=49 per group)
16	Ratanawongsa et al. [35]	USA	Outpatient clinic	RCT	To evaluate the effectiveness of two diabetes self-management support programs (ATSM and GMVs) on quality of care, patient activation,	Type 2 diabetes with poor glycaemic control (HbA1c ≥ 8.0%).	330 patients randomised: 112 ATSM + nurse care management, 113 group medical visits (GMV), 105 usual care. PCP survey completed for 245 patients (74%) by 87 PCPs.	Linguistically diverse adults with poorly controlled type 2 diabetes (HbA1c ≥8.0%); 44% Spanish-speaking, 44% English-speaking and 12% Cantonese-speaking. Patients were recruited from primary care clinics and randomized to ATSM, GMV or usual care. A total of 330 patients were randomized, and PCP survey data were available for 245 participants (74%) from 87 primary care providers.

ID	Author, year	Country	Setting	Study design	Aim	Chronic condition(s)	Sample size (N int./control)	Age, sex, and other characteristics
					and barriers to care in vulnerable adults with diabetes.			
17	Riegel et al. [36]	USA	Transitional (hospital-to-home)	Cluster RCT (physicians randomized)	To assess the effectiveness of a standardized nurse-led telephonic case-management intervention in reducing resource use among patients with chronic heart failure.	Chronic heart failure (HF).	358 patients (130 intervention / 228 usual care).	Mean age 72.5 (SD 13.1) intervention vs 74.6 (SD 12.0) control; female 46.2% vs 53.9%; predominantly NYHA class III-IV (97% overall); 70.0% vs 73.7% English-speaking and 26.9% vs 25.4% Spanish-speaking; high burden of comorbidity. Patients were recruited during hospitalization for heart failure and allocated according to physician-cluster randomization. Of 1,145 patients screened, 573 met eligibility criteria and 358 were enrolled (130 intervention, 228 control). Non-enrolment resulted from refusal to participate (n=148), physician refusal (n=29), withdrawal during follow-up (n=28), and other reasons such as relocation (n=10).
18	Akkus & Akdemir [37]	Turkey	Home care	Quasi-experimental	To determine the effect of NANDA-based nurse home visiting interventions on symptoms and quality of life in patients with multiple sclerosis.	Multiple sclerosis (MS)	45 patients (21 intervention / 24 control)	Mean age 44.1 (SD 7.6) intervention vs 40.2 (SD 9.3) control; female 66.6% vs 62.5%; mean EDSS score 6.96 vs 6.65; most participants lived with family (>95%) and had moderate to severe disability requiring caregiver support. Patients were recruited through the Ankara Multiple Sclerosis Association and allocated according to EDSS score and registration order. Eleven participants withdrew or experienced clinical deterioration during follow-up; final analyses included 21 intervention and 24 control participants.
19	Kizawa et al. [38]	Japan	Primary care	RCT	To examine the effects of a brief nurse-led advance care planning (ACP) intervention using visual materials on goal-of-care preferences, cardiopulmonary resuscitation (CPR) preferences, health care proxy designation, CPR knowledge, and readiness for advance care planning among older adults with chronic diseases.	Chronic diseases, including cardiovascular disease, cancer, pulmonary disease, and renal disease.	220 participants (117 intervention / 103 control).	Mean age 69.5 (SD 3.6) intervention vs 67.1 (SD 6.0) control; 80.3% vs 85.4% male; most participants had college/university education (80.3% vs 88.3%). Participants were aged ≥65 years, were receiving ongoing treatment for at least one chronic disease, and were recruited from a large commercial internet database (Rakuten Research). All enrolled participants completed post-intervention assessments; no attrition was reported.
20	Nualchaem et al. [39]	Thailand	Primary care	Quasi-experimental	To examine the effects of a nurse-led integrated care model on self-management, functional status, blood pressure, and blood glucose outcomes among older adults with uncontrolled chronic diseases living in an urban community setting.	Uncontrolled diabetes mellitus and/or hypertension with multimorbidity.	60 participants (30 intervention and 30 control group).	Community-dwelling adults aged ≥60 years with uncontrolled diabetes mellitus and/or hypertension and multimorbidity; ADL >11 and cognitively intact (MMSE-Thai 2002 score >23). Participants were recruited from two public health centres and allocated by site to intervention (n=30) and comparison (n=30) groups.

Supplementary material: Table S2. Extended data-extraction matrix (Part 2 of 4): characteristics of the nursing intervention.

ID	Author, year	Intervention type	Intervention description (components)	Provider	Duration, intensity, follow-up	Comparator
1	Meekaew et al. 2025	Self-management education / support	12-week nurse-led self-care programme based on Orem's Self-Care Deficit Nursing Theory, delivered through the LINE OA application	Three registered nurses from the NCD clinic, all with a bachelor's	12 weeks; educational content 3 times per week; assessments at	Usual care at the NCD clinic: regular health assessment, treatment and

ID	Author, year	Intervention type	Intervention description (components)	Provider	Duration, intensity, follow-up	Comparator
			(developed with the ADDIE model, pilot-tested with 2 nurses and 10 patients). Week 1: baseline assessment, patient and family education on diabetes, hypertension and CKD stage 3, discussion of identified self-care deficits, goal setting, individualised activity plan and training in use of the application. Weeks 2-5 and 7-11: 5-minute education videos and infographics sent 3 times per week covering diet, medication adherence, exercise and management of abnormal symptoms, each followed by 2 quizzes with manual nurse feedback on wrong answers. Week 6: reassessment plus a session on barriers to self-care. Week 12: final assessment. After-action review through semi-structured exit interviews.	degree in nursing (mean 9.33 years of outpatient experience); one was a certified case manager for diabetes and hypertension. All completed a structured 12-hour training in disease management and programme/application implementation.	baseline, 6 and 12 weeks. No post-intervention follow-up period.	health education based on illness condition. Control participants received the same educational materials and manual after week 12.
2	Zhu et al. 2015	Transitional care	Nurse-led early discharge planning programmes: initial nurse visit within 48 hours of hospital admission, structured pre-discharge assessment of discharge needs, individualised discharge plan, systematic patient and family education, coordination of resources and arranging referrals, followed by structured home visits and telephone follow-up after discharge. Programmes were led by a nurse and supported by a multidisciplinary team; specific trials added case management, volunteer support or an individualised instruction booklet sent to the primary care provider.	Trained nurses, including advanced practice nurses (Naylor 1999, Rawl 1998) and nurse case managers (Wong 2012), supported by multidisciplinary teams of medical experts, physicians, social workers, trained volunteers or family members.	Intervention initiated within 48 hours of admission; trial durations 4 weeks to 12 months; readmission assessed at 1, 3, 6 and 12 months after index discharge.	Standard care, defined as any care in which discharge planning, if provided, was not identified as having been initiated early (within 48 hours of admission); described in trials as usual care, routine discharge nursing care or the regular discharge process.
3	Shaw et al. 2014	Nurse-led clinic / consultation	Nurse-managed protocols in which a registered nurse (or a nurse with equivalent scope of practice) titrated medications by following a written algorithm; 11 of 18 studies also allowed the nurse to independently initiate new medications. Interventions were delivered in a nurse-led clinic in 14 studies, by clinic visits in 83% and primarily by telephone in 3 studies. 16 studies added education or self-management components, 9 included a self-management plan and 3 used motivational interviewing. Intervention targets were glucose (83%), blood pressure (61%) and lipids (50%).	Registered nurses - not advanced practice RNs - in all US studies, and nurses with an equal scope of practice in the non-US studies; no licensed practical nurses were used. Of studies reporting training, 3 used specialists (for example diabetes-certified nurses), 10 used RNs with study-specific training and 1 used nurse case managers experienced in coordinating long-term care. Supervisors were nearly always physicians.	Intervention duration 6 months (2 studies), 12 months (8) and more than 12 months up to 36 months (8); outcomes assessed at 6-36 months, most at 12 months or longer.	Usual care in all but one study, which used a reverse-control design in which each intervention served as the control for the other.
4	Le Berre et al. 2017	Transitional care	Transitional care interventions comprising all of: coordination and continuity of care, pre-arranged structured post-discharge follow-up (home visits, telephone calls) and at least one follow-up beginning within 30 days of discharge. On average interventions started 7.9 (SD 6.2) days after discharge, lasted 179.7 (SD 158.5) days and involved 7.1 (SD 6.3) contacts. Components: telephone contacts (59.2%), home visits (59.8%), telephone availability or hotline (47.8%), educational components such as motivational interviewing, individualised coaching, brochures or videotape (94.6%) and medication management (55.4%), plus multidisciplinary coordination through written reports to primary care physicians or specialists and access to specialists for clinical consultation.	Nurses played a key role in 88.0% of interventions. Other interventions involved pharmacists, physicians, dietitians, physiotherapists or multidisciplinary teams; the subgroup analysis compared nurse-delivered with other configurations.	First contact on average 7.9 days after discharge; mean intervention duration 179.7 days with a mean of 7.1 contacts; outcomes assessed at 1, 3, 6, 12, 18 and 24 months post-discharge.	Usual care, defined as non-structured follow-up. It was sparsely described across trials and generally consisted of pre-discharge education and usual follow-up with the family physician or a specialist as needed.
5	Boult et al. 2008	Case management	Guided Care: a registered nurse who has completed a supplementary chronic-care curriculum is based in a primary care practice and works with two to five physicians to provide comprehensive chronic care to 50-60 multimorbid patients. Using a web-accessible electronic health record the nurse performs eight clinical processes with the patient's physician: assessing the patient and primary caregiver at home, creating an evidence-based care plan, promoting patient self-management, monitoring the patient's conditions monthly, coaching the patient in healthy behaviours,	Registered nurses with at least three years of practice experience (7 nurses), preference given to applicants with gerontological nursing interest or experience, enthusiasm for patient counselling and comfort with information technology and interdisciplinary practice. They completed an	Two-year intervention; nurses conducted on average two home assessments per week until reaching a caseload of 50-60 patients, then monitored patients monthly. This paper reports patient outcomes at six months and physician outcomes at approximately one year.	Usual primary care delivered by the same practices; pods of physicians and their patients were randomised to Guided Care or usual care.

ID	Author, year	Intervention type	Intervention description (components)	Provider	Duration, intensity, follow-up	Comparator
			coordinating transitions between sites and providers of care, educating and supporting the caregiver, and facilitating access to community resources.	educational programme covering comprehensive assessment, evidence-based guidelines, motivational interviewing, collaborative care, chronic disease self-management, elder abuse, cultural competence, community resources, caregiver support and use of the Guided Care electronic health record, with a practicum using simulated patients.		
6	Schenker et al. 2015	Case management	CONNECT (Care Management by Oncology Nurses): an existing oncology clinic nurse delivers a series of nurse-led encounters before or after regularly scheduled oncology visits, at least monthly for three months, performing four primary palliative care roles - addressing symptom needs, engaging patients and caregivers in advance care planning, providing emotional support, and communicating with oncologists and coordinating care. The Edmonton Symptom Assessment Scale and Distress Thermometer are administered before every visit and guide visit content. Visits follow an intervention manual with visit-specific checklists, communication pearls, common pitfalls and sample scripts. An individualised Shared Care Plan is completed and updated at each encounter. Every visit is followed by a mandatory follow-up with the oncologist and a follow-up call to the patient or caregiver within one week.	An existing registered oncology clinic nurse, selected for strong working relationships with clinic providers, interest in learning new skills, commitment to supportive oncology care and preferably Oncology Nursing Society certification. Trained over two days by a palliative care physician and an oncology nurse practitioner using didactic sessions and role-play with simulated patients, followed by biweekly supervision sessions reviewing audiorecorded encounters to prevent skills drift. The nurse received full administrative support and adjusted patient assignments.	At least monthly for three months (three CONNECT visits); mean visit length 29 (SD 15) minutes; three-month outcome assessment. Enrolment January to April 2014.	None - single-arm pilot with no control arm. Comparison is against baseline measures and against descriptions of usual oncology care in the literature; the authors state the trial cannot establish efficacy.
7	Leff et al. 2009	Case management	Guided Care: a registered nurse who has completed a Guided Care Nursing course is based in the primary care practice and works with 2 to 5 physicians and the office staff, providing 8 clinical services to a caseload of 50-60 of the practice's highest-risk older patients: (1) comprehensive assessment at home; (2) creation of an evidence-based care guide; (3) monthly monitoring and coaching of the patient; (4) coordination of the efforts of all the patient's healthcare providers; (5) smoothing transitions between sites of care; (6) promotion of patient self-management; (7) education and support of family caregivers; (8) facilitation of access to community resources.	Registered nurses who had completed a course in Guided Care Nursing, embedded in the practice team alongside 2 to 5 physicians and office staff. Further qualification requirements, prior experience and course length are not reported in this paper.	Guided Care nurses joined their pods in May 2006 and built caseloads of 50-60 patients over the following 6-8 months; one comprehensive home assessment at entry, then monthly monitoring and coaching. This analysis covers each patient's start date through 30 June 2007 (mean exposure 8.3 months intervention vs 7.8 months control); baseline claims window 12 months before the start date. Total trial duration 32 months, with final results published in 2011. Mode and length of individual contacts not reported.	Usual care: patients continued to receive the same care they had received before enrolling, from the same practices and physicians but without a Guided Care nurse. Start dates for control patients were randomly assigned to mirror the distribution of intervention start dates. The content of usual care is not further described.
8	Rechenberg et al. 2021	Self-management education / support	IL2L for Chronic Care: a Spanish-language, telephone-based health coaching intervention delivered on top of standard clinic diabetes care. Each coach was assigned an average of four patients and met intervention participants in person three times (baseline, midpoint, post-intervention). After the baseline meeting, coaches delivered up to six biweekly coaching calls using a semi-structured interview guide. Calls were emergent and participant-led: in each session the participant discussed two health goals, identified barriers to meeting them, discussed readiness for action and confidence, and reviewed progress; coach and participant engaged in collaborative dialogue	Nurse and nurse practitioner students trained in language concordant type 2 diabetes care, acting as language concordant health coaches (proficient in the patients' preferred language, Spanish); mentored by selected bilingual clinical instructors fluent in English and Spanish. Recruitment was supported by a trained on-site	Six biweekly coaching sessions (approximately 12 weeks of active intervention) plus three in-person meetings at baseline, midpoint and post-intervention; completed sessions ranged from three to six, mean 4.6 (SD 1.37); mean session length 18.33 minutes (SD 4.27); coaches made a mean of 1.94 calls (SD 0.64) to reach each participant	Standard diabetes care offered by the clinic, including consultations with a primary care provider, a dietitian, a pharmacist and a diabetes educator, plus written educational materials on diabetes self-management in Spanish. Participants in both groups received incentives (three $10 gas cards and

ID	Author, year	Intervention type	Intervention description (components)	Provider	Duration, intensity, follow-up	Comparator
			and set personal goals. Fidelity: all calls audio-recorded and uploaded to a secure database; bilingual clinical instructors mentored the coaches, reviewed all call notes and action plans, listened to random calls and gave individual feedback throughout delivery.	bilingual promotor (a lay Latinx community member with specialised training in basic health education), who did not deliver the intervention. The coaches were students, not licensed registered nurses.	for each session. Delivered mainly by telephone. Outcomes assessed at baseline, month 3 (A1c) and month 6; total study follow-up 6 months, with no follow-up after that point.	three $10 phone cards) for study-specific clinical visits.
9	Schraeder et al. 2008	Case management	A registered nurse case manager (NCM) and a case assistant were added to each of eight primary care clinics, each pair working with about five primary care physicians and their high-risk health plan patients. Components: an education series for physicians, nurses and administrators on the goals and responsibilities of collaborative care teams, clinical decision making with older adults and outcome reporting; an initial home visit and comprehensive assessment at enrolment; a plan of care developed jointly with the primary care physician; contact or visit at least monthly to review and update the care plan, monitor health status, provide education and arrange health-related services; a comprehensive team review at least every 12 months or sooner on change of status; transitional care, with the NCM visiting during hospital admission or nursing home placement, a post-discharge home visit including medication review, and at least weekly contact for the first month after discharge; ongoing education on community resources, medication management, disease processes and warning symptoms, with materials tailored to the patient; proactive monitoring calls, including calls after physician and specialist visits and after emergency department visits; e-mail alerts to the NCM triggered by an office visit, emergency department visit or hospital admission; monthly feedback reports on patients and panels to the physician-nurse teams; team communication by telephone, voice mail, written notes and summaries, care plan letters to patients and families, informal office conversations and formal team meetings.	Registered nurses working as nurse case managers (5.3 full-time equivalents) supported by case assistants (3.7 FTE), based in eight primary care clinics within a multi-specialty physician group practice and a vertically integrated health system, in collaboration with the patients' primary care physicians. Training was delivered as a joint education series for physicians, nurses and administrators, co-presented at each practice by a physician leader and a nurse administrator. No further nursing qualification or experience requirements are reported.	36-month intervention. Initial home visit and assessment at enrolment; contact or visit at least monthly thereafter; comprehensive team review at least every 12 months; at least weekly contact for the first month after any hospital discharge; additional contacts triggered by e-mail alerts after office, emergency department or hospital use. Delivered through home visits, office visits and telephone. Mean time enrolled 27.6 months (SD 7.9) treatment vs 24.8 (SD 8.6) comparison. Evaluation at enrolment (baseline) and annually at 12, 24 and 36 months; utilisation and cost data collected from each patient's enrolment date to the end of the study period. Duration of individual contacts is not reported.	Usual care in a geographically distinct region (13 counties in west central Illinois), where the same managed care plan contracted with 49 small primary care practices and three urban and six rural hospitals. No nurse case manager was added to these practices. All treatment and comparison patients had the same covered health benefits regardless of where they lived. The content of usual primary care is not further described.
10	Xiao et al. 2022	Psychosocial support / counselling	Family-oriented dignity therapy: a face-to-face psychosocial intervention of three sessions, developed with the Medical Research Council framework for complex interventions and grounded in the dignity model plus the eighth stage of Erikson's model of psychosocial development, and culturally adapted from Chochinov's dignity therapy after two reviews and a qualitative study of dignity perception in Chinese patients. Session 1: introduction of the process and of the nine-question reminiscence protocol (a parallel five-question version for the family caregiver). Session 2: patient and family caregiver together answer the same questions about life history, important roles, accomplishments, life lessons and messages to the family, at the bedside in a curtained-off space or in a private room; audio-recorded and transcribed with the iFlytek Xun Fei Ting Jian application (accuracy above 97.5%), checked by a research assistant against the recording, then edited by the first author into a Spiritual Diary organised by themes (my family, memorable stories, important roles, proudest achievement, life lessons, hopes and dreams, things I want to share with my family, family messages), with family caregivers invited to audiotape messages; a monochrome proof was checked by the family before the final full-colour version with typesetting and photographs. Session 3: patient and family caregiver read the Spiritual Diary together and share their feelings about the reminiscence and the document. A process	The intervention facilitator was the first author, a nursing researcher who had completed a two-and-a-half-day dignity therapy training workshop run by Harvey Max Chochinov in Winnipeg, Canada (theoretical basis, demonstrations, product editing and simulation exercise). A research assistant checked transcripts and recorded the process evaluation. The intervention was not delivered by ward nurses; the authors propose nurse-led delivery as a practice implication, noting the reminiscence process is not time-consuming and can be practised by trained nurses.	Three face-to-face sessions delivered over roughly one week during the inpatient chemotherapy stay. The reminiscence session lasted on average 26.22 minutes (SD 11.53, range 8.24-56.36); the transcript was edited within 24 hours and the Spiritual Diary was usually completed and returned about 48 hours after the reminiscence; the sharing session was then scheduled flexibly according to the patient's physical condition and treatment, face to face or by WeChat video. Outcomes were measured at baseline (T0), after completion of the intervention at one week (T1) and at week four (T2). No follow-up beyond four weeks.	Attention control: usual hospital care plus three attention contacts on a schedule similar to the intervention. The first contact, on the day of admission, informed patients and caregivers of the topics to be covered; the second, the next day, was a health education session on chemotherapy covering preparation, common side effects and management strategies; the third covered nutrition and daily life after discharge. The control condition therefore matched the intervention for contact time and attention but contained no reminiscence or family communication component.

ID	Author, year	Intervention type	Intervention description (components)	Provider	Duration, intensity, follow-up	Comparator
			evaluation with a fidelity checklist, satisfaction scale and semi-structured interviews ran alongside the trial.			
11	Wang et al. 2025	Multicomponent	A structured continuity of care programme added to routine care, with six components. (1) Forming a nursing team: one cardiologist, one nurse, one pharmacist, one nutritionist and one mental health professional, who regularly review the treatment plan and provide collaborative, consistent nursing services. (2) Personalised health education: plans tailored to the patient's condition, cognitive level and educational background, covering the pathophysiology of heart failure, disease monitoring, medication knowledge, the importance of diet and exercise, and recognition of early signs of deterioration. (3) Psychological and social support: assessment of anxiety and depression, psychological intervention where needed, and training in psychological support and stress management skills. (4) Drug management: regular review of the medication plan for rational use and adherence, with pharmacy professionals reviewing prescriptions, adjusting doses and educating patients on effects, side effects and correct use. (5) Regular tracking and monitoring: outpatient follow-up, remote monitoring and telephone support to monitor weight, blood pressure, heart rate and symptoms and to adjust treatment plans in a timely way. (6) Promotion of self-management: encouraging self-monitoring of cardiac function and symptoms and self-regulation of lifestyle, supported by tools such as heart failure diaries and self-monitoring devices. The frequency and duration of contacts are not quantified anywhere in the paper.	A multidisciplinary team containing one nurse alongside a cardiologist, a pharmacist, a nutritionist and a mental health professional. The paper describes the programme as a continuity nursing intervention and frames it as guidance for nursing professionals, but it does not state that a nurse led the programme, nor does it report the nurse's qualifications, training or specific tasks as distinct from those of the other team members.	Duration of the intervention is not stated. Contacts are described only as regular (regular outpatient follow-up, remote monitoring and telephone support) with no frequency, number or length given. Outcomes were assessed before the intervention and at 1 month and 2 months for depression, anxiety, 6-minute walk test, self-efficacy and left ventricular ejection fraction; self-management and quality of life were assessed before and after the intervention with the post-intervention time point unspecified; complications were collected within 3 months after the intervention. No longer-term follow-up.	Routine nursing care following standard heart failure treatment guidelines: guideline-based drug therapy (diuretics, angiotensin-converting enzyme inhibitors, beta-blockers and other agents as indicated); lifestyle education focused on dietary adjustment such as limiting salt intake, weight management and moderate physical activity; and a standard cardiac function monitoring programme including echocardiography and electrocardiogram examinations. The experimental group received all of this plus the continuity of care components.
12	Mallow et al. 2018	Nurse practitioner (APN)-led mHealth/telehealth intervention; remote self-monitoring/telemonitoring; self-management support; patient education; nurse-led video follow-up; care coordination (Chronic Care Model)	mI SMART - HIPAA-compliant web-based platform plus Bluetooth self-monitoring devices (scale, glucometer, BP cuff), a Nexus 7 tablet and 3 months of internet data (kept by participants); components: track diagnoses/medications/lab results; individualised reminders for self-management and medications; self-monitoring with automated immediate feedback plus personal clinician feedback (RN reviewed critical values, referral as needed); secure asynchronous messaging; video conferencing for routine NP visits; synchronous and asynchronous condition-specific education videos and live health-educator sessions; in-person enrollment training by one NP; individualised self-monitoring goals shown green/yellow/red; guided by the Quality Health Outcomes Model and the Chronic Care Model	Nurse practitioner (NP/APN, FNP-BC)-led; a registered nurse (RN) reviewed critical self-monitoring values and referrals; intervention developed by an NP; IT consultant/programmer built the technology; delivered at a single free clinic	12-week intervention; frequency of video visits arranged by patient and NP as needed; self-monitoring frequency individualised per NP recommendation; outcomes measured at baseline (enrollment) and after 12 weeks; no longer-term follow-up	NA (single-group pre/post design; no control or comparison group)
13	Xie et al. 2025	Nurse-led self-management support; supportive-educative nursing (Orem); patient and family education; telephone nursing support; symptom monitoring; psychosocial support	12-week structured self-care programme based on Orem's self-care deficit theory - Step 1 individualised needs assessment with a self-developed Orem-based questionnaire (functional deficits, knowledge gaps, resources, physical/cognitive limits); Step 2 supportive-educative intervention over 2 weeks with three approximately 60-min face-to-face education sessions for patients and families using interactive materials (understanding HF; medication adherence and side effects; self-monitoring of BP/weight/fluid; low-sodium diet, physical activity, smoking cessation; emotional regulation/stress management), individualised teaching plans and tailored counselling; Step 3 follow-up and reinforcement Weeks 3-12 with weekly telephone calls (at least twice weekly) for Q&A and emotional support, weekly patient self-care checklists reviewed by nurses, and additional phone counselling or outpatient visits if gaps identified	Multidisciplinary nursing team - two senior cardiovascular nurses, one heart-failure nurse educator and one clinical psychologist; all completed a standardised 2-day training workshop covering Orem's theory, assessment tools, communication and ethics	12-week programme; education phase three sessions of about 60 min over 2 weeks; reinforcement Weeks 3-12 with at least two telephone calls per week plus weekly checklists; outcomes assessed before and after the 12-week intervention; HF-related readmissions followed for 12 months	Routine/standard nursing care - basic discharge instructions, printed health-education brochures and standard medication counselling per hospital protocol, without structured follow-up or individualised support

ID	Author, year	Intervention type	Intervention description (components)	Provider	Duration, intensity, follow-up	Comparator
14	Fu et al. 2026	Nurse-led digital health/telemonitoring (mHealth); self-management support; patient and caregiver education; transitional/continuity care and care coordination; symptom monitoring; medication reminders	SPEM platform - a WeChat mini-program integrating hospital, community and patient modules with five interconnected feature modules: (1) health monitoring - daily vital-sign input or connected community devices with alerts/abnormality reports to providers; (2) health education - twice-weekly WeChat messages and on-demand disease-management, rehabilitation and lifestyle materials; (3) online consultation - 24/7 text, image or voice access to a multidisciplinary team; (4) medication reminder - daily automated WeChat reminders plus nurse-added personalised reminders; (5) lifestyle management - nurse follow-ups biweekly in month 1 then monthly with personalised diet/exercise/sleep plans; encrypted data transmission; caregivers involved via linked WeChat accounts; developed through an iterative user-centred process led by nurses with a multidisciplinary team; pilot-tested before the trial	Nurse-led (ward, case and community nurses delivered follow-up in both arms); platform developed by nurses with a multidisciplinary team (geriatric specialists, clinicians, software engineers, interface designers); online consultation staffed by multidisciplinary healthcare professionals	6-month intervention; health-education messages twice weekly; lifestyle follow-ups biweekly during month 1 then monthly; assessments at baseline, 3 months and 6 months; recruitment Dec 2023-Feb 2024, follow-up completed Aug 2024	Standardised telephone follow-up by trained nurses at 1 week, 1 month, 3 months and 6 months post-discharge; each 10-15 min call covered medication adherence, symptom monitoring and daily-living support using a structured checklist
15	Wong et al. 2010	Nurse-led disease management / nurse case management; nurse-initiated telephone support; self-management support and health coaching; care coordination (four-Cs model); skill-mix (specialist plus general nurse) model	Routine care plus a protocol-driven disease management programme based on the four-Cs model (comprehensiveness, collaboration, coordination, continuity); each study patient followed by a team of one renal nurse (case manager) and one general nurse; pre-discharge initial assessment using the Omaha System modified for renal patients (four dimensions, 50 health problems); weekly nurse-initiated telephone calls for 6 weeks (first call by the renal nurse within 72 h of clinic discharge, weekly calls for 4 weeks by the general nurse, closing call by the renal nurse); calls structured around greeting/overall health, monitoring changes, health advice, reinforcing self-management, assessing referral need, reviewing and setting mutual goals, and encouragement; Omaha intervention scheme (health teaching and counselling, surveillance, case management); referral to nurse clinic when required	Renal (specialist) nurses acting as case managers and general nurses (recruited as volunteers) in a skill-mix team; renal nurses trained 24 h (8 h theory, 4 h case training, 12 h guided reading), general nurses trained 12 h (8 h theory, 4 h case training); protocols validated by an expert panel (renal clinical nurse specialist, renal physician, renal nurse manager, case manager, academic); simulated-patient training with audiotape review; renal nurse available for consultation	6-week disease management programme with weekly telephone calls; outcomes measured at baseline (O1), 7 weeks / one week after programme completion (O2) and 13 weeks / six weeks after completion (O3)	Routine care only, including instructions on medications and basic health advice
16	Ratanawongsa et al. 2012	Telephone / telehealth follow-up	Weekly automated telephone self-management support with tailored diabetes education messages, assessment of self-management behaviours and health status, nurse care manager follow-up calls for predefined alerts, and collaborative goal setting and action planning, compared with group medical visits and usual care.	RN nurse care manager providing telephone self-management support and follow-up.	9-month intervention; weekly automated telephone calls; nurse follow-up triggered by out-of-range responses. Mean of 22 of 39 calls completed and mean of 9.2 nurse call-backs.	Usual diabetes care provided by primary care clinics, including routine physician care and referral to diabetes education, nutrition counselling or endocrinology services when indicated.
17	Riegel et al. 2002	Telephone / telehealth follow-up	Standardised nurse case-management programme delivered by telephone using decision-support software. Initial contact within 5 days of discharge, ongoing symptom monitoring, patient education, support for medication and dietary adherence, reinforcement of self-management, coordination with family and community services, physician reporting, and monthly educational materials.	Registered nurse case managers trained in heart failure management and use of a decision-support software system; supported by ongoing mentoring.	6-month intervention; mean 17 telephone contacts per patient (median 14, IQR 11-22); approximately 16 hours of nurse time per patient; outcomes measured at 3 and 6 months.	Usual post-discharge care; no structured telephonic case-management programme.
18	Akkus & Akdemir 2011	Home visits	NANDA-based nurse-led home visiting programme covering assessment, individualised care planning, symptom monitoring, patient and family education, counselling, psychosocial support, risk assessment, self-care support, and referral to other health professionals when necessary. Eight structured home visits over four months.	MS-specialized registered nurse trained in NANDA nursing assessment and care planning.	8 home visits over 4 months; first four weekly, visits 5-6 biweekly, visits 7-8 monthly; each visit 60-90 minutes.	Usual care plus two study visits (baseline and final assessment); an educational booklet provided at study completion.
19	Kizawa et al. 2020	Psychosocial support / counselling	Brief nurse-led advance care planning discussion covering goals of care, life-sustaining treatment, CPR preferences, and health care proxy designation. The intervention group additionally received visual materials (PowerPoint images illustrating CPR, intensive care, life-prolonging care, and comfort care scenarios), while the control group received standardized verbal explanations only.	Six registered nurses with at least five years of oncology experience; one was a certified oncology nurse.	Single 30-minute face-to-face intervention; outcomes assessed at baseline and immediately after intervention.	Standardized nurse-led verbal advance care planning discussion without visual materials.

ID	Author, year	Intervention type	Intervention description (components)	Provider	Duration, intensity, follow-up	Comparator
20	Nualchaem et al. 2026	Case management	12-week Nurse-Led Integrated Care Model comprising: comprehensive geriatric assessment and goal setting; health education and skill training; monthly multidisciplinary home visits; bi-weekly self-management support from a nurse case manager via a self-monitoring application; two group-sharing sessions; ongoing monitoring and feedback.	Nurse case manager coordinating integrated care, self-management support and outcome monitoring in collaboration with a multidisciplinary team.	12 weeks; monthly multidisciplinary home visits; bi-weekly nurse follow-up; self-monitoring application with patient reporting three days per week and weekly nurse feedback; outcomes assessed at baseline and week 12.	Usual care provided by a public health centre.

Supplementary material Table S3. Extended data-extraction matrix (Part 3 of 4): targeted care gaps and reported outcomes.

ID	Author, year	Care gap type	How the gap was measured	Effect on the gap	Outcome category	Measurement instrument(s)	Time points	Results	Direction of effect	Statistical vs. clinical significance
1	Meekaew et al. 2025	Care deficit	Measured directly. The targeted self-care deficit was measured with the researcher-developed Self-Care Agency Scale and Self-Care Behaviour Scale (together with a Knowledge Assessment Scale) at baseline, 6 and 12 weeks and compared between groups. The service-level gap (limited number of diabetes care and education specialists, nurse workload, inadequate follow-up of patients' self-care) is described narratively in the introduction and explored in the after-action review interviews.	Reduced: significant between-group improvement in self-care agency and self-care behaviour at 6 and 12 weeks	Multiple	Researcher-developed Knowledge Assessment Scale (10 dichotomous items, KR-20 = 0.70); Self-Care Agency Scale (10 items, 5-point Likert, alpha = 0.68); Self-Care Behaviour Scale (15 items, 5-point Likert, alpha = 0.70); Client Satisfaction Scale (10 items, alpha = 0.78); A1C from venous blood (hospital laboratory); blood pressure by calibrated sphygmomanometer; microalbuminuria (MAU) from first-morning urine. Semi-structured exit interviews analysed thematically by 3 researchers. Analyses in SPSS 18.0: paired and independent t-tests, repeated measures ANOVA, Cohen's d.	Baseline, 6 weeks and 12 weeks (A1C and MAU at baseline and 12 weeks only); exit interviews after completion of the programme.	Between groups: knowledge, self-care agency and self-care behaviour were significantly higher in the intervention group than in the control group at 6 and 12 weeks. Within-group change, intervention: knowledge +1.71 at 6 wk and +2.89 at 12 wk (both p < .001); self-care agency +7.89 and +8.31 (p < .001); self-care behaviour +7.54 and +10.63 (p < .001); Cohen's d 0.87-2.29 (large). SBP -13.31 at 6 wk and -20.85 at 12 wk (p < .001); DBP -9.19 and -11.04 (p = .029); control unchanged except DBP +2.02 at 12 wk (p = .010); between-group d 1.37-2.02. MAU -36.12 in intervention (p < .001), no change in control; between-group difference -34.12 (p = .005), d = 0.19. A1C -0.61 in intervention (p < .001), no change in control; between-group difference -0.65 (p < .001), d = 0.35. Clinically relevant A1C reduction >= 0.5%: 52.8% (n = 29) intervention vs 5.7% (n = 3) control; A1C increased/exacerbated in 1.8% (n = 1) vs 45.3% (n = 24). MAU reduced in 43.6% (n = 24) vs 11.3% (n = 6); MAU increased/exacerbated in 0% vs 18.9% (n = 10).	Positive (significant)	Both reported and distinguished. Effect sizes were large for knowledge, self-care and blood pressure but small for A1C (d = 0.35) and MAU (d = 0.19) despite statistical significance; the authors therefore report the clinically meaningful threshold separately (A1C reduction >= 0.5% achieved by 52.8% vs 5.7%).
2	Zhu et al. 2015	Continuity of care	The gap was not measured with a dedicated instrument. It was operationalised as failure of the hospital-to-community transition and quantified indirectly through index length of stay, all-cause readmission, readmission length of stay and all-cause mortality,	Not directly measured (readmission, length of stay and mortality treated as indirect evidence)	Multiple	Outcomes extracted from the primary trials: hospital records for length of stay, readmission and mortality; Minnesota Living with Heart Failure Questionnaire and the eight SF-36 subscales for quality of life; author-developed Likert	Index admission; 1, 3, 6 and 12 months after index discharge (readmission subgroups); trial follow-up 4	Length of stay (5 trials): SMD 0.03 (95% CI -0.06 to 0.12), p = 0.54, I2 = 0% - no difference. Hospital readmission (10 trials): RR 0.72 (95% CI 0.58-0.89), p = 0.002, I2 = 66%, a 28% relative reduction; fixed-effect model RR 0.72 (0.64-0.80). Subgroups by follow-up: no difference at 1 month (RR 0.73, 0.46-1.15, p = 0.17)	Mixed	The authors translate the pooled estimates into practice terms - a 28% reduction in readmissions and a 30% reduction in all-cause mortality with significant resource

ID	Author, year	Care gap type	How the gap was measured	Effect on the gap	Outcome category	Measurement instrument(s)	Time points	Results	Direction of effect	Statistical vs. clinical significance
			with narrative analysis of cost, quality of life and satisfaction.			satisfaction scales; costs in local currencies. Risk of bias assessed with the Cochrane tool across six domains by two independent reviewers; meta-analyses in RevMan 5.2.6 using Mantel-Haenszel risk ratios and inverse-variance mean or standardised mean differences.	weeks to 12 months.	or 3 months (RR 0.64, 0.33-1.27, p = 0.20), significant at 6 months (RR 0.48, 0.37-0.63, p < 0.001, I2 = 0%) and 12 months (RR 0.76, 0.65-0.88, p < 0.001, I2 = 0%). By age: >= 65 years RR 0.74 (0.57-0.96), p = 0.02; < 65 years RR 0.69 (0.51-0.92), p = 0.01. Readmission length of stay (2 trials): MD -2.08 days (95% CI -3.76 to -0.39), p = 0.02, I2 = 0%. All-cause mortality (5 trials): RR 0.70 (95% CI 0.52-0.95), p = 0.02, I2 = 0%, a 30% relative reduction. Narrative: total cost lower (USD 0.6 vs 1.2 million at 24 weeks, p < 0.001; saving of EUR 2063 per patient; cost-benefit ratio 1.09); readmission cost lower (saving EUR 2505 per patient; HK$1505 and HK$3000); quality of life significantly better in 2 trials; satisfaction showed no significant between-group difference in any of the 3 trials reporting it.		implications - but statistical caution is warranted: readmission heterogeneity was substantial (I2 = 66%), readmission length of stay rests on only 2 trials, and the funnel plot for length of stay was asymmetrical, suggesting possible publication bias.
3	Shaw et al. 2014	Quality of care	The gap was not measured with a dedicated instrument. It is framed in the background as the difference between recommended and delivered care, compounded by the shortage of primary care clinicians, and was operationalised through guideline-target attainment (proportion of patients reaching target blood pressure and total cholesterol), diabetes performance measures (eye, foot and urinary microalbumin-creatinine examinations) and nurses' documented adherence to treatment protocols compared with usual care.	Not directly measured (intermediate clinical outcomes and target attainment treated as indirect evidence)	Multiple	A1C (%), systolic and diastolic blood pressure (mm Hg), total and LDL cholesterol (mmol/L and mg/dL), target attainment expressed as odds ratios, patient behavioural adherence, nurse protocol adherence, adverse effects and resource use (salary, medication, inpatient and outpatient costs). Dichotomous outcomes combined as odds ratios and continuous outcomes as mean differences in random-effects models with the Knapp-Hartung adjustment; heterogeneity by Cochran Q and I2; strength of evidence graded with the AHRQ approach.	6 to 36 months; most studies reported outcomes at 12 months or longer.	A1C: -0.4% (95% CI 0.1 to 0.7), n = 8, Q = 23.19, I2 = 70% (moderate strength of evidence). Systolic BP -3.68 mm Hg (CI 1.05 to 6.31) and diastolic BP -1.56 mm Hg (CI 0.36 to 2.76), 12 RCTs, 10 224 patients, I2 > 70% (moderate SOE). Total cholesterol -0.24 mmol/L (9.37 mg/dL) (CI -0.54 mmol/L to +0.05 mmol/L), n = 9 - not statistically significant; LDL cholesterol -0.31 mmol/L (12.07 mg/dL) (CI -0.73 to +0.11 mmol/L), n = 6, I2 >= 89% (low SOE). Target attainment: total cholesterol OR 1.54 (CI 1.02-2.31), equating to 106 more patients per 1000 reaching target (CI 5 to 196); blood pressure OR 1.41 (CI 0.98-2.02) - not significant, 120 more patients per 1000 (CI 6 fewer to 244 more); reduction in the proportion with A1C >= 8.5% OR 1.69 (CI 1.25-2.29) in one study. Medication adherence 80.5% +/- 23.0% vs 69.2% +/- 31.1% (p = 0.03) in one study. Adverse effects: severe hypoglycaemia 3.1% intervention vs 2.9% control (p = 0.158), one study. Resource use: salary costs EUR 114.6 +/- 50.4 vs 138.3 +/- 48.3 (p < 0.001); inpatient costs USD 869 535 vs 1 702 682 (p = 0.02). Subgroup: greater A1C effect in US studies (-0.92 vs -0.23, p = 0.01).	Mixed	Statistically significant for A1C and blood pressure with moderate strength of evidence, but the absolute changes are modest (0.4% A1C, about 4/2 mm Hg) and the authors do not claim clinical superiority. Cholesterol reductions were not statistically significant, and the strength of evidence was judged insufficient for protocol adherence, adverse effects and resource use. Heterogeneity was substantial throughout (I2 70-90%) and subgroup analyses explained little of it.
4	Le Berre et al. 2017	Continuity of care	The gap was not measured with a dedicated instrument. It is documented in the background from the literature on failed information exchange at discharge - primary care physicians receiving incomplete, late or no	Not directly measured (mortality and service use treated as indirect evidence)	Multiple	All-cause mortality, all-cause hospital readmission, all-cause emergency department visits and total readmission days as reported in the primary trials. Quality of life pooled where the Minnesota Living with	1, 3, 6, 12, 18 and 24 months after discharge.	Mortality (68 trials): significantly lower at 3 months RD -0.02 (-0.05, 0.00), NNT 50; 6 months RD -0.02 (-0.03, 0.00), NNT 50; 12 months RD -0.04 (-0.06, -0.01), NNT 25; 18 months RD -0.07 (-0.12, -0.02), NNT 14; not significant at 1 month. Emergency department visits (20	Mixed	The authors argue clinical relevance through numbers needed to treat (7 for readmission at 3 months, 14-50 for mortality), but the

ID	Author, year	Care gap type	How the gap was measured	Effect on the gap	Outcome category	Measurement instrument(s)	Time points	Results	Direction of effect	Statistical vs. clinical significance
			discharge summaries, and patients reporting a lack of clear information and follow-up - and quantified indirectly through post-discharge service use and mortality.			Heart Failure questionnaire, EQ-5D, SF-12 or SF-36 were used, with narrative synthesis for other scales. Cochrane risk-of-bias tool for study quality; RevMan 5.3.5 random-effects models producing risk differences with number needed to treat and mean differences with 95% CI; funnel plots, subgroup and sensitivity analyses.		trials): lower only at 3 months RD -0.08 (-0.15, -0.01), NNT 13; no significant difference at 1, 6 or 12 months and no difference in the mean number of visits. Readmission (76 trials): RD -0.08 (-0.14, -0.03), NNT 7 at 3 months; RD -0.05 (-0.09, 0.00), NNT 20 at 6 months; RD -0.11 (-0.17, -0.05), NNT 9 at 12 months; RD -0.11 (-0.21, -0.01), NNT 9 at 18 months; no difference at 1 month. Mean number of readmissions lower only at 24 months, MD -1.03 (-1.81, -0.24). Total readmission days (41 trials): MD -1.33 (-2.15, -0.52) at 3 months; -1.42 (-2.33, -0.50) at 6 months; -3.37 (-5.21, -1.53) at 12 months; -3.16 (-5.68, -0.64) at 18 months; no difference at 1 month. Quality of life: no significant differences on pooled MLHF total or subscales at 2, 3, 6 and 12 months, EQ-5D visual analogue scale at 3 and 6 months, or SF-12 components at 6 months; among SF-36 subscales at 6 months only Physical Functioning differed. Subgroups favouring better outcomes: telephone calls, 24/7 telephone availability and pharmacist involvement; NOT favoured: nurse-led delivery, medication reconciliation, home visits, telemonitoring, first contact within 1 week, and a CHF-specific population. European interventions appeared to perform better.		absolute risk differences are small (2-11 percentage points). Sensitivity analyses removing prorated data, imputed standard deviations or studies with at least one item at risk of bias caused several effects to lose statistical significance (mortality and readmissions at 18 months, readmission days at 3 and 18 months). Data were sparse below 3 months and beyond 18 months.
5	Boult et al. 2008	Quality of care	Measured directly. The Patient Assessment of Chronic Illness Care (PACIC), a validated 20-item instrument covering goal setting, coordination of care, decision support, problem solving and patient activation, administered at baseline and six months by trained interviewers masked to group assignment; responses dichotomised into higher quality (process occurred most of the time or always) versus lower quality. Supplemented by anonymous physician surveys including ten items derived from the Primary Care Assessment Tool on knowledge of patients and care coordination processes.	Reduced: significant between-group improvement in the aggregate PACIC score (adjusted OR 2.03) and in three of five subscales	Quality of care (process indicators)	PACIC with five subscales and an aggregate score; physician surveys on satisfaction with 11 aspects of care (1-6 scale), time spent on 5 tasks (1-5), knowledge of 6 elements of patient information (1-4) and occurrence of 4 care coordination processes (1-4); a validated nurses' job satisfaction instrument; SF-36 Physical and Mental Component Summaries, hierarchical condition category score and Short Portable Mental Status as covariates. Multivariate logistic regression adjusted for baseline sociodemographic characteristics, health and functional status, baseline PACIC, satisfaction and practice site; multiple imputation with chained equations for missing baseline items.	Baseline (face-to-face) and six months (telephone, interviewers masked) for patients; baseline and approximately one year for physicians; one year for nurses.	Aggregate PACIC rated high quality by 17.4% Guided Care vs 8.5% usual care; adjusted OR 2.03 (95% CI 1.22-3.39), p = 0.006. Goal setting 24.6% vs 11.6%, aOR 2.37 (1.53-3.67), p < 0.001. Coordination of care 14.2% vs 7.1%, aOR 2.25 (1.27-3.97), p = 0.005. Decision support 42.7% vs 33.1%, aOR 1.51 (1.08-2.10), p = 0.014. Problem solving 33.4% vs 24.7%, aOR 1.35 (0.95-1.93), p = 0.096 - not significant. Patient activation 26.6% vs 23.0%, aOR 1.06 (0.74-1.52), p = 0.763 - not significant. Physicians (mean difference Guided Care minus usual care): satisfaction with communicating with patients 0.67 (0.09-1.25), p = 0.026; communicating with family caregivers 0.78 (0.12-1.45), p = 0.023; educating family caregivers 1.08 (0.30-1.87), p = 0.009; motivating patients 0.89 (0.29-1.49), p = 0.005; referrals to community resources 0.83 (0.04-1.61), p = 0.039; knowing all medications patients take 0.54 (0.01-1.07), p = 0.045; trend for giving patients written information for specialists 0.89 (-0.06 to 1.83), p = 0.065.	Positive (significant)	The doubling of the odds of rating care as high quality is substantial in relative terms, but the absolute rates remained low in both arms (17.4% vs 8.5%), and the two subscales closest to patient behaviour change - problem solving and patient activation - showed no effect. The authors state explicitly that conclusions about efficiency, effectiveness, timeliness, technical proficiency, quality of life, functional independence, cost and caregiver strain cannot be inferred

ID	Author, year	Care gap type	How the gap was measured	Effect on the gap	Outcome category	Measurement instrument(s)	Time points	Results	Direction of effect	Statistical vs. clinical significance
								Six of seven nurses were moderately or very satisfied with the model of care. Follow-up rates 93.8% and 93.2%.		from these six-month data.
6	Schenker et al. 2015	Unmet needs	The gap was not measured with a dedicated instrument. Unmet primary palliative care needs are documented in the background (41% of NCI and 78% of non-NCI cancer centres have no outpatient palliative care clinic; the nearest specialty palliative care clinic was 4.5 miles from the study site, with no on-site service) and operationalised through visit checklists recording whether each palliative care process occurred, the documented content of oncologist check-in sessions, and three-month questionnaires on whether the intervention helped with symptoms, coping and planning.	Not directly measured (single-arm feasibility study; perceived effectiveness reported descriptively)	Patient satisfaction / experience	Feasibility: enrolment rates, visit checklists and three-month outcome assessment rates. Fidelity: protocol adherence plus analysis by the principal investigator of 18% of audiorecorded encounters. Patient outcomes collected but not analysed for change: FACIT-Pal quality of life (46 items, range 0-184), Edmonton Symptom Assessment Scale (9 items, range 0-900), Hospital Anxiety and Depression Scale (14 items, cut-off >= 8). Caregiver outcomes: HADS and Zarit Caregiver Burden short form (12 items). Acceptability and perceived effectiveness by three-month questionnaires. Data managed in REDCap; descriptive statistics only.	Baseline and three months; documentation at each of the three CONNECT visits and at each oncologist check-in.	Feasibility: consent-to-approach rate 86%, enrolled-to-consent rate 77%; CONNECT implemented according to protocol for all participants; no participant withdrew after enrolment; three-month assessments completed with all remaining participants (83%); visits in person 92% and by telephone 8%; 92% on the same day as a scheduled oncology visit; mean overall quality score 84% and mean content score 96% across 11 audiorecorded encounters; missing data about 1%. Perceived effectiveness (n = 34): satisfied with the sessions 32 (94%); improved pain or other symptoms 29 (85%); better understanding of the illness 30 (88%); helped to cope 31 (91%); helped to plan for the future 28 (82%); would recommend to others 33 (97%). All 5 oncologists agreed CONNECT improved the quality of care, found it helpful for symptoms, advance care planning, emotional support and coordination, and were comfortable working with the CONNECT nurse. Oncologist check-ins covered physical symptoms 98%, emotional symptoms or distress 94%, readiness to think about the future 58%, goals of care 45%, treatment preferences 39%, questions for the oncologist 34%, decision-making preferences 32% and possible care transitions 18%; in 33 of 62 check-ins (53%) the nurse requested a new treatment plan and the oncologist was receptive in every case. Baseline scores: FACIT-Pal 140.7 (24.9); ESAS 216.7 (128.9); patient HADS anxiety 5.5 (3.9) and depression 4.6 (3.4); caregiver burden 10.7 (9.2); caregiver HADS anxiety 7.7 (5.3) and depression 4.0 (5.6). Three-month mortality 17% (4 patients). NO effect estimates, p-values or confidence intervals are reported - the design does not permit them.	Not applicable (feasibility study; effectiveness not tested)	Neither can be assessed: no statistical testing of change was performed and only descriptive statistics (counts and means) are reported. The perceived effectiveness rates of 82-97% are high but come from an uncontrolled sample of 34 respondents who knew they were receiving an intervention. The authors state explicitly that the trial does not establish efficacy and that a randomised controlled trial is needed.
7	Leff et al. 2009	Fragmented care	The gap was not measured with a dedicated instrument. It was operationalised through claims-based use of 9 service categories (hospital, skilled nursing facility, primary care physician, specialty physician, home healthcare, emergency department, durable medical equipment, tests, non-	Not directly measured (non-significant differences in service use treated as indirect evidence)	Multiple	Paid insurance claims from 3 insurers (administrative data, no questionnaire); claims-based Hierarchical Condition Category (HCC) predictive model for risk stratification and adjustment; pre-randomisation in-home baseline interviews	Baseline: 12 months of claims before each patient's start date, plus a pre-randomisation in-home interview. Outcomes: from each patient's	Adjusted ratios of Guided Care to usual care service use: hospital days 24% fewer (95% CI 49% fewer to 13% more); skilled nursing facility days 37% fewer (95% CI 65% fewer to 5% more); home healthcare episodes 29% fewer (95% CI 53% fewer to 8% more); emergency department visits 15% fewer (95% CI 38% fewer to 18% more); specialist visits	Positive (trend)	Not statistically significant: the authors state explicitly that the observed differences did not meet the traditional criterion of $p < 0.05$, and every 95% confidence

ID	Author, year	Care gap type	How the gap was measured	Effect on the gap	Outcome category	Measurement instrument(s)	Time points	Results	Direction of effect	Statistical vs. clinical significance
			medication treatments) and through aggregate costs, on the assumption that proactive coordinated care reduces avoidable acute and post-acute use. Coordination, continuity, transitions, self-management and caregiver support were not measured directly in this paper (measured with the PACIC in the companion paper, ID 5).			(instrument not named); Centers for Medicare and Medicaid Services national average payment rates per unit of service and mid-Atlantic nurse salaries for costing.	start date through 30 June 2007 (mean 8.3 vs 7.8 months), annualised as mean units of service per person per year. No intermediate time points; 2-year outcomes reported elsewhere (2011).	9% more (95% CI 8% fewer to 29% more). Any use of the service, intervention vs control: hospital 26% vs 30%, home health 20% vs 22%, skilled nursing facility 8% vs 9%. Costs: net saving of $75,000 per nurse per year for a caseload of 55 patients (95% CI -$244,000 to $150,900), about $1364 per patient, two-thirds of it from reduced hospital use. No p values are reported for individual utilisation outcomes and no effect sizes are given.		interval crosses the null, including the cost estimate. Clinical significance was not assessed and no minimal clinically important difference was defined. The authors present the estimated saving as potentially important for policy but caution that the 8-month findings are imprecise.
8	Rechenberg et al. 2021	Health literacy / information gap	The gap was not measured with a dedicated instrument. Language discordance is documented in the background as a communication and education gap: people with LEP are three times more likely to misunderstand a medical situation described by their provider and 4 to 5 times more likely to report confusion about medication and difficulty understanding prescription labels. In this study it was operationalised indirectly through feasibility metrics (response rate, attrition, sessions completed, session length, calls needed per session), acceptability (PSCC satisfaction) and preliminary trends in A1c, PHQ-9 and GAD-7. Language concordance, comprehension and unmet information needs were not measured directly, and no between-group testing of outcomes was performed.	Not directly measured (feasibility, acceptability and within-group clinical changes only)	Multiple	A1c from medical record review, measured at the clinic visit with DCA2000 (Bayer, NY); Spanish version of the Generalized Anxiety Disorder-7 (GAD-7; 7 items, 4-point Likert, range 0-21, higher = more severe, alpha 0.89); Spanish version of the Patient Health Questionnaire-9 (PHQ-9; 9 items, range 0-27, higher = more severe, alpha 0.88); Patient Satisfaction with Cancer-Related Care scale (PSCC) adapted for type 2 diabetes and health coaching (18 items, 5-point Likert, range 18-90, alpha 0.81; the Measures section states that lower scores indicate greater satisfaction); self-reported demographics; BMI by physical examination or record review; author-defined feasibility metrics.	Baseline, month 3 (A1c only) and month 6. The Measures section states that self-report measures were completed at baseline and at 6-month follow-up; PSCC satisfaction was collected from intervention participants after the coaching sessions.	Feasibility: 18 of 22 enrolled (82% response rate), attrition 6% (n = 1), mean 4.6 of 6 sessions completed (SD 1.37, range 3-6), mean session length 18.33 min (SD 4.27), mean 1.94 calls (SD 0.64) to reach a participant per session. Acceptability: PSCC mean 82.9 (SD 6.4), completed by all 10 intervention participants. A1c, intervention (n = 10): baseline 10.9 (SD 2.02), month 3 9.42 (SD 1.93), month 6 8.96 (SD 2.26); paired t baseline to month 3 = 5.41, p < .01, d = 1.70; month 3 to month 6 t = 3.14, p < .05, d = 1.05; baseline to month 6 t = 6.17, p < .001, d = 2.05. A1c, control (n = 7): baseline 10.41 (SD 1.22), month 3 10.37 (SD 1.18), month 6 10.14 (SD 1.04); all paired t-tests non-significant (0.12; 2.15; 1.85). PHQ-9, intervention (n = 9, one outlier deleted): 14.1 (SD 11.3) to 10.5 (SD 1.7), t = 4.34, p < .001, d = 1.30 (the text reports t = 3.20). GAD-7, intervention (n = 9): 10.1 (SD 4.24) to 7.01 (SD 5.65), t = 4.22, p < .01 per the table (the text reports p < .001), d = 1.48. All outcome tests are within-group dependent t-tests; no between-group comparisons were performed and no confidence intervals are reported.	Within-group change only (no between-group test)	Statistically significant within-group improvements in the intervention group only, with large effect sizes (d = 1.05 to 2.05), and no significant change in the control group; however, no between-group tests were performed, so a treatment effect cannot be established. The authors claim clinically meaningful differences, pointing to an A1c fall of at least 1 percentage point at 3 months and about 2 percentage points at 6 months, a 3.6-point fall in depression scores and a 3-point fall in anxiety scores, but no minimal clinically important difference was defined and the authors state that the sample was too small to determine efficacy.
9	Schraeder et al. 2008	Fragmented care	The gap was not measured with a dedicated instrument. The authors frame it in the background as repeated fragmentation of medical and community-based services for older people with chronic illness, and a healthcare system poorly suited to the needs of chronicity, with particular vulnerability to	Not directly measured (hospital use treated as indirect evidence)	Multiple	Author-developed 50-item mailed health questionnaire (self-report, with telephone interview for non-responders) covering demographics, health and functional status, chronic conditions, ADL and IADL limitations, durable medical equipment use and prior 6-	Enrolment (baseline) and annually at 12, 24 and 36 months. Utilisation and cost data covered each patient's full enrolment period (mean 27.6 vs	Unadjusted (treatment n = 400 vs comparison n = 277): any hospital admission 51.0% vs 53.8%, p = 0.352; two or more hospital admissions 19.2% vs 28.8%, p = 0.006; hospitalisations among those hospitalised 1.76 (SD 1.27) vs 2.30 (SD 1.83), p = 0.001; hospital bed days among those hospitalised 8.19 (SD 10.15) vs 13.89 (SD 16.54), p = 0.002; any	Mixed	Statistically significant for repeat and total hospital use: 34% lower odds of two or more hospitalisations (p = 0.032), 0.5 fewer hospitalisations (p = 0.002) and 5.3 fewer

ID	Author, year	Care gap type	How the gap was measured	Effect on the gap	Outcome category	Measurement instrument(s)	Time points	Results	Direction of effect	Statistical vs. clinical significance
			preventable rehospitalisation. It was operationalised through health plan claims: the likelihood of any hospital admission, of two or more hospital admissions, the number of hospitalisations and hospital bed days among those hospitalised, emergency department visits without a subsequent hospitalisation, emergency department visits among users, and total cost of care per patient per month including the cost of the intervention. Coordination, continuity and unmet needs were not measured directly.			month service use; risk screening based on 24 selected questions from that questionnaire (screening process published previously); managed care plan administrative claims for enrolment, provider, utilisation and billed charges, with charges converted to Medicare allowable costs. No validated patient-reported outcome measures were used.	24.8 months) up to the end of the 36-month study.	emergency department visit without hospitalisation 16.8% vs 12.1%, p = 0.086; emergency department visits among users 1.48 (SD 0.87) vs 1.79 (SD 1.20), p = 0.135; cost of care per patient per month $1193 (SD $1953) vs $708 (SD $1028), p < 0.001. Adjusted (covariates: age, gender, minority race, less than high school education, lives alone, cancer, no annual physical, any specialist visit, months enrolled, propensity score): any hospital admission OR 0.93 (95% CI 0.67-1.30), p = 0.683; two or more hospital admissions OR 0.66 (95% CI 0.45-0.97), p = 0.032, a 34% reduction; hospitalisations among users B = 0.54 (95% CI 0.89 to 0.20), p = 0.002, about 0.5 fewer admissions; hospital bed days among users B = 5.25 (95% CI 8.23 to 2.27), p = 0.001, about 5.3 fewer days; any emergency department visit without hospitalisation OR 1.39 (95% CI 0.87-2.25), p = 0.173; emergency department visits among users B = 0.32 (95% CI 0.81 to 0.18), p = 0.205; cost of care $106 per patient per month lower in the treatment group (95% CI $138 to $75), p = 0.253. Intervention cost $54 per patient per month, $1491 per patient on average.		bed days (p = 0.001) among those hospitalised. Not statistically significant for the likelihood of any hospital admission, any emergency department visit without hospitalisation, emergency department visits among users, or cost of care ($106 per patient per month, p = 0.253). The authors give the cost result a practical rather than statistical interpretation, describing it as budget neutral and more than enough to offset the $54 per patient per month cost of the intervention. No minimal clinically important difference was defined.
10	Xiao et al. 2022	Unmet needs	The gap was not measured with a dedicated instrument. It is framed as unmet psychosocial, existential and spiritual needs during chemotherapy for lung cancer (dignity-related distress from changed appearance, feeling a burden, loss of roles and loss of meaning), compounded by the absence of a culturally appropriate dignity intervention for Chinese patients, since dignity therapy was developed in Canada. It was operationalised through validated distress and well-being scales (Patient Dignity Inventory, PHQ-9, FACIT-Sp) and, for reach and acceptability, through a process evaluation covering intervention integrity, participant involvement, satisfaction and interview accounts.	Not directly measured (dignity-related distress, depression and spiritual well-being treated as indirect evidence)	Multiple	Patient Dignity Inventory (PDI; 25 items, five subscales, each item 1-5, total range 25-125, higher = greater dignity-related distress; Cronbach's alpha 0.93 total and 0.63-0.83 for subscales; validated Chinese version alpha 0.92); Patient Health Questionnaire-9 (PHQ-9; 9 items, 0-3, range 0-27, higher = more depressive symptoms; simplified Chinese version alpha 0.86); Functional Assessment of Chronic Illness Therapy - Spiritual Well-being Scale (FACIT-Sp; 12 items over peace, meaning and faith, 0-4, range 0-48, higher = better spiritual well-being; Chinese version alpha 0.83); Satisfaction with Therapy and Therapist Scale (12 items, two domains); a self-designed demographic and clinical questionnaire with medical record review; a team-designed intervention integrity	Baseline (T0), one week / immediately after completion of the intervention (T1) and week four (T2). Satisfaction questionnaires were completed within two days of finishing the intervention.	Generalised estimating equation group-by-time interaction terms (intervention vs attention control, change from baseline). At T1: total PDI beta -3.347 (95% CI -6.054 to -0.641), p = 0.015; existential distress beta -1.372 (95% CI -2.269 to -0.472), p = 0.003; PHQ-9 beta -3.430 (95% CI -5.032 to -1.829), p < 0.001; FACIT-Sp beta 3.705 (95% CI 0.599 to 6.811), p = 0.019; meaning subscale p = 0.002; faith subscale beta 1.616 (95% CI 0.363 to 2.869), p = 0.012. Not significant at T1: symptom distress beta -0.580 (95% CI -1.849 to 0.688), p = 0.370; peace of mind beta -0.422 (95% CI -0.944 to 0.099), p = 0.113; peace subscale p = 0.432. At T2 only spiritual well-being remained significant: FACIT-Sp beta 4.939 (95% CI 0.476 to 9.401), p = 0.030, with the faith subscale beta 2.334 (95% CI 0.633 to 4.034), p = 0.007; total PDI beta -0.919 (95% CI -5.625 to 3.786), p = 0.702; existential distress p = 0.660; PHQ-9 beta -1.728 (95% CI -4.030 to 0.574), p = 0.141. Descriptive means, intervention vs control at T0 / T1 / T2: PDI 38.23, 35.98, 36.64 vs 36.15, 38.10,	Mixed	Statistically significant at one week for total dignity-related distress, existential distress, depression and spiritual well-being, and at four weeks for spiritual well-being only; not significant for symptom distress or peace of mind distress at any point, and not significant for dignity-related distress or depression at four weeks. The authors are explicit that statistical significance does not establish clinical significance here: the total PDI fell by only 2.25 points (38.23 to 35.98) in the

ID	Author, year	Care gap type	How the gap was measured	Effect on the gap	Outcome category	Measurement instrument(s)	Time points	Results	Direction of effect	Statistical vs. clinical significance
						checklist; and semi-structured interviews analysed by qualitative content analysis.		37.67; PHQ-9 6.13, 3.96, 4.44 vs 4.78, 6.59, 5.61; FACIT-Sp 23.52, 25.02, 27.31 vs 24.85, 21.79, 23.06 - the control group deteriorated over time, so the between-group differences reflect both improvement in the intervention group and worsening in the control group. Process evaluation: reminiscence sessions averaged 26.22 minutes (SD 11.53); over 80% of participants were highly interested; over 90% were satisfied with the intervention (patients mean 4.41, SD 0.66; caregivers 4.55, SD 0.60) and 90% were satisfied with the facilitator; about 80% found the sessions and Spiritual Diary useful and would recommend them; only 52% of patients thought it helped alleviate depressive symptoms; no harm was reported. Sensitivity analyses (complete case and last observation carried forward) were consistent in direction. No effect sizes were reported.		intervention group, which they say needs critical consideration, and they conclude that because of the small sample, high attrition and small effects they cannot conclude the therapy is effective in decreasing dignity-related distress. Their own hypotheses I (dignity-related distress) and IV (sustained effects) were not supported; hypotheses II and III were. No minimal clinically important difference was defined and no effect sizes were reported.
11	Wang et al. 2025	Continuity of care	The gap was not measured with a dedicated instrument. It is framed in the background as the absence of continuous and comprehensive medical and psychosocial support for a disease with high recurrence and readmission rates, and as the lack of consensus on what continuity of care should consist of, how long it should last and how it should be evaluated. It was operationalised indirectly through self-management scores, quality of life, depression and anxiety scores, 6-minute walk distance, self-efficacy, left ventricular ejection fraction, cardiovascular complications and nursing satisfaction. Readmission, continuity indices and unmet needs were not measured; the authors acknowledge that effects on rehospitalisation and mortality remain to be investigated.	Not directly measured (self-management, quality-of-life and clinical outcomes treated as indirect evidence)	Multiple	An unnamed self-management scale with three subscales (self-care agency, self-responsibility, self-concept), with no source reference or psychometric data reported; Minnesota Living with Heart Failure Questionnaire (MLHFQ) covering physical activity, emotion and symptoms; Hamilton Depression Scale (HAMD); Hamilton Anxiety Scale (HAMA); 6-minute walk test (6MWT); General Self-Efficacy Scale (GSES, named only in the results text); left ventricular ejection fraction (LVEF) by echocardiography; complication counts extracted from electronic medical records; and an unnamed three-category nursing satisfaction rating (very satisfied / relatively satisfied / dissatisfied). The authors state that validated standardised tools were used and that, where possible, data collection and scoring were done by researchers not involved in the intervention, with objective outcomes taken from the electronic medical record.	Before the intervention; 1 month and 2 months after the intervention (HAMD, HAMA, 6MWT, self-efficacy, LVEF); before and after the intervention with the post time point unspecified (self-management, MLHFQ); within 3 months after the intervention (complications); satisfaction time point not stated.	Self-management, experimental vs control after the intervention: self-care agency 89.41 (SD 5.38) vs 76.47 (SD 3.26), t = 13.799, P < .001 (from 73.39 and 73.42 at baseline); self-responsibility 90.37 (SD 5.73) vs 73.21 (SD 3.71), t = 16.863, P < .001; self-concept 89.34 (SD 5.73) vs 74.26 (SD 3.32), t = 15.276, P < .001; no baseline differences (all P > .83). MLHFQ (figure only, no test statistics): experimental group before to after - physical activity 16.23 to 13.37, emotion 6.29 to 4.68, symptoms 12.39 to 9.59; control group 16.55 to 15.43, 6.94 to 5.87 and 12.44 to 11.62; no difference at baseline, P < .05 after. HAMD: 57.04 (SD 3.23) to 50.68 (SD 3.07) at 1 month and 41.46 (SD 1.83) at 2 months vs control 56.92 to 54.36 and 52.15; t = 5.7604 and 20.7076, both P < .001. HAMA (figure): 56.14 to 47.73 to 40.48 vs 55.26 to 53.89 to 49.77, P < .05 at 1 and 2 months. 6MWT: 337.76 (SD 23.87) to 397.48 (SD 24.46) to 462.53 (SD 26.28) m vs control 337.49 to 363.97 to 417.78 m; t = 6.5051 and 8.0787, both P < .001. Self-efficacy: 23.45 (SD 3.52) to 27.68 (SD 4.19) to 31.53 (SD 6.17) vs 23.52 to 24.97 to 26.79; P = .0013 and .0003. LVEF: 52.37 to 55.63 (SD 3.23) to 59.89 (SD 5.57) vs 52.33 to 53.89 (SD 3.67) to 55.25 (SD 5.21); P = .0191 at 1 month and .0001 at 2 months. Complications within 3 months: 1/45 (2.22%, one arrhythmia, no myocardial	Positive (significant)	Every reported comparison favoured the intervention and reached statistical significance after the intervention, with no null findings anywhere in the paper. Clinical significance was not formally assessed and no minimal clinically important difference was defined, although the between-group gap in 6-minute walk distance at 2 months (462.53 vs 417.78 m) and the complication rates (2.22% vs 15.56%) are large in absolute terms. The uniformly significant results should be weighed against the retrospective single-centre design, the unexplained contradictions in the paper and the absence of confidence intervals,

ID	Author, year	Care gap type	How the gap was measured	Effect on the gap	Outcome category	Measurement instrument(s)	Time points	Results	Direction of effect	Statistical vs. clinical significance
								infarction or sudden cardiac death) vs 7/45 (15.56%: 2 myocardial infarction, 3 arrhythmia, 2 sudden cardiac death); chi-square 4.9390, P = .0263. Nursing satisfaction: 42/45 satisfied (93.3%) vs 31/45 (68.9%); chi-square 8.7752, P = .0031. No confidence intervals, effect sizes or adjusted analyses beyond the propensity score matching are reported.		effect sizes and adjustment.
12	Mallow et al. 2018	Access to care (primary): rural transportation, insurance and work-hour barriers (mean travel 21 miles). Secondary: self-management support, monitoring, preventive care/health maintenance, equity (low income, health disparity) and fragmentation of care (non-integrated technology)	The gap was not measured directly. Care was delivered remotely, and effects were assessed indirectly via biophysical outcomes (blood glucose, systolic and diastolic BP, body weight, BMI) captured through Bluetooth devices transmitted to the study database; feasibility and acceptability (direct access measures) reported in separate publications; no direct access-gap metric quantified in this paper	Not directly measured (uncontrolled within-group changes in biophysical outcomes treated as indirect evidence)	Clinical/biophysical outcomes (glucose, blood pressure, weight, BMI); psychosocial outcomes (loneliness, depression - baseline only); quality of life (baseline only); process/implementation outcomes (feasibility and acceptability reported separately)	Bluetooth glucometer, blood-pressure monitor and scale (biophysical, transmitted to database); SF-36v2 Medical Outcomes Trust Short Form-36 Health Survey (validated; 0-100, higher = better); UCLA Loneliness Scale version 3 (validated; 20-80, higher = more lonely); Patient Health Questionnaire (PHQ-9) (validated depression screen); BMI calculated from height and weight	Baseline (at enrollment) and 12 weeks (post-intervention); surveys planned at both but post-intervention response too low (n=9) so SF-36, UCLA and PHQ-9 reported at baseline only	Within-group paired-samples t-tests (biophysical): Blood glucose pre M=201.93±88.29 vs post M=146.79±60.67 (units reported as mmol/L), t(28)=4.54, p<0.001, Cohen's d=0.84, n=29; Systolic BP pre M=134.24±15.57 vs post M=118.93±12.56 mmHg, t(28)=6.29, p<0.001, d=1.16, n=29; Diastolic BP pre M=88.79±10.72 vs post M=83.62±8.06 mmHg, t(28)=4.31, p<0.001, d=0.80, n=29; BMI pre M=36.77±8.70 vs post M=35.05±4.39, t(29)=2.16, p=0.04, n=30; Body weight pre M=217.97±44.64 vs post M=207.43±43.89 lbs, t(29)=2.08, p=0.053 (NS), d=0.36, n=30; correlations: age with systolic BP r=0.37 p=0.04, age with diastolic BP r=-0.44 p=0.02, number of chronic illnesses with diastolic BP pre r=-0.43 p=0.01	Uncontrolled pre–post change (significant within-group reductions in glucose, systolic and diastolic BP and BMI; weight not significant)	Statistically significant within-group reductions for glucose, systolic BP, diastolic BP and BMI (all p<0.05) with medium-to-large effect sizes (d=0.80-1.16); weight not statistically significant (p=0.053); no MCID defined and clinical significance not formally assessed; no between-group comparison (uncontrolled)
13	Xie et al. 2025	Continuity of care (primary; authors describe fragmented follow-up). Secondary: self-management support, psychosocial support, education/health-literacy and monitoring gaps (standard care lacks a structured self-care framework)	The gap was not measured directly. Related outcomes: self-care ability (SCHFI); medication adherence (MMAS-4); anxiety and depression (SAS, SDS); HF-related hospital readmissions from electronic medical records and monthly telephone follow-ups; all compared between groups	Not directly measured (significant between-group improvements in self-care, adherence, mood and HF readmissions treated as indirect evidence)	Self-management/self-care (primary); medication adherence; psychosocial/mental health (anxiety, depression); healthcare utilisation (readmission)	Self-Care of Heart Failure Index (SCHFI) v6.2 (maintenance 10 items, management 6, confidence 6; standardised 0-100, higher = better); Morisky Medication Adherence Scale-4 (MMAS-4) (0-4, higher = better adherence); Self-Rating Anxiety Scale (SAS) (20 items, index 20-80, higher = more anxiety); Self-Rating Depression Scale (SDS) (20 items, 20-80, higher = more depression); HF readmissions from electronic medical records	Baseline (before) and after the 12-week intervention for SCHFI, MMAS-4, SAS and SDS; HF-related readmissions tracked over 12 months post-enrolment	SCHFI significantly higher in study group post-intervention (p<.05; numeric values only in Figure 1); MMAS-4 distribution significantly better in study group (p<.05; more scored 4, fewer scored 0); SAS least-squares change control -5.71 (95% CI -7.72 to -3.69) vs study -15.88 (-17.89 to -13.86), between-group difference 10.17 (7.35 to 13.00), p<.001 (SAS before control 42.3 SD 8.17, study 43.9 SD 7.19; after control 37.3 SD 7.96, study 27.4 SD 6.81); SDS LS change control -6.59 (-8.52 to -4.65) vs study -12.17 (-14.11 to -10.23), difference 5.58 (2.87 to 8.30), p<.001; HF readmissions 0 times control 16 (31%) vs study 34 (65%), 1-3 times control 31 (60%) vs study 16 (31%), more than 3 times control 5 (10%) vs study 2 (4%), Fisher's exact p=.001	Positive (significant)	Statistically significant across all reported outcomes (self-care, adherence, SAS, SDS, readmissions); clinical significance not assessed and no MCID defined
14	Fu et al. 2026	Continuity of care (primary). Secondary: coordination gap (fragmented hospital–community–home care), follow-up, self-management support, monitoring (lack of real-time monitoring)	The gap was not measured directly. Related outcomes: health-related quality of life (SF-36) and self-management skills (AHSMSRS) compared between groups over time; platform usability, perceived usefulness and perceived economic impact via a custom questionnaire	Not directly measured (between-group improvements in quality of life and self-management treated as indirect evidence)	Quality of life (primary); self-management; implementation/usability outcomes; economic (perceived cost) outcomes	SF-36 Health Survey (Chinese-validated; 8 domains, 0-100, higher = better; PCS and MCS); Adult Health Self-Management Skill Rating Scale (AHSMSRS; 38 items, three dimensions, converted 0-100, higher = better); Barthel Index (BADL, baseline); Functional	Baseline, 3 months and 6 months post-discharge (HRQoL and self-management); platform user experience assessed once at	Total HRQoL time effect p<0.001, time x group p=0.010; at 6 months intervention 104.78±19.95 vs control 101.57±17.08 (between-group p=0.026); PF at 6 months 59.32±27.15 vs 53.04±27.86 (p=0.026); RP 59.11±34.15 vs 48.69±33.12 (p=0.002); RE 77.95±28.64 vs 68.94±34.89 (p=0.021); VT 60.00±17.93 vs 56.18±15.08 (p=0.003); PCS 56.99±21.75 vs	Mixed (significant 6-month between-group advantage for total HRQoL, PF, RP, RE, VT, PCS, MCS and self-	Statistically significant for the primary outcome (HRQoL) and several secondary outcomes; mixed across domains; clinical significance not

ID	Author, year	Care gap type	How the gap was measured	Effect on the gap	Outcome category	Measurement instrument(s)	Time points	Results	Direction of effect	Statistical vs. clinical significance
		and access gaps (limited non-urban community resources)				Activities Questionnaire (FAQ, IADL, baseline); custom author-developed, non-validated Platform User Experience Questionnaire (usability, usefulness, economic impact)	the end of the intervention	52.24±19.88 (p=0.012); MCS 68.62±18.44 vs 64.59±17.48 (p=0.008); no significant between-group difference for GH, BP, SF or MH (MH 6-month p=0.423); self-management total time x group p<0.001, 6 months 78.94±9.99 vs 72.98±14.59 (p<0.001), behaviours 75.33±11.43 vs 68.68±15.38 (p<0.001), cognition 85.89±11.42 vs 78.24±16.86 (p<0.001), environment not significant (6-month p=0.100); usability 96.35% found it easy to use, 94.79% effective, 70.83% agreed it reduced overall costs; most-used feature health education 88.02%	management; no difference for GH, BP, SF, MH and self-management environment)	assessed and no MCID defined
15	Wong et al. 2010	Continuity of care (primary). Secondary: self-management/medication-adherence and monitoring gaps; unmet care needs of an under-studied CKD/ESRF population (authors note disease management has neglected this group)	The gap was not measured directly. Related outcomes: non-adherence via a modified Dialysis Diet and Fluid Non-adherence Questionnaire (patient self-report; diet, fluid, dialysis, medication; frequency and degree); quality of life (KDQOL-SF); satisfaction (La Monica-Oberst scale); symptom/complication control (chart-validated plus blood chemistry); health-service utilisation (hospitalisations and emergency-room visits from hospital computer records)	Not directly measured (selective between-group improvements in adherence, quality of life and satisfaction treated as indirect evidence)	Self-management/medication adherence; quality of life; patient satisfaction; clinical (symptom/complication control, blood chemistry); healthcare utilisation	Modified Dialysis Diet and Fluid Non-adherence Questionnaire (DDFQ; self-report; 7-day recall; degree 0-4; CVI=1, ICC 0.98); Kidney Disease Quality of Life-Short Form (KDQOL-SF; Chinese-validated; ICC 0.81-0.96); La Monica-Oberst Patient Satisfaction Scale (28 items; alpha 0.91, ICC 0.97); symptom/complication control (chart validation, ICC 0.93; blood chemistry - urea, creatinine, sodium, potassium, phosphate, albumin); health-service utilisation (hospital records)	Baseline O1, 7 weeks O2 (one week after the 6-week programme) and 13 weeks O3 (six weeks after completion)	Diet degree between-group F=4.19, p=0.04 (O2 study 0.49 vs control 0.98, t=2.90, p=0.00; O3 non-significant); CAPD degree within-group F=4.68, p=0.02, between-group non-significant overall but O3 study 0.00 vs control 0.08, t=2.07, p=0.04; diet days, fluid degree (F=8.68, p=0.00) and medication degree (F=8.45, p=0.00) significant within-group only, no between-group difference; KDQOL sleep between-group F=12.19, p=0.00 (study higher at O2 63.8 vs 47.9, t=-3.40, p=0.00 and O3 64.3 vs 48.1, t=-3.81, p=0.00; baseline imbalance confirmed by ANCOVA); KDQOL symptom between-group F=5.12, p=0.03 (O2 78.1 vs 69.8, t=-2.58, p=0.01; O3 79.4 vs 72.7, t=-2.23, p=0.03); staff encouragement O2 90.8 vs 81.9, t=-2.44, p=0.02; overall health O2 56.9 vs 48.1, t=-2.32, p=0.02; satisfaction O2 study 77.12 vs control 72.26, t=-2.66, p=0.01 (O3 non-significant; between-group F=2.50, p=0.12); no significant differences in blood chemistry or utilisation (hospitalisation O2/O3 study 4.1%/20.4% vs control 6.1%/22.4%; ER O2/O3 study 4.1%/16.3% vs control 6.1%/16.3%)	Mixed (study group better on diet-degree and CAPD-degree non-adherence, KDQOL sleep and symptom, staff encouragement, overall health and satisfaction; no difference on most other outcomes, blood chemistry and service use)	Statistically significant between-group differences on selected outcomes (diet-degree and CAPD-degree non-adherence, sleep, symptom, staff encouragement, overall health, satisfaction at O2); clinical significance not formally assessed (no MCID) and no change detected in clinical blood chemistry
16	Ratanawongsa et al. 2012	Health literacy / information gap	Measured directly (provider-reported). PCP questionnaire assessing 13 barriers to diabetes care, including limited English proficiency, inadequate self-management resources, medication management problems, fragmented care, difficulty monitoring patients between visits, and lack of multidisciplinary support.	Partially reduced: provider-reported barriers related to limited English proficiency and medication management were significantly more often overcome with automated telephone self-management support (ATSM)	Multiple	PCP survey assessing patient activation, goal setting, quality of diabetes care and barriers to care.	1 year post-intervention.	Patient activation was higher in ATSM vs usual care (ES +0.41, p = 0.01) and in GMV vs usual care (ES +0.31, p = 0.05). Quality of care was rated higher for ATSM vs usual care (OR 0.38 for poor/bad care rating, p < 0.01). PCPs more frequently reported that ATSM than GMV helped overcome barriers related to limited English proficiency (82% ATSM vs 44% GMV, p = 0.01) and medication management (80% ATSM vs 53% GMV, p = 0.01); the barrier item was asked only for intervention-arm patients (no usual-care comparison).	Positive (significant)	Statistically significant improvements in provider-perceived patient activation, quality of care and selected barriers to care; no direct patient clinical outcomes reported in this analysis. Barrier-related differences compare two active interventions (ATSM vs GMV) and

ID	Author, year	Care gap type	How the gap was measured	Effect on the gap	Outcome category	Measurement instrument(s)	Time points	Results	Direction of effect	Statistical vs. clinical significance
				than with group medical visits (GMV) (82% vs 44% and 80% vs 53%); the barrier item was asked only for intervention-arm patients, so no comparison with usual care was possible; PCPs were not blinded						therefore do not establish barrier reduction relative to usual care; all outcomes are unblinded provider perceptions collected once, post-intervention.
17	Riegel et al. 2002	Continuity of care	The gap was not measured with a dedicated instrument. It was operationalised using health service utilisation indicators, including hospitalisation rates, readmissions, hospital days, costs, emergency department visits and patient satisfaction after hospital discharge.	Not directly measured (reductions in HF hospitalisation, readmission and costs treated as indirect evidence)	Multiple	Hospital administrative and financial records (hospitalisations, readmissions, hospital days, costs), physician office records (outpatient resource use), and a patient satisfaction survey.	3 months and 6 months after hospital discharge.	HF hospitalisation rate reduced by 45.7% at 3 months (0.17 vs 0.31, p=0.03) and by 47.8% at 6 months (0.21 vs 0.41, p=0.01). HF readmission rate reduced by 35.9% at 6 months (17.7% vs 27.6%, p=0.04). HF hospital days reduced by 46.4% at 6 months (1.1 vs 2.1 days, p=0.03). Multiple readmissions reduced by 43% (13.1% vs 22.8%, p=0.03). HF inpatient costs reduced by 45.5% ($1192 vs $2186, p=0.04). Patient satisfaction was higher in the intervention group (22.88 vs 21.66, p=0.01). No significant differences in emergency department or physician office visits.	Positive (significant)	Both statistically and clinically significant. Reductions in hospitalisations, readmissions and costs were substantial and exceeded the estimated intervention cost ($443 per patient).
18	Akkus & Akdemir 2011	Unmet needs	The gap was not measured using a dedicated instrument. It was operationalised through symptom assessment, nursing diagnoses based on NANDA, functional problems, psychosocial needs, and quality-of-life outcomes.	Not directly measured (quality of life treated as indirect evidence)	Quality of life (PROM)	Multiple Sclerosis Quality of Life Scale (MSQOL-54); Symptom Evaluation Form.	Baseline and 4 months (after eighth home visit).	MSQOL-54 Physical Health Composite improved significantly in the intervention group compared with the control group (change score: -8.59 vs 2.61, p = 0.02). Mental Health Composite improved more in the intervention group, but this was not statistically significant (p = 0.06). Significant improvement was observed for role limitation due to emotional problems (p = 0.04).	Positive (significant)	Statistically significant improvement in physical quality of life and emotional-role functioning; improvement in mental quality of life showed a positive but non-significant trend.
19	Kizawa et al. 2020	Health literacy / information gap	Measured directly through goal-of-care preferences, CPR preferences, designation of a health care proxy, CPR knowledge scores, and readiness for advance care planning.	No change: no significant between-group differences; both groups improved within groups	Multiple	Goal-of-care preference questionnaire; CPR preference assessment; health care proxy designation; CPR Knowledge Questionnaire (score 0–5); Advance Care Planning Readiness Scale (score 1–4).	Baseline and immediately post-intervention.	No statistically significant differences between intervention and control groups were observed for the primary outcome (goal-of-care preference) or secondary outcomes. However, significant improvements were observed within groups. Health care proxy designation increased from 29% to 65% in the intervention group and from 22% to 52% in the control group (both p < 0.001). CPR knowledge improved from 3.7 ± 1.1 to 4.6 ± 0.7 in the intervention group and from 3.4 ± 1.2 to 4.5 ± 0.7 in the control group (both p < 0.001). Readiness for advance care planning improved significantly in both groups. In the intervention group, the proportion of participants who did not	No significant between-group difference	No significant between-group effects were observed. Nevertheless, statistically significant within-group improvements were found in health care proxy designation, CPR knowledge, readiness for advance care planning, and CPR preferences. Because similar improvements

ID	Author, year	Care gap type	How the gap was measured	Effect on the gap	Outcome category	Measurement instrument(s)	Time points	Results	Direction of effect	Statistical vs. clinical significance
								want CPR increased from 55% to 67% in a terminal condition (p = 0.003) and from 67% to 80% in a bedridden condition (p < 0.001).		occurred in both groups, they cannot be attributed to the intervention; clinical relevance was not formally assessed.
20	Nualchaem et al. 2026	Fragmented care	The gap was not measured with a dedicated instrument. It was conceptualised as fragmented health services, inadequate continuity of care and insufficient self-management support for older adults with multimorbidity, and was evaluated indirectly through self-management, functional status, blood sugar and blood pressure outcomes.	Not directly measured (self-management, functional status and clinical outcomes treated as indirect evidence)	Multiple	Modified Diabetes Self-Management Questionnaire (DSMQ); functional status assessment (ADL); blood sugar measurements; blood pressure measurements.	Baseline and week 12.	At week 12, the intervention group demonstrated significantly greater improvements in self-management, functional status, blood sugar and blood pressure compared with the comparison group (all p < 0.001).	Positive (significant)	Statistically significant improvements were observed across all measured outcomes. Clinical relevance was supported by improved blood glucose and blood pressure control alongside better self-management and functional status.

Note. Effect on the gap was classified according to the criteria described in Section 2.3 of the manuscript: “Reduced”, a direct measure of the targeted (primary) care gap showed a statistically significant between-group improvement; “Partially reduced”, a direct measure improved on some but not all indicators, only within groups or in uncontrolled designs, or only on provider perceptions or in a comparison between two active interventions without a usual-care comparator; “No change”, a direct measure showed no significant between-group difference; “Not directly measured”, the gap itself was not assessed and only related clinical, behavioural, psychosocial or utilisation outcomes were reported (treated as indirect evidence). The targeted gap was measured directly in four of the 17 primary-study reports (Meekaew et al., Boult et al., Ratanawongsa et al. and Kizawa et al.); it was reduced in two (Meekaew et al., Boult et al.), partially reduced in one (Ratanawongsa et al.) and unchanged in one (Kizawa et al.). Direction of effect refers to the primary outcome and uses the categories defined in Table 2a of the manuscript. IDs 2–4 are systematic reviews, which are not included in the primary-study counts.

Supplementary material Table S4. Extended data-extraction matrix (Part 4 of 4): synthesis—key findings, barriers and facilitators, limitations, and preliminary themes.

ID	Author, year	Key finding relevant to the review question	Barriers and facilitators	Limitations (per authors)	Preliminary code / theme
1	Meekaew et al. 2025	A 12-week nurse-led self-care programme delivered through a messaging application already in everyday use improved knowledge, self-care agency, self-care behaviour and blood pressure, and produced small but clinically relevant improvements in A1C and microalbuminuria. Supports the nurse's role in bridging patients' self-care deficits where specialist diabetes educators are scarce.	Facilitators: LINE is already widely used in Thailand; structured 12-hour nurse training; theory-driven design (Orem); development with the ADDIE model and pilot testing with nurses and patients; real-time nurse-patient communication; patient handbook as supplement. Barriers: low digital literacy among older participants, who required family support; functional restrictions of the free version of LINE OA; nurse daily workload and limited numbers of diabetes care and education specialists in Thai community settings. Nurses recommended digital literacy orientation at programme start.	Most participants were elderly with low educational levels; small trial, which may affect sustainability of the intervention; short 12-week duration without extended follow-up. Authors call for larger samples and longer follow-up.	Bridging self-care deficit through nurse-led mHealth support
2	Zhu et al. 2015	Nursing action taken early in the admission, rather than at the point of discharge, is associated with fewer readmissions, shorter readmission stays and lower mortality - but it does not shorten the index admission itself, so the benefit lies after	Facilitators: structured nurse-led protocol, initiation within 48 hours, multidisciplinary support, longer follow-up intervals (benefit appeared only from 6 months onward). Barriers: discharge planning services still in their infancy in mainland China with limited governmental policy support and no national	Only English-language publications were included; the number of trials per meta-analysis ranged from 2 to 10 with combined samples of 182-3376, limiting precision; substantial heterogeneity (I2 = 66%) for readmission; possible publication bias for length of	Early discharge planning as a continuity mechanism

ID	Author, year	Key finding relevant to the review question	Barriers and facilitators	Limitations (per authors)	Preliminary code / theme
		discharge rather than within it. Continuity of care itself was not measured directly.	programmes or guidelines; wide variation in how services are organised across health systems; lack of standardised reporting of methods and results, which prevented meta-analysis of cost outcomes.	stay; inherent variability in patients' underlying diseases; the search may have missed important reports, and unpublished or non-English reports could have produced different results.	
3	Shaw et al. 2014	Giving nurses protocol-based authority to titrate and initiate medication improves intermediate cardiovascular outcomes in outpatients with chronic conditions, showing that extending nursing scope of practice - rather than adding a new service - may help narrow the gap between recommended and delivered care where primary care clinicians are scarce, although the gap itself was not measured directly.	Facilitators: written protocols and algorithms (provided in all but one study), study-specific nurse training, physician supervision, the nurse-led clinic structure and authority to initiate as well as titrate medication. Barriers: the literature lacked detailed descriptions of interventions, intervention intensity, nurses' education and supervision, protocol adherence and resource use; variation in nurses' scope of practice between countries limits transferability; no evidence from inpatient settings.	The inclusion criterion of medication titration may have excluded studies in which nurses practised autonomously in other ways; inpatient settings were not included; the literature lacked detailed descriptions of interventions and protocols; 11 of 18 studies were conducted outside the USA, which may limit applicability to US practice; only RNs were studied, with no reports on licensed practical nurses; reported outcomes varied across studies and contributed to unexplained variability.	Extended nursing scope as a route to guideline-concordant care
4	Le Berre et al. 2017	Structured transitional care reduces mortality, readmissions and readmission days for older patients with chronic disease, but the improvement in service use is not accompanied by any measurable gain in quality of life. Critically for this review, the subgroup analysis found that interventions led by a nurse did NOT produce better results than other professional configurations - the effect appears to lie in the structure of the follow-up rather than in the discipline delivering it.	Facilitators identified by the authors for large-scale implementation: an educational component, initial contact within one week of discharge, multidisciplinary coordination, continuity through repeated contacts with the same professional (about seven contacts), 24/7 telephone availability, follow-up of at least six months, and pharmacist involvement. Interpersonal continuity was especially valued by older people and produced patient activation and better adherence. Barriers: the complexity of implementing a multi-component intervention, organisational readiness to adopt it, the flexibility required of managers, and the need for sustainable funding and payment models such as bundled payments.	Some trials could not be pooled because outcomes were reported inconsistently; different quality of life scales prevented full pooling and required narrative synthesis; data were sparse at early (under 3 months) and later (18 months or more) time points, which may explain the difficulty in establishing significance there; blinding of participants and personnel was impossible given the nature of the interventions.	Transitional care reduces service use but not quality of life
5	Boult et al. 2008	Embedding a specially trained registered nurse inside a primary care practice doubled the odds that multimorbid older patients rated their chronic care as high quality, with the largest gains in goal setting and care coordination - precisely the dimensions that define a care gap - and improved physicians' satisfaction with communicating with and educating patients and caregivers.	Facilitators: a structured nurse education programme ending in a practicum with simulated patients; a web-accessible shared electronic health record; deliberate integration into the practice through orientation, observation and interaction with staff; a defined caseload of 50-60 patients; and close physician collaboration. Nurses reported high job satisfaction with the flexibility of working hours and the model of care. Barriers: a limited consent rate (37.8% of eligible patients), reliance on self-reported data and a suboptimal physician response rate at one-year follow-up (67%).	Small number of nurses (seven); geographic scope limited to the urban mid-Atlantic USA; reliance on self-reported data; limited consent rate; suboptimal physician response rate at the one-year follow-up survey, which could have biased the observed results.	Nurse embedded in primary care improves perceived quality of chronic care
6	Schenker et al. 2015	An oncology nurse-led care management model was feasible and acceptable and was perceived by patients, caregivers and oncologists to improve palliative care processes; notably, nurses without prescribing privileges influenced treatment decisions in 53% of oncologist check-ins. The design can support claims about feasibility and process, not about patient outcomes.	Facilitators: use of existing clinic nursing staff rather than new posts; a detailed evidence-based intervention manual with checklists and scripts; mandatory oncologist follow-up after every visit; biweekly supervision reviewing audiorecordings; strong clinic leadership support; adjusted staffing so that CONNECT patients were always assigned to the CONNECT nurse; permitting telephone visits for patients too ill to attend. Barriers: the nurse felt overwhelmed by faster than anticipated enrolment; patient deaths during follow-up; a minority of patients too sick to attend clinic; nurses lack prescribing privileges, which the authors identify as an anticipated criticism.	Single-site study; no control arm, so efficacy cannot be established; the community clinic population was 96% Caucasian, so a racially or ethnically diverse population could not be enrolled; small sample; expected loss to follow-up from mortality in a population with advanced cancer.	Nurse-delivered primary palliative care as a substitute for scarce specialty access
7	Leff et al. 2009	Embedding a registered nurse trained in Guided Care within primary care to assess, coordinate, monitor and support multimorbid older patients was associated with 15-37% lower use of hospital, skilled nursing facility, emergency and home healthcare and an estimated net saving of $75,000 per nurse per year during the first 8 months, but no difference reached statistical significance. The paper therefore shows a direction	Facilitators: completion of a Guided Care Nursing course before deployment; the nurse embedded in the practice alongside 2 to 5 physicians and office staff; a bounded caseload of 50-60 patients; a standardised protocol of 8 defined services with a written evidence-based care guide; claims-based targeting of the highest-risk patients. Barriers: the early implementation phase, with nurses, physicians, patients and family caregivers still becoming	Randomisation by pods within practices could have allowed contamination of the usual care group, reducing measured differences; the analysis assumed a common treatment effect across pairs of pods and was not powered to evaluate heterogeneity between pods; the use of national CMS payment rates and mid-Atlantic nurse salaries limits the applicability of the	Nurse-led case management in primary care: non-significant trend toward lower acute and post-acute service use and net cost saving

ID	Author, year	Key finding relevant to the review question	Barriers and facilitators	Limitations (per authors)	Preliminary code / theme
		of travel rather than a demonstrated effect on the utilisation consequences of fragmented care, and the authors expect the effect to strengthen as the model matures.	familiar with the model and 6-8 months needed to build caseloads; possible contamination of usual care pods within the same practice; possible variation in how effectively individual pods implemented the model; geographic variation in service costs and nurse salaries. Barriers and facilitators were not studied systematically.	reported net savings to areas with different costs; emergency care, durable medical equipment, tests and treatments could not be included in the aggregate cost analysis because average cost estimates were unavailable; the observed differences did not meet the traditional criterion for statistical significance, and these preliminary 8-month findings have wide confidence intervals and should be interpreted with caution.	
8	Rechenberg et al. 2021	A language concordant health coaching intervention delivered by nurse and nurse practitioner students to Latinx immigrants with type 2 diabetes and limited English proficiency was feasible (82% response rate, 6% attrition, 4.6 of 6 sessions completed) and acceptable (high PSCC satisfaction), and was accompanied by significant within-group improvements in A1c, depressive symptoms and anxiety that were absent in the control group. Language concordance, comprehension and unmet information needs were not measured, and no between-group tests were performed, so the effect on the communication and health-literacy gap remains untested.	Facilitators: coaches proficient in the patients' preferred language; telephone delivery with short sessions (mean 18 minutes); training of nurse and nurse practitioner students in language concordant diabetes care; fidelity support through bilingual clinical instructors who reviewed call notes, listened to random calls and gave feedback; recruitment through Spanish-language brochures, introduction by primary care providers and a trained bilingual promotor; participant-led, emergent calls with collaborative goal setting; financial incentives; frequent and quick responses from coaches. Barriers: competing life demands in the target community, which the authors give as the reason only 4.6 of 6 sessions were completed on average; the need for a mean of 1.94 calls to reach a participant for each session; limited English proficiency and low income in the population. Participants discussed their own barriers and facilitators to self-management during the calls, but these were not reported in this paper.	The sample of 17 (two fewer than the 19 indicated by the power analysis) was sufficient to establish feasibility and acceptability but insufficient for all of the mental health measures or for any complex analyses; the sample is too small to generalise to the population, and a larger, adequately powered trial accounting for covariates such as socio-demographics, time since diagnosis and medication adherence is the logical next step; the positive outcomes may in part be due to the frequent and quick responses of the health coaches, since attention alone often has a positive impact on desired outcomes.	Language concordant nurse-delivered coaching targeting the communication and health-literacy gap; feasibility established, efficacy not yet tested
9	Schraeder et al. 2008	Adding a registered nurse case manager to primary care practices, with a home assessment, joint care planning with the physician, at least monthly monitoring, patient education and active involvement at care transitions, did not change whether chronically ill older people were admitted to hospital or used emergency care at all, but among those admitted it significantly reduced repeat admissions (34% lower odds), total admissions and bed days over 36 months. Fragmentation itself was not measured; these utilisation findings are indirect evidence and suggest an effect after a hospital episode rather than on initial access to acute care, and the intervention did so at a cost that was at worst budget neutral.	Facilitators described by the authors: locating the nurse case manager inside the practice with about five physicians; clearly identified roles, scope of practice and agreed communication channels; the nurse demonstrating clinical competence, availability for consultation and accountability; nurse participation in clinic visits, brief e-mail updates, impromptu conversations and scheduled team conferences; e-mail alerts and monthly feedback reports to the teams; the initial face-to-face home assessment, which built a personal relationship and revealed home circumstances the physician did not see; education delivered in small, tailored segments with comprehension checked at follow-up; involvement of family in care planning; the longitudinal design, as benefits were expected to accrue over time; implementation within a multi-specialty group practice and a vertically integrated network, which the authors believe makes the model more effective. Barriers: fragmentation of medical and community services; time constraints in physician office visits and their focus on acute rather than comprehensive needs; patients being unable or unwilling to raise concerns with their physician; system differences between the two geographically distinct sites; the authors imply the model may work less well in traditional, non-integrated primary care practices.	The treatment-comparison group design may not have controlled for system differences between the two sites that could have influenced outcomes; a propensity score was calculated to account statistically for the non-randomised design, and the same analysis repeated in the patients not screened as high risk found no significant differences in utilisation or cost, which the authors take as support that the observed effects were due to the intervention. The analyses were limited to variables collected by patient self-report; access to actual pre-enrolment utilisation data, had it been available, might have changed how patients were classified into risk categories. The results may not be generalisable to patients in other geographical areas or healthcare markets, since the sample came from urban and rural central Illinois.	Nurse case management embedded in primary care reduces repeat hospitalisation and bed days but not initial admissions or emergency use; cost-neutral
10	Xiao et al. 2022	A culturally adapted, family-involving dignity therapy targeted the unmet existential and spiritual needs of Chinese patients receiving chemotherapy for lung cancer: at one week it significantly reduced existential distress and depressive symptoms and improved spiritual well-being relative to an attention control, and spiritual well-being remained significantly better at four weeks. Unmet needs were not	Facilitators: involving the family caregiver, which promoted communication, reinforced the patient's self-value and also benefited caregivers; systematic cultural adaptation using the MRC framework, two reviews and a prior qualitative study; a written question protocol giving both parties hints about what to recall; brief sessions (mean 26 minutes) that the authors judge not time-consuming and practicable by trained nurses; flexible timing	A relatively small sample size and a high attrition rate at both T1 and T2, which increased the risk of over- or underestimating the effects, although sensitivity analyses were used to test robustness. Culture strongly affects the sense of dignity, so generalisability to other cultures or countries may be limited and cultural modification would be needed elsewhere. The	Culturally adapted, family-involving psychosocial intervention targeting unmet existential and spiritual needs; effects largely fade within four weeks

ID	Author, year	Key finding relevant to the review question	Barriers and facilitators	Limitations (per authors)	Preliminary code / theme
		measured directly, and the related outcomes improved only partly - the symptom-related and peace-of-mind components of dignity distress were untouched, the effects on distress and depression had faded by week four, and the authors themselves judge the size of the change in dignity-related distress too small to claim effectiveness. Relevant caveat for a review of nurse-delivered care: the therapy was delivered by a trained researcher, with nurse-led delivery proposed only as an implication for practice.	and location, including bedside curtained space, private room and WeChat video for the sharing session; fast, accurate automated transcription giving a 48-hour turnaround for the Spiritual Diary; a facilitator trained by the developer of dignity therapy; a fidelity checklist and process evaluation; high reported satisfaction. Barriers: high attrition from physical deterioration, chemotherapy side effects, referral to other hospitals, loss of contact and travel distance; reluctance to answer the two questions about what patients wanted their family to remember and what they still needed to say, which led patients towards thoughts of death and making a will while they were still on active treatment; 11 patients initially considered the Spiritual Diary unnecessary; one newly diagnosed patient could answer only one question in 8 minutes and withdrew because of unbearable psychological distress, so facilitators must be prepared to stop the intervention and arrange professional psychological support; low awareness among patients and caregivers of the importance of psychosocial care, which the authors say hinders participation; the requirement for an available family caregiver; the next chemotherapy cycle falling around the week-four assessment, increasing distress and influencing the evaluation.	family-oriented approach excluded patients who could not identify a significant family caregiver, meaning the intervention cannot help highly vulnerable patients who have poor family support but an acute need for dignity preservation. Outcomes were evaluated with questionnaires answered subjectively by patients, which could have introduced bias despite the use of well-validated scales. Only 14 patients and 11 family members completed post-intervention interviews, which limited deeper exploration of participants' perceptions.	
11	Wang et al. 2025	A structured continuity of care programme delivered by a multidisciplinary team containing a nurse - personalised education, psychological support, medication management, outpatient, remote and telephone monitoring, and self-management tools - was associated with significantly better self-management, quality of life, depression and anxiety scores, walking distance, self-efficacy, ejection fraction, complication rates and satisfaction than routine care in patients with chronic heart failure. The continuity gap was addressed through sustained post-discharge contact rather than through any measured index of continuity, and the retrospective design and the internal inconsistencies in the report mean these associations cannot support a causal claim.	Barriers and facilitators were not studied or discussed. Facilitators can only be inferred from the intervention design: a multidisciplinary team spanning cardiology, nursing, pharmacy, nutrition and mental health; education tailored to each patient's cognitive level and educational background; provision of practical tools such as heart failure diaries and self-monitoring devices; and multiple contact channels (outpatient, remote monitoring, telephone). Barriers: NR.	First, as a retrospective controlled study conducted in a single centre, generalisability may be limited, and multicentre studies with larger samples are needed to validate the findings. Second, although propensity score matching was used to balance baseline characteristics, unmeasured confounding may still exist. Third, follow-up was relatively short, so long-term effects on clinical outcomes such as mortality and rehospitalisation remain to be investigated. Fourth, some outcome assessments relied on self-reported questionnaires and may be subject to response bias.	Structured multidisciplinary continuity of care after discharge associated with better self-management and function; evidence quality low
12	Mallow et al. 2018	A nurse practitioner-led web-based mHealth intervention (mI SMART) delivered remotely to rural, low-income adults with multiple chronic conditions was associated with significant short-term improvements in glucose, blood pressure and BMI; access to care itself was not measured, and the single-group pre/post design provides only uncontrolled within-group evidence	Barriers: small sample and no control group; commonly cited rural barriers of patient willingness, ability and internet access (which authors argue were not observed here); non-integration of data with existing EHRs; limited reimbursement for technology; workflow uncertainty; high baseline loneliness and obesity complicating outcomes. Facilitators: NP willingness to implement the technology; combined multiple technologies with live technical support; provided devices, tablet and internet data; usability enhancements, in-person training, recorded demonstrations and written instructions; automated reminders and immediate feedback; theoretical grounding (Quality Health Outcomes Model, Chronic Care Model) [authors discussed]	Small sample size; lack of a comparison/control group; convenience sample; participants not assessed for being native to a rural population; community-dwelling adults within a limited age range; findings should not be generalised to other rural populations or clinics	NP-led remote mHealth targeting the access gap (access not measured); reduced biophysical risk without controlled comparison; self-management support via remote monitoring; uncontrolled pre/post feasibility evidence; addressing rural equity/access gap; unmet psychosocial need (loneliness) alongside chronic disease
13	Xie et al. 2025	A 12-week nurse-delivered Orem-based self-care programme (education plus telephone reinforcement) significantly improved self-care ability, medication adherence and mood and reduced HF-related readmissions versus standard care in	Barriers: self-care ability is not static and may decline over time without ongoing support; diverse patient care needs (education level, health status, family circumstances) complicate individualised interventions given limited health-care resources.	Self-care ability may decline over time, so ongoing support is needed (12-month follow-up only); diverse care needs of HF patients complicate delivery of personalised education given resource limitations;	Enhanced self-management through theory-based nursing; reduced hospital

ID	Author, year	Key finding relevant to the review question	Barriers and facilitators	Limitations (per authors)	Preliminary code / theme
		older patients with chronic heart failure; continuity of care itself was not measured directly	Facilitators: structured Orem-based protocol; trained multidisciplinary team including a nurse educator and clinical psychologist; family/caregiver involvement; weekly telephone reinforcement and self-care checklists [authors discussed]	single-hospital and older-only sample noted as future-research context	utilisation; medication adherence support; improved psychosocial wellbeing; continuity through structured telephone follow-up; individualised nursing support
14	Fu et al. 2026	A nurse-led WeChat-based digital follow-up platform targeting continuity of care produced, in older post-discharge adults, significantly greater 6-month gains in overall quality of life and self-management than standardised nurse telephone follow-up, though several quality-of-life domains showed no between-group difference; continuity itself was not measured directly	Barriers: variable digital literacy among older users (interactive modules less used); small number of clusters; short follow-up; performance bias from lack of blinding; mixed evidence and unclear long-term cost-effectiveness. Facilitators: WeChat mini-program requiring no separate installation; user-centred nurse-led design; caregiver involvement via linked accounts; automated reminders and monitoring reducing manual workload; integration of hospital-community-patient modules [authors discussed]	Relatively small number of clusters (limits cluster-level power and precision of effect estimates); single-city recruitment (limited generalisability); 6-month follow-up only (long-term effects unclear); outcomes primarily self-reported (reporting bias); no blinding of participants or implementers (performance bias)	Nurse-led digital follow-up targeting continuity (continuity not measured); stronger self-management capacity; digital-literacy constraint on interactive features; perceived cost reduction; mixed quality-of-life outcomes
15	Wong et al. 2010	A 6-week nurse-led disease management programme using a specialist-plus-general-nurse telephone model improved diet and CAPD adherence, some quality-of-life aspects (sleep, symptom) and satisfaction for chronic kidney disease patients, but effects were selective and short-lived and did not change health-service utilisation or blood chemistry	Barriers: small sample and short follow-up limiting detection of biochemical effects; sustaining effects requires continuous application of the intervention. Facilitators: protocol-driven skill-mix model optimising specialist and general nursing resources; specialist nurse as resource person; structured Omaha-based assessment; mutual goal-setting and a sustained nurse-patient relationship; expert-panel-validated protocols [authors discussed]	Subjects confined to CAPD renal patients, limiting generalisability to other chronic-disease groups; small sample size and relatively short follow-up meant effects on outcomes needing higher power or longer time (e.g. blood chemistry) could not be demonstrated	Care coordination via nurse skill-mix; medication/diet adherence support; selective and short-lived adherence gains; improved satisfaction and selected quality-of-life aspects; no change in utilisation; resource-optimising disease management
16	Ratanawongsa et al. 2012	A nurse-supported telehealth self-management programme improved provider-perceived patient activation, quality of diabetes care, and provider-reported language (limited English proficiency) and medication-management barriers among vulnerable adults with diabetes; barrier differences were observed only relative to group medical visits (no usual-care comparison) and were based on unblinded provider perceptions.	Facilitators: multilingual automated system, nurse follow-up, integration with primary care, high patient engagement, and tailored self-management support. Barriers: limited English proficiency, medication-management difficulties, fragmented care, and difficulty monitoring patients between visits.	Primary Care Providers (PCPs) were not blinded to the intervention assignment; potential recall bias; outcomes based on surveys; limited sample size; and findings derived from a single urban safety-net health system may not be generalizable.	Nurse-supported telehealth: provider-perceived gains in activation and quality of care; communication barriers addressed more often than with group visits (active-comparator, provider-reported evidence).
17	Riegel et al. 2002	A standardised nurse-led telephone case-management programme significantly reduced heart failure hospitalisations, readmissions, inpatient costs and resource use, while increasing patient satisfaction; continuity of care itself was not measured directly.	Facilitators: decision-support software, standardised protocol, intensive follow-up, early post-discharge contact, ongoing nurse training, communication with physicians, patient education materials. Barriers: high intervention intensity required, dependence on nurse expertise, potential variability in patient severity and engagement.	Physicians, rather than patients, were randomised; possible selection bias; limited power for outpatient resource-use outcomes; physicians may have become aware of allocation; actual nurse time was estimated rather than measured.	Nurse-led telephonic case management targeting continuity of care reduces hospital utilisation.
18	Akkus & Akdemir 2011	Structured nurse-led home visits based on NANDA nursing diagnoses improved quality of life in people with multiple sclerosis, particularly physical functioning; unmet home-care and psychosocial needs were targeted but not measured directly.	Facilitators: individualized NANDA care plans, regular home visits, patient and family education, continuity of support, nurse expertise in MS, psychosocial counselling. Barriers: limited home-care services in Turkey, absence of multidisciplinary team involvement, high disability burden (high EDSS scores), disease uncertainty and progression, limited community support resources.	Small sample; short intervention period; no multidisciplinary team; high disability level of participants; uncertainty and variability of MS progression; home visits delivered by a single nurse.	NANDA-based home nursing improves quality of life in MS; unmet home-care needs targeted but not measured.
19	Kizawa et al. 2020	Knowledge, readiness for advance care planning and health care proxy designation improved in both the visual-material	Facilitators: structured nurse-led counselling, trained nurses, individualised communication, visual educational materials,	Pilot study; recruitment via an online commercial database, with possible selection bias; predominantly	Nurse-led advance care planning counselling: no

ID	Author, year	Key finding relevant to the review question	Barriers and facilitators	Limitations (per authors)	Preliminary code / theme
		and the verbal nurse-led advance care planning groups, with no significant between-group differences; the added value of visual materials was not demonstrated, and the within-group improvements cannot be attributed to the intervention.	advance care planning discussions, improved patient understanding of CPR and treatment options. Barriers: limited prior experience with advance care planning, cultural reluctance to discuss end-of-life decisions, potential family influence on decision-making, difficulty imagining future health states.	well-educated male participants; visual materials were not developed through a fully rigorous process; some outcome measures were modified and not formally validated in the Japanese context; no long-term follow-up.	added effect of visual materials over verbal counselling (directly measured information gap unchanged).
20	Nualchaem et al. 2026	A nurse-led integrated care model that combines case management, multidisciplinary collaboration, home visits and self-management support improved self-management, functional status and chronic disease control among older adults with multimorbidity; fragmentation of care itself was not measured directly.	Facilitators: nurse case management, multidisciplinary team involvement, integrated care pathways, home visits, family and community support, self-monitoring application, regular feedback and goal setting. Barriers: fragmented health services, poor continuity of care, low health literacy, limited family/community support, difficulties maintaining self-management in an urban ageing population.	Not reported.	Nurse-led integrated care targeting fragmented care strengthens self-management in older adults with multimorbidity.

Supplementary material Table S5. Preferred Reporting Items for Systematic Reviews and Meta-Analyses (PRISMA 2020) Checklist.

Section and Topic	Item #	Checklist item	Location where item is reported
**TITLE**			
Title	1	Identify the report as a systematic review.	p. 1
**ABSTRACT**			
Abstract	2	See the PRISMA 2020 for Abstracts checklist.	p. 2
**INTRODUCTION**			
Rationale	3	Describe the rationale for the review in the context of existing knowledge.	pp. 2-3
Objectives	4	Provide an explicit statement of the objective(s) or question(s) the review addresses.	p. 3
**METHODS**			
Eligibility criteria	5	Specify the inclusion and exclusion criteria for the review and how studies were grouped for the syntheses.	pp. 3-5; Table 1
Information sources	6	Specify all databases, registers, websites, organisations, reference lists and other sources searched or consulted to identify studies. Specify the date when each source was last searched or consulted.	p. 4
Search strategy	7	Present the full search strategies for all databases, registers and websites, including any filters and limits used.	p. 4; Supplementary Table S7
Selection process	8	Specify the methods used to decide whether a study met the inclusion criteria of the review, including how many reviewers screened each record and each report retrieved, whether they worked independently, and if applicable, details of automation tools used in the process.	p. 4
Data collection process	9	Specify the methods used to collect data from reports, including how many reviewers collected data from each report, whether they worked independently, any processes for obtaining or confirming data from study investigators, and if applicable, details of automation tools used in the process.	p. 5

Section and Topic	Item #	Checklist item	Location where item is reported
Data items	10a	List and define all outcomes for which data were sought. Specify whether all results that were compatible with each outcome domain in each study were sought (e.g. for all measures, time points, analyses), and if not, the methods used to decide which results to collect.	pp. 4-6; Suppl. Table S3
	10b	List and define all other variables for which data were sought (e.g. participant and intervention characteristics, funding sources). Describe any assumptions made about any missing or unclear information.	p. 5; Suppl. Tables S1-S2
Study risk of bias assessment	11	Specify the methods used to assess risk of bias in the included studies, including details of the tool(s) used, how many reviewers assessed each study and whether they worked independently, and if applicable, details of automation tools used in the process.	p. 7 (partial)
Effect measures	12	Specify for each outcome the effect measure(s) (e.g. risk ratio, mean difference) used in the synthesis or presentation of results.	Not explicitly specified; pp. 7-9; Suppl. Table S3
Synthesis methods	13a	Describe the processes used to decide which studies were eligible for each synthesis (e.g. tabulating the study intervention characteristics and comparing against the planned groups for each synthesis (item #5)).	pp. 3-6
	13b	Describe any methods required to prepare the data for presentation or synthesis, such as handling of missing summary statistics, or data conversions.	p. 5
	13c	Describe any methods used to tabulate or visually display results of individual studies and syntheses.	pp. 7-9; Suppl. Tables S1-S4
	13d	Describe any methods used to synthesize results and provide a rationale for the choice(s). If meta-analysis was performed, describe the model(s), method(s) to identify the presence and extent of statistical heterogeneity, and software package(s) used.	p. 6
	13e	Describe any methods used to explore possible causes of heterogeneity among study results (e.g. subgroup analysis, meta-regression).	p. 6; pp. 16-18
	13f	Describe any sensitivity analyses conducted to assess robustness of the synthesized results.	Not performed
Reporting bias assessment	14	Describe any methods used to assess risk of bias due to missing results in a synthesis (arising from reporting biases).	Not formally assessed; pp. 17-18
Certainty assessment	15	Describe any methods used to assess certainty (or confidence) in the body of evidence for an outcome.	p. 7 (no formal certainty framework)
**RESULTS**			
Study selection	16a	Describe the results of the search and selection process, from the number of records identified in the search to the number of studies included in the review, ideally using a flow diagram.	pp. 6-7; Figure 1
	16b	Cite studies that might appear to meet the inclusion criteria, but which were excluded, and explain why they were excluded.	Figure 1, p. 6 (full-text exclusion reported)
Study characteristics	17	Cite each included study and present its characteristics.	pp. 7-8; Table 2; Suppl. Table

Section and Topic	Item #	Checklist item	Location where item is reported
			S1
Risk of bias in studies	18	Present assessments of risk of bias for each included study.	pp. 10-15; Tables 3-6
Results of individual studies	19	For all outcomes, present, for each study: (a) summary statistics for each group (where appropriate) and (b) an effect estimate and its precision (e.g. confidence/credible interval), ideally using structured tables or plots.	pp. 8-9; Suppl. Table S3
Results of syntheses	20a	For each synthesis, briefly summarise the characteristics and risk of bias among contributing studies.	pp. 7-8, 10-15
	20b	Present results of all statistical syntheses conducted. If meta-analysis was done, present for each the summary estimate and its precision (e.g. confidence/credible interval) and measures of statistical heterogeneity. If comparing groups, describe the direction of the effect.	N/A: no statistical synthesis; pp. 8-9
	20c	Present results of all investigations of possible causes of heterogeneity among study results.	pp. 16-18 (narrative only)
	20d	Present results of all sensitivity analyses conducted to assess the robustness of the synthesized results.	Not performed
Reporting biases	21	Present assessments of risk of bias due to missing results (arising from reporting biases) for each synthesis assessed.	Not formally assessed
Certainty of evidence	22	Present assessments of certainty (or confidence) in the body of evidence for each outcome assessed.	pp. 10-15, 17-18 (overall only)
**DISCUSSION**			
Discussion	23a	Provide a general interpretation of the results in the context of other evidence.	pp. 16-18
	23b	Discuss any limitations of the evidence included in the review.	pp. 10-15, 17-18
	23c	Discuss any limitations of the review processes used.	pp. 17-18
	23d	Discuss implications of the results for practice, policy, and future research.	pp. 16-18
**OTHER INFORMATION**			
Registration and protocol	24a	Provide registration information for the review, including register name and registration number, or state that the review was not registered.	p. 3
	24b	Indicate where the review protocol can be accessed, or state that a protocol was not prepared.	Not reported
	24c	Describe and explain any amendments to information provided at registration or in the protocol.	p. 3 (no amendments after registration)
Support	25	Describe sources of financial or non-financial support for the review, and the role of the funders or sponsors in the review.	p. 18
Competing	26	Declare any competing interests of review authors.	p. 19

Section and Topic	Item #	Checklist item	Location where item is reported
interests			
Availability of data, code and other materials	27	Report which of the following are publicly available and where they can be found: template data collection forms; data extracted from included studies; data used for all analyses; analytic code; any other materials used in the review.	p. 18; Suppl. Tables S1-S6

Supplementary material Table S6. Citation matrix of overlap between included systematic reviews and primary studies.

Primary study included in the present review	Zhu et al. [40]	Shaw et al. [41]	Le Berre et al. [42]
Meekaew et al.			
Boult et al			
Schenker et al.			
Leff et al			
Rechenberg et al			
Schraeder et al			
Xiao et al.			
Wang et al.			
Mallow et al.			
Xie et al.			
Fu et al.			
Wong et al	X		
Ratanawongsa et al.			
Riegel et al.			X
Akkus & Akdemir			
Kizawa et al.			
Nualchaem et al.			

Note. X indicates that the primary study was included in the corresponding systematic review. Two of the 16 unique primary studies included in the present review (Wong et al. and Riegel et al.) were also included in at least one systematic review. No primary study was included in more than one review. Overlap among the three systematic reviews

themselves was not quantified, because the complete sets of primary studies included in each review were not mapped; the matrix therefore documents only the overlap between the reviews and the primary studies of the present review.

Supplementary material Table S7. Complete database-specific search strategies as executed, including platforms, searched fields, applied limits, and records retrieved.

**Database**	**Platform (interface)**	**Fields searched**	**Search strategy as executed**	**Limits applied**	**Date of final search**	**Records retrieved (before deduplication)**
PubMed	PubMed (U.S. National Library of Medicine)	All Fields (no field tags); phrases in quotation marks	("chronic disease" OR "chronic illness" OR "chronic condition" OR "long-term condition" OR "noncommunicable disease" OR multimorbid OR comorbid) AND ("nursing care" OR "nursing practice" OR "nursing intervention" OR "nursing management" OR "nurse-led" OR "nurse-managed") AND ("care gap" OR "gap in care" OR "unmet need" OR "unmet healthcare need" OR "unmet health care need" OR "missed care" OR "care deficit" OR "fragmented care" OR "continuity of care" OR "access to care" OR "barrier to care" OR "quality of care") AND ("patient outcome" OR "quality of life" OR "quality of care" OR satisfaction OR self-management OR "self-management")	English language; no publication-date limits	15 July 2026	189
MEDLINE	EBSCOhost	Default EBSCOhost fields (no field codes); Boolean/phrase search mode	("chronic disease*" OR "chronic illness*" OR "chronic condition*" OR "long-term condition*" OR "noncommunicable disease*" OR multimorbid* OR comorbid*) AND ("nursing care" OR "nursing practice" OR "nursing intervention*" OR "nursing management" OR "nurse-led" OR "nurse-managed") AND ("care gap*" OR "gap* in care" OR "unmet need*" OR "unmet healthcare need*" OR "unmet health care need*" OR "missed care" OR "care deficit*" OR "fragmented care" OR "continuity of care" OR "access to care" OR "barrier* to care" OR "quality of care") AND ("patient outcome*" OR "quality of life" OR "quality of care" OR satisfaction OR self-management OR "self-management")	English language; no publication-date limits	15 July 2026	257
CINAHL Ultimate	EBSCOhost	Default EBSCOhost fields (no field codes); Boolean/phrase search mode	("chronic disease*" OR "chronic illness*" OR "chronic condition*" OR "long-term condition*" OR "noncommunicable disease*" OR multimorbid* OR comorbid*) AND ("nursing care" OR "nursing practice" OR "nursing intervention*" OR "nursing management" OR "nurse-led" OR "nurse-managed") AND ("care gap*" OR "gap* in care" OR "unmet need*" OR "unmet healthcare need*" OR "unmet health care need*" OR "missed care" OR "care deficit*" OR "fragmented care" OR "continuity of care" OR "access to care" OR "barrier* to care" OR "quality of care") AND ("patient outcome*" OR "quality of life" OR "quality of care" OR satisfaction OR self-management OR "self-management")	English language; no publication-date limits	15 July 2026	301
Web of Science Core Collection	Clarivate	Topic (TS: title, abstract, author keywords, Keywords Plus)	TS=(("chronic disease*" OR "chronic illness*" OR "chronic condition*" OR "long-term condition*" OR "noncommunicable disease*" OR multimorbid* OR comorbid*) AND ("nursing care" OR "nursing practice" OR "nursing intervention*" OR "nursing management" OR "nurse-led" OR "nurse-managed") AND ("care gap*" OR "gap* in care" OR "unmet need*" OR "unmet healthcare need*" OR "unmet health care need*" OR "missed care" OR "care deficit*" OR "fragmented care" OR "continuity of care" OR "access to care" OR "barrier* to care" OR "quality of care") AND ("patient outcome*" OR "quality of life" OR "quality of care" OR satisfaction OR self-management OR "self-management"))	English language; no publication-date limits	15 July 2026	228

Database	Platform (interface)	Fields searched	Search strategy as executed	Limits applied	Date of final search	Records retrieved (before deduplication)
Scopus	Elsevier	TITLE-ABS-KEY (title, abstract, keywords)	TITLE-ABS-KEY(("chronic disease*" OR "chronic illness*" OR "chronic condition*" OR "long-term condition*" OR "noncommunicable disease*" OR multimorbid* OR comorbid*) AND ("nursing care" OR "nursing practice" OR "nursing intervention*" OR "nursing management" OR "nurse-led" OR "nurse-managed") AND ("care gap*" OR "gap* in care" OR "unmet need*" OR "unmet healthcare need*" OR "unmet health care need*" OR "missed care" OR "care deficit*" OR "fragmented care" OR "continuity of care" OR "access to care" OR "barrier* to care" OR "quality of care") AND ("patient outcome*" OR "quality of life" OR "quality of care" OR satisfaction OR self-management OR "self-management"))	English language; no publication-date limits	15 July 2026	213

Note. No controlled-vocabulary terms (MeSH, CINAHL Subject Headings) were used; the same free-text search string was applied in all databases, with truncation symbols (asterisks) removed in PubMed. Records retrieved are reported before deduplication (total n = 1188; 519 duplicates removed; 669 records screened). The reference lists of all included reports were also screened manually; no additional studies were identified.
